# Protein corona formed on silver nanoparticles in blood plasma is highly selective and resistant to physicochemical changes of the solution†
†Electronic supplementary information (ESI) available: Supporting Information 1: Experimental details; figures and tables; protein abundance and corresponding sigmoid fit for all of the differentially abundant proteins detected in pH and temperature perturbation experiments. Supporting Information 2: Complete list of quantified proteins in all LC–MS runs in pH and temperature experiments, including protein metadata (Uniprot accession number, sequence coverage, GO annotation, number of identified peptides, *etc.*) Quantitative data provided as log-converted LFQ abundancies. Supporting Information 3: Results of lessening analysis. See DOI: 10.1039/c8en01054d


**DOI:** 10.1039/c8en01054d

**Published:** 2019-03-18

**Authors:** Vladimir Gorshkov, Julia A. Bubis, Elizaveta M. Solovyeva, Mikhail V. Gorshkov, Frank Kjeldsen

**Affiliations:** a Department of Biochemistry and Molecular Biology , University of Southern Denmark , Odense , Denmark . Email: vgor@bmb.sdu.dk ; Email: frankk@bmb.sdu.dk; b V.L. Talrose Institute for Energy Problems of Chemical Physics , Russian Academy of Sciences , Moscow , Russia; c Moscow Institute of Physics and Technology (State University) , Dolgoprudny , Moscow Region , Russia

## Abstract

Nanoparticles (NPs) in contact with protein-containing media such as biological fluids rapidly acquire a surface layer of proteins, known as the protein corona. The protein composition and structural properties of the protein corona are crucial for NP interactions with living cells. Although much has been learned about the protein corona phenomenon, further elucidation could benefit from extensive quantitative proteomics analysis. Herein we report a comprehensive quantitative characterization (>350 proteins) of the corona that formed on 60 nm silver NPs *via* interaction with human blood plasma, as a function of pH and temperature. By varying the pH and temperature one can access different conformational spaces and charge localizations of the plasma proteins, which in turn provide knowledge pertinent to how the proteome corresponds to binding affinity. Thirty-eight percent of the quantified proteins bind at all temperatures, 47% at all pH values, and of these most persistent proteins, approximately 60% do not significantly change in abundance within the protein corona. Evaluation of 544 protein properties (present in the Kyoto databank) suggests that binding of these proteins to NPs is determined by the extent of hydrophobicity, β-sheet propensity, α-helical structure (and turns), and amino acid composition. Protein binding is promoted by a larger amount of β-sheets, higher hydrophobicity, and a smaller amount of α-helices. Our work enhances researchers' knowledge of a long-standing, vexing aspect of the nano–bio interface.

## Abstract 2

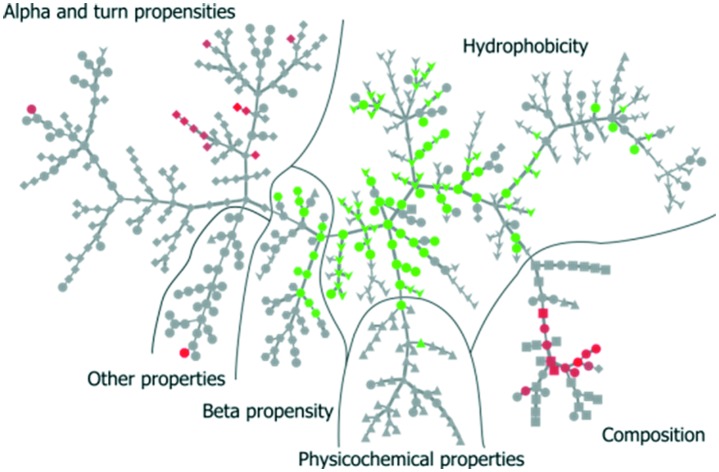
Highlighted are the protein properties determining protein binding to the silver nanoparticle surface.

##

Environmental significance

Proteins adsorbed on the surface of nanoparticles (NPs) in a biological medium (protein corona) are believed to play a key role in the interaction of particles with cells. Hence, assessment of environmental and health effects of nanoparticles is strengthened with a thorough characterization of the corona. The literature shows high selectivity in the formation of the protein corona. From more than 9000 proteins present in blood plasma only 300–500 are reported on NPs. This selectivity calls for the elucidation of the properties responsible for the corona composition. Our study of protein corona on silver NPs highlights the enrichment of proteins having a higher number of β-sheets and hydrophobic regions than that of the background protein repertoire. Our findings could be generalized to other nanomaterials.

## 

Upon contact of a nanoparticle (NP) with protein-containing media such as biological fluids, a layer of proteins (the so-called protein corona) forms on the particle surface. This stabilizes NPs *via* electrostatic and/or steric repulsion.^1^ The corona can be subdivided into a “hard corona”, which contains proteins bound directly to the nanoparticles, and a “soft corona”, which forms mainly by weakly bound proteins, primarily *via* protein–protein interactions.^2^ The protein composition and corona formation have been heavily debated in the literature, as it is considered important for NP interactions with cells and the subsequent biological responses. Examples include whether NPs will be absorbed, excreted, or internalized.^3^,^4^ Also, the protein corona composition is suggested to dictate the interaction of the NP with different cell types.^5^ Therefore, detailed information about the protein corona formation is important for assessing the basic mechanisms behind the cellular interactions with the nanoparticles and their application in biomedical research. Typical methods of in-depth characterization of protein binding to NPs include surface plasmon resonance, size-exclusion chromatography, isothermal titration calorimetry, circular dichroism, IR spectroscopy, NMR spectrometry, and H/D exchange.^2^,^3^,^6^–^9^ These methods allow elucidation of important binding properties, yet are tedious and time-consuming. Hence, low throughput and analysis of only a few selected proteins (conditions that only minimally resemble biological media) are considerable limitations. Alternatively, mass spectrometry-based proteomics approaches can provide unprecedented sensitivity and throughput, allowing for large-scale protein screening analysis. However, they are rarely utilized to describe detailed binding characteristics.

The composition of the protein corona can evolve over time and during the transition from one biological medium to another.^10^ Despite many efforts, there is a lack of deep understanding of the chemical properties which govern protein binding to NPs. The current consensus is that the protein corona forms rapidly within seconds to minutes as a consequence of affinity competition between proteins for NP binding, known as the Vroman effect.^11^,^12^ The final composition of the protein corona is primarily a function of the NP material, size, and surface properties, as well as the protein medium composition and experimental/physiological conditions.^12^–^14^ Xia *et al.* conducted a systematic investigation of the forces involved in small molecule adsorption to NPs and developed a weighted nanodescriptor algorithm to infer the contributions of Coulomb forces, London dispersion, hydrogen bonding, polarizability, and lone-pair interactions.^15^ However, there has been no clear extrapolation of findings based on small molecules to the behavior of large proteins. In principle, all of the aforementioned forces could act on protein binding to NPs, as suggested by many researchers.^16^,^17^

However, these properties are intrinsic to all known proteins and do not clearly correspond to reported NP–protein binding. A survey of the literature shows that approximately 300–500 proteins from human plasma bind to various NPs,^12^,^18^–^21^ which is quite striking considering that the plasma proteome contains more than 9000 different proteins.^22^ Nanoparticles can certainly bind proteins, but based on the small number of reported protein interactions, the likelihood of binding appears to be small (of low affinity) for most proteins. This high degree of selectivity is further substantiated by the fact that the dynamic range of plasma proteins spans 9–10 orders of magnitude.^23^ If most proteins had a similar binding affinity, the protein composition would reflect this impressive protein concentration range, which is not the case. A similar high selectivity toward certain proteins is typically obtained *via* immunoprecipitation or moiety-selective materials such as TiO_2_ for phosphopeptides. It is well known that plasma protein interaction with most widely used resins, such as reverse-phase, hydrophilic interaction liquid chromatography, and strong cation/anion exchange, results in a binding of most abundant proteins,^24^ which is markedly different from what is observed for nanoparticle–protein interaction.

We speculate that one of the reasons for similar results pertaining to protein coronas on various NPs could be due to the strong similarities in experimental design, typically under physiological conditions. However, both the high degree of protein selectivity and the similarity between studies testify that understanding the formation of the protein corona necessitates further attention. For instance, if the charge/conformational space corresponds to protein binding to NPs, then significant pH or temperature changes should have a major impact on the composition and abundance of proteins bound to NPs. We tested this speculation using silver nanoparticles as a model system; our findings suggest that whereas many proteins are exchanged as a consequence of plasma solution perturbation (pH and temperature changes), an unexpectedly large fraction of proteins reside on NPs in all conditions.

## Results and discussion

We formed protein corona on silver nanoparticles (AgNPs) under five different temperatures (4, 17, 30, 41, and 47 °C) and pH values (4.9, 6.1, 6.8, 7.7, and 8.9). Our approach to study the possible effect of protein binding properties as a function of conformational space is based on changing the experimental conditions of the protein solution prior to incubation with NPs, which then reflects the binding affinities of individual proteins to either NPs or proteins already bound to the particles as a function of the available conformational space. We selected experimental conditions to cover the largest possible range at which plasma proteins remain stable (no aggregation), yet are sufficiently broad to enable protein conformational transitions. Protein folding and unfolding equilibria directly correlate to temperature and pH.

We performed each experiment three times in parallel with the corresponding particle-free control. We quantified on average 300 proteins per experimental condition. This number is consistent with the literature.^12^,^18^–^20^ On average, we quantified 87% of the identified peptides and 66% of the identified proteins using label-free quantitation (LFQ). Since we set at least three quantified peptides as the threshold for protein quantitation, the quantification ratio observed at the protein level is lower than that of the peptide level. However, the median number of quantified peptides for each protein was close to 5. A higher number of peptides per protein results in greater reliability of quantitation and may indicate the extent to which the analysis is complete. In other words, the number of proteins identified by a single peptide was low and thus it is unlikely that under-sampling during data-dependent acquisition was the sole cause of not detecting some of the protein corona components.

To avoid non-specific binding, we used low-binding plastic for all steps and we replaced Eppendorf tubes after the incubation and each washing step (see subsequent experimental details). Despite identifying many proteins in the particle-free control samples, their quantities were much less compared to particle-containing samples. The presence of proteins in the particle-free controls can be explained by the extremely high protein concentration in plasma (up to 73 g L^–1^).^25^ Our estimation based on the summed LFQ abundances of all proteins indicates that the total quantity of proteins bound to NPs under the conditions of our experiments was less than 5 μg. Even a negligible transfer of the solution during washing could result in similar quantities in the particle-free controls. Thus, further optimization of our protein corona purification procedure and the use of specially designed equipment for sample preparation could be necessary to mitigate the problem completely. To remove bias in subsequent analyses, we corrected our calculated protein abundances using the particle-free control samples.

**Fig. 1:**
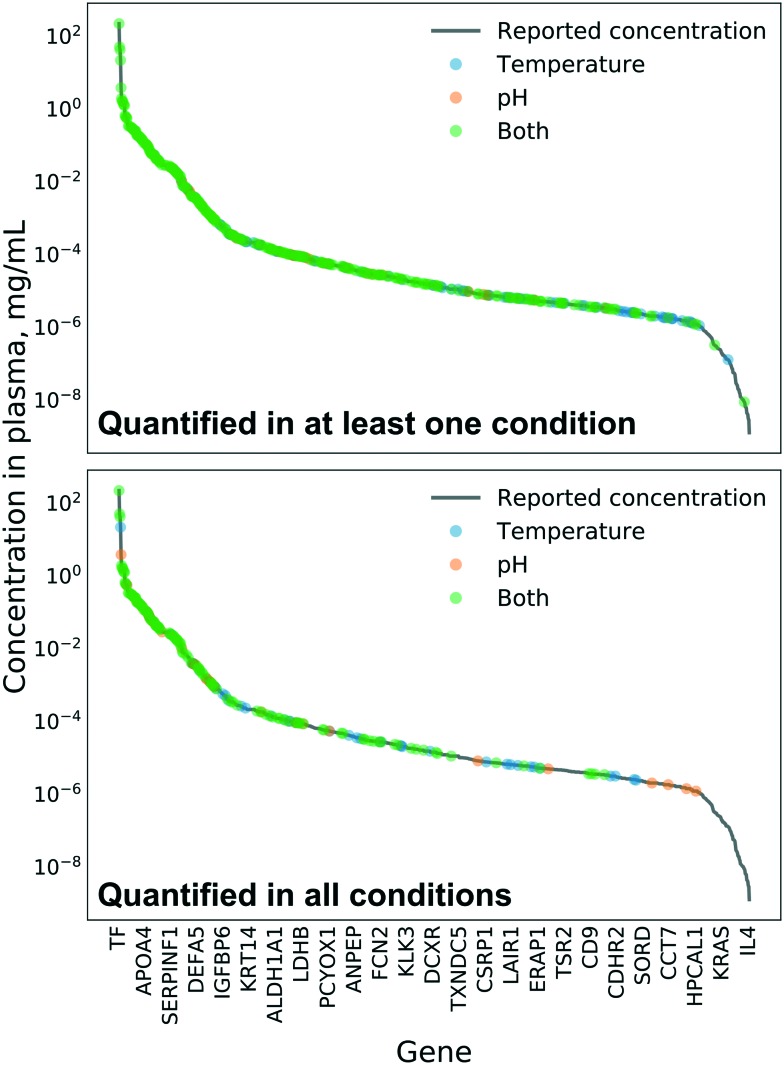
Plasma protein concentration of quantified corona proteins. Concentrations reported in the Plasma Proteome Database.^26^

Fig. 1 shows the proteins quantified in our study and their average abundances reported in the Plasma Proteome Database.^26^ Out of 1276 proteins with reported plasma abundance, we could quantify 404 in at least one condition. From this distribution, it is clear that NPs enrich only a subset of the plasma proteome (the corona consists of a few hundred proteins out of 9000 different proteins reported in the plasma proteome^22^). Despite the enormous distribution of plasma protein abundances, we quantified proteins in the full range of that distribution. The difference in reported plasma abundance between the lowest, oncostatin M (OCM), and the highest, transferrin (TF), among our quantified corona proteins is approximately 10 orders of magnitude. The dynamic range limitation of the mass spectrometer is 4 orders of magnitude, which without enrichment excludes the observation of very low-abundance proteins from plasma samples. The typical result of proteomics analyses of unfractionated plasma shows a strong bias toward high-abundance proteins.^24^

Table 1 lists the 20 most abundant proteins observed in our pH and temperature experiments.

**Table 1:** Twenty most abundant proteins in the pH and temperature experiments

log_10_(abundance)					UniprotID	GeneID	Name
pH 4.9	pH 6.1	pH 6.8	pH 7.7	pH 8.9			
6.34	6.93	6.98	7.30	7.50	P00746	CFD	Complement factor D
6.15	7.00	6.91	7.10	7.23	P07203	GPX1	Glutathione peroxidase 1
7.78	7.13	7.09	7.52	2.45	P05062	ALDOB	Fructose-bisphosphate aldolase B
6.36	6.27	5.95	6.20	6.50	O15143	ARPC1B	Actin-related protein 2/3 complex subunit 1B
6.67	1.32	7.13	7.31	7.11	Q9BQE3	TUBA1C	Tubulin alpha-1C chain
0.00	6.52	7.31	7.53	7.83	P12830	CDH1	Cadherin-1
0.00	6.66	6.68	7.53	7.83	P22692	IGFBP4	Insulin-like growth factor-binding protein 4
0.00	6.36	6.75	7.51	7.94	P24593	IGFBP5	Insulin-like growth factor-binding protein 5
0.00	6.76	6.90	7.25	7.44	P21291	CSRP1	Cysteine and glycine-rich protein 1
8.11	0.00	6.47	6.87	6.64	P0DJI8	SAA1	Serum amyloid A-1 protein
6.87	1.27	6.08	6.25	6.70	Q96IY4	CPB2	Carboxypeptidase B2
0.00	5.90	6.36	6.72	7.65	P01344	IGF2	Insulin-like growth factor II
0.00	6.71	6.35	6.60	6.56	P25774	CTSS	Cathepsin S
0.00	6.25	5.96	6.71	6.61	P04179	SOD2	Superoxide dismutase [Mn], mitochondrial
0.00	6.33	6.14	6.47	6.55	P36542	ATP5C1	ATP synthase subunit gamma, mitochondrial
0.00	5.99	6.20	6.35	6.52	P50552	VASP	Vasodilator-stimulated phosphoprotein
0.00	6.15	6.04	6.33	6.44	Q13576	IQGAP2	Ras GTPase-activating-like protein IQGAP2
0.00	6.49	6.11	6.12	6.20	P43304	GPD2	Glycerol-3-phosphate dehydrogenase, mitochondrial
1.51	1.32	6.95	6.46	6.90	O00187	MASP2	Mannan-binding lectin serine protease 2
6.15	0.54	1.59	7.10	7.27	Q14766	LTBP1	Latent-transforming growth factor beta-binding protein 1

log_10_(abundance)					UniprotID	GeneID	Name
4 °C	17 °C	30 °C	41 °C	47 °C			
7.77	7.45	7.34	7.85	7.05	P10646	TFPI	Tissue factor pathway inhibitor
7.10	6.92	7.20	7.69	6.71	P10720	PF4V1	Platelet factor 4 variant
7.87	6.95	6.57	7.07	6.75	P01344	IGF2	Insulin-like growth factor II
8.39	7.59	7.51	7.46	0.00	P12830	CDH1	Cadherin-1
7.94	7.22	7.30	7.46	0.74	P24593	IGFBP5	Insulin-like growth factor-binding protein 5
7.90	7.04	6.79	6.55	0.00	P05154	SERPINA5	Plasma serine protease inhibitor
7.40	6.40	6.52	7.37	0.29	P00746	CFD	Complement factor D
6.82	7.05	6.80	6.49	0.00	Q9BXN1	ASPN	Asporin
7.18	6.35	6.24	6.56	0.00	Q03591	CFHR1	Complement factor H-related protein 1
2.51	6.83	6.97	7.50	2.11	P17936	IGFBP3	Insulin-like growth factor-binding protein 3
8.20	7.36	7.16	1.42	0.00	P22692	IGFBP4	Insulin-like growth factor-binding protein 4
1.69	6.76	1.82	7.36	5.39	P21291	CSRP1	Cysteine and glycine-rich protein 1
6.27	5.93	1.83	6.84	0.00	P80511	S100A12	Protein S100-A12
0.84	6.62	6.38	0.22	6.63	P04632	CAPNS1	Calpain small subunit 1
7.52	6.61	6.43	0.00	0.10	O00187	MASP2	Mannan-binding lectin serine protease 2
7.72	6.02	6.18	0.00	0.00	P18065	IGFBP2	Insulin-like growth factor-binding protein 2
0.00	0.00	6.24	6.63	6.69	P05164	MPO	Myeloperoxidase
0.00	6.41	6.12	6.69	0.00	P30740	SERPINB1	Leukocyte elastase inhibitor
8.17	7.90	2.28	0.87	0.00	O00602	FCN1	Ficolin-1
0.00	5.12	6.24	6.91	0.43	Q04756	HGFAC	Hepatocyte growth factor activator

The main constituents of the corona are proteins involved in the complement system, blood coagulation, and lipid processing. In general, our findings are in line with previous reports for AgNPs; however, several important observations differ. We found serum albumin, which was reported as one of the major corona components in some studies,^27^ at a much lower concentration herein; however, our results correspond to the most recent studies.^18^,^19^ Similarly, we observed a much lower concentration of kininogen, apolipoprotein E, and immunoglobulins. These proteins are at high abundances in all samples; however, the abundances lessened after applying the particle-free control correction (described in the Methods section). In total, these observations further advocate for an appropriate control experiment for unbiased characterization of NP protein coronas obtained from blood plasma or any complex protein sample, a practice that is not typically applied. The ESI† provides a complete list of the quantified proteins in pH and temperature experiments.

**Fig. 2:**
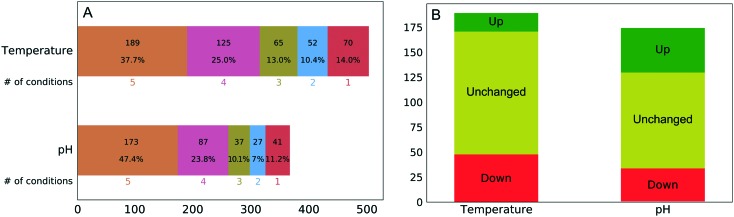
(A) Number of shared protein identities observed between different temperature and pH conditions. (B) Number of persistent proteins and the direction of change (all five conditions), referring to abundance in the protein corona.

The experimental workflow developed allows one to investigate the extent to which the protein corona composition shares similarity at different perturbation conditions. As aforementioned, changes in temperature and pH induce changes both to the predominating conformation and the charge distribution. Fig. 2A shows the number of quantified proteins that were shared between different temperatures or pH values. There was a substantial resistance to forming an entirely new corona when we changed the temperature or pH of the plasma solution prior to NP addition, despite expected major changes in the solution state of plasma proteins.

The percentage of the same protein identities in all five temperature (38%) and pH (47%) conditions was surprisingly high even considering that washing of nanoparticles was performed using phosphate-buffered saline (PBS), which due to its ionic strength could weaken some ionic interactions between proteins and nanoparticles. Although this factor is affecting all experimental conditions it cannot fully account for the observed extraordinary similarity of protein corona.

Our findings suggest that the NP corona consists of two types of proteins: those that are sensitive, and those that are resistant, to temperature or pH perturbations. The latter group deserves deeper investigation in order to assess the quantitative changes, if any, under different conditions.

The thermal shift assay is well known for detecting drug–protein interactions. The underlying principle is the thermodynamic stabilization of proteins as a result of ligand binding.^28^ The variant of this method, thermal proteome profiling, was recently successfully applied in large scale for characterization of drug–protein interactions in living cells.^29^ Herein, we adapted this methodology to measure changes in protein abundances across all experimental conditions.

Out of the 173 corona proteins that we quantified at all pH values, 44 increased in abundance and 33 decreased in abundance as the pH increased from 4.9 to 8.9. Similarly, out of the 189 corona proteins that we quantitated at all temperatures, 18 increased in abundance and 48 decreased in abundance as the temperature increased from 4 to 47 °C (Fig. 2B). The ESI† shows plots representing the protein abundances in each condition and the corresponding sigmoid fits for all proteins. Among a large number of proteins present in the NP protein corona under all of the perturbation conditions, only a minority (35% for temperature and 45% for pH) exhibit changes in abundance. This is surprising, in that the pH (4.9–8.9) and temperature (4–47 °C) changes should induce substantial changes in protein conformation and charge distribution.

For each protein that featured a change in its corona abundance, one can determine the pH or temperature value that corresponds midway between the attached and the detached states. This value should correspond to the structural transition, which we term the “critical pH” and “critical temperature.” As expected, structural changes can both enhance and reduce the binding propensity of proteins to NPs, for example, by changing the accessibility of a specific sequence motif. Likewise, considering a corona formed from a complex protein mixture, one can expect interplay within protein abundances; *i.e.*, the affinity decrease of a particular protein can promote binding of another protein.

**Fig. 3:**
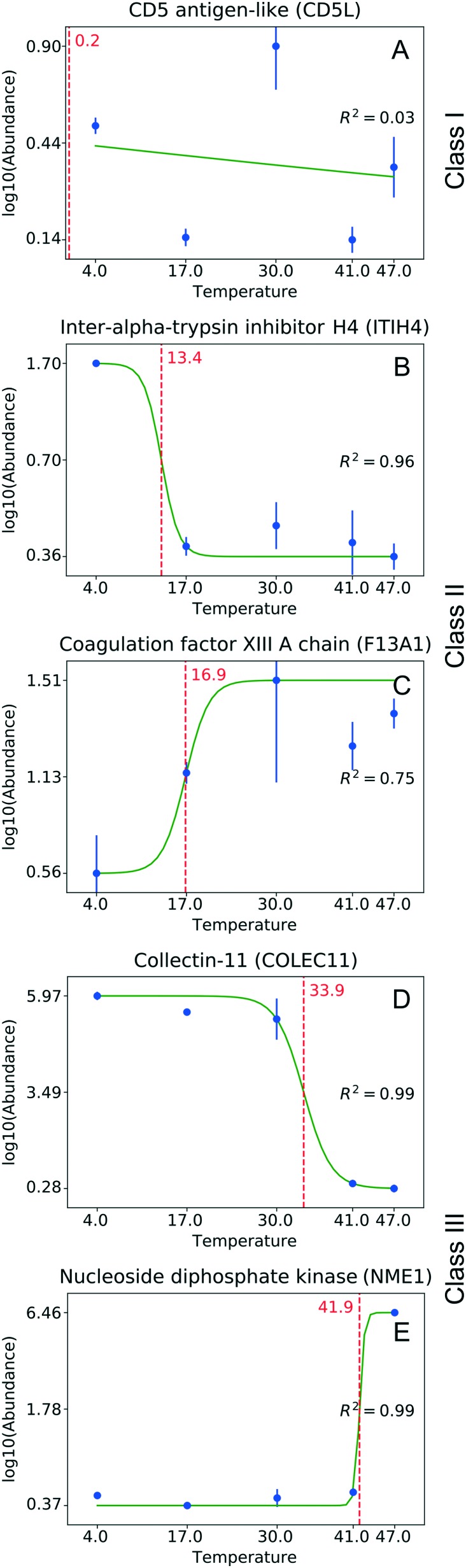
Classes of proteins with respect to their abundance on NPs observed in the temperature experiment. (A) No significant changes observed (Class I); (B, C) binding affinity is reduced or enhanced at low temperatures (<30 °C, Class II); (D, E) binding affinity is reduced or enhanced at higher temperatures (>30 °C, Class III). The green curve shows the sigmoid fit. The red dotted line corresponds to the critical temperature. Whiskers correspond to standard deviation.

One can identify several distinct classes of proteins. Fig. 3 shows some representative examples for the temperature experiment. The largest fraction, Class I, is proteins that do not change their abundance under the studied conditions (Fig. 3A). These proteins are either tolerant to structural change as a function of temperature, or their structural changes do not involve the motif(s) responsible for binding. Class II consists of proteins losing or gaining affinity to NPs at rather low temperatures (<30 °C, Fig. 3B and C). A low critical temperature for those proteins indicates that the change in binding affinity is unlikely to involve extensive thermal denaturation, suggesting that effects other than protein conformation are responsible for binding. Class III includes proteins that either increased or decreased their abundance in the corona at higher temperatures (>30 °C, Fig. 3D and E). For proteins of this class, one can assume a larger degree of thermal denaturation as a key determinant of the observed changes. Similar classes can be identified in the pH experiment as well (the border pH between Class II and Class III is 6.8). Table S1† shows the number of proteins identified in each class.

Savitski *et al.*^29^ reported on melting points corresponding to the temperatures at which a protein loses its native structure and becomes insoluble. The isoelectric point of the protein is the pH value at which a protein undergoes net charge inversion. Fig. S1† shows the correlation of these melting points and isoelectric point values with those of the critical temperature and critical pH determined in our experiments. No correlation can be determined in either case. The absence of a correlation between the pI and our critical pH values suggests that the binding affinity is not solely determined by the global charge (global pI). This contradicts the literature assumption that a protein charge inversion can lead to protein dissociation from NP surfaces.^8^ A possible explanation could be that the charge polarity close to the binding interface of the protein is more pertinent to the affinity. Consequently, the global pI of a protein does not account for the complete distribution of this charge across the protein sequence, presenting the possibility that protein binding to NPs is determined by the local polarity of the protein segment that is involved in this interaction.

The low correlation with the melting temperatures of Savitski *et al.* suggests that structural changes have less of an effect than assumed for this group of proteins. Protein structural alterations may proceed through a number of intermediate steps, each with its own activation energy and/or occurring under specific conditions. The critical parameters that we found during our experiments should be related to a particular transition to some of the intermediate structures.

Since an unexpectedly large percentage (∼60%) of the proteins in the corona of the NPs did not significantly change in abundance under any of the experimental conditions, it is of paramount interest to retrieve further information on common denominators for these sets of proteins. In order to more deeply investigate the relationship between protein binding propensity and protein features, we conducted an extensive analysis using the amino acid index (AAindex) in the Kyoto database.^30^ The AAindex is a database containing hundreds of biological protein properties obtained from empirical data and is presented as numerical indices of amino acids and pairs of amino acids. Our study includes 544 protein properties.

We performed the following test to determine characteristics from the AAindex as pertinent to NP binding. We calculated the numerical value of each property present in the AAindex for all plasma proteins, using sequence and corresponding amino acid indices. Then we examined the distribution of each individual property for the complete population of plasma proteins and proteins that are always present in the corona (*i.e.*, resistant to exchange). We used the dispersion of the distribution as a measure of similarity/dissimilarity between proteins, rationalizing that enrichment of proteins with certain characteristics (properties) should result in narrowing of dispersion compared to the background (complete plasma). Details of the calculation can be found in the experimental section and the ESI.†

Hence, the dispersion will lessen if more proteins with similar values of the property in question are enriched on the surface of the NPs. Analogously, protein properties that are not pertinent to NP adsorption would result in random sampling of proteins and will not change the dispersion by a statistically significant amount. Fig. S2† shows an example of such an analysis. We used bootstrapping to evaluate the level of significance and filtered the results to a false discovery rate (FDR) <0.005.

**Fig. 4:**
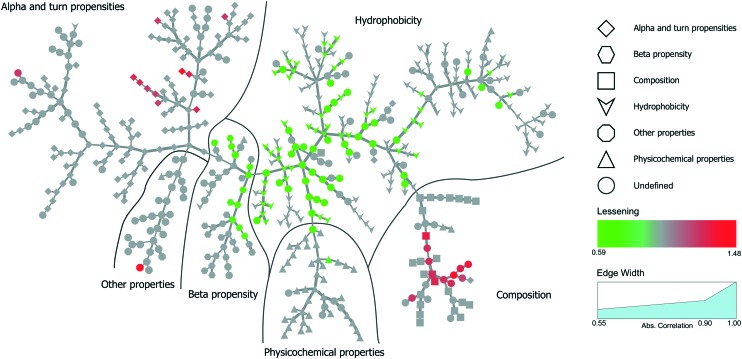
Minimum spanning tree for the temperature perturbation experiment. Properties are divided into six main categories. Individual properties are color-coded in accordance with the decreasing dispersion between proteins bound to NPs under all experimental conditions and proteins found in plasma. Only nodes corresponding to significant changes (FDR <0.005) are colored.

Inspired by Tomii *et al.*,^31^ we created a minimum spanning tree of the 544 properties from the AAindex. This clustered pertinent properties into six categories (composition, physicochemical properties, beta propensity, alpha and turn propensities, hydrophobicity, and other properties). Next, we added the significant dispersion changes to the minimum spanning trees of the temperature experiments and color-coded them in accordance with the decreasing dispersion between proteins bound to NPs and proteins found in plasma (Fig. 4). Fig. S3† shows analogous data for the pH experiment. An interactive visualization of the complete spanning tree can be accessed at https://caetera.github.io/AgNPCorona.

**Fig. 5:**
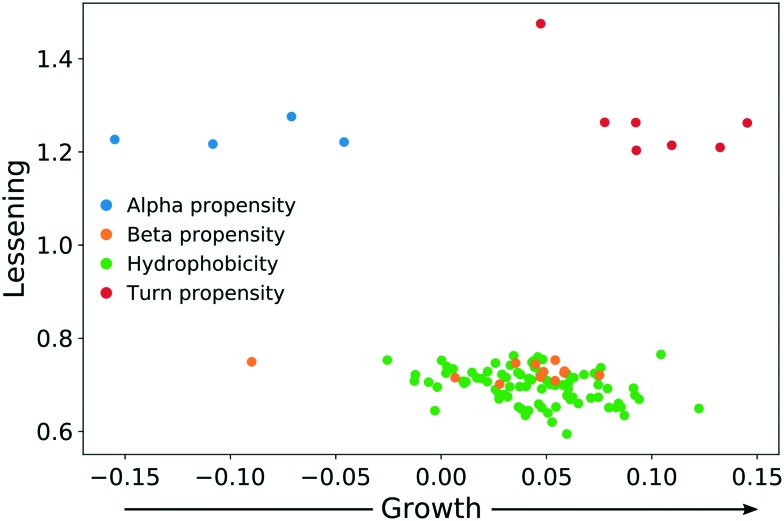
The direction of change for protein properties displaying significant lessening or broadening of distribution in persistent protein fraction.

In both experiments, the proteins remaining in the NP corona despite protein perturbation revealed a clear clustering of protein properties influencing the binding. Our use of two orthogonal perturbation methods increases our confidence in assigning pertinent protein-binding properties. Apart from the properties displaying significant lessening of dispersion (green nodes) we observed properties with significantly wider dispersion (red nodes). Lessening the dispersion is the result of the accumulation of proteins with a similar value of characteristics, while widening could be explained as the tendency of NPs to avoid proteins with a specific value of characteristics. In both cases, one can observe selective behavior highlighting protein properties important for protein to NP interaction. For proteins resistant to exchange despite in-solution perturbation, lessening is observed for hydrophobicity and propensity for β-sheets (green; lesser spread), whereas amino acid composition, α-helical structure, and turn propensities (red; higher spread) result in widening. To obtain further details of the important (resulting in significant dispersion change) properties, we investigated whether the average value of the corresponding properties were larger or smaller for persistent proteins. The plot in Fig. 5 clearly illustrates that persistent proteins have a tendency to prefer a higher amount of β-sheet formation, hydrophobicity, and turn content, while a lower amount of α-helices. This may reflect a greater structural stability of proteins forming more β-sheets than α-helices. This observation is in line with the recent analysis of 8000 proteins showing that β-sheets enhance the structural stability over α-helices.^32^ We observe both properties as important for protein binding. Analogously, protein hydrophobicity is a driving force for strong protein structure formation, since protein folding and stabilization are largely entropy-driven (as per water exclusion from the interior).^33^

From the above results, a picture is emerging that adds to the understanding of protein corona formation. Protein composition in the AgNP corona is a function of affinity and protein structural stability. This affinity is largely related to the protein structural stability, as evidenced by the fact that changing the temperature or pH of the plasma solution results in an exchange of some proteins from the corona. These exchanged proteins are those of lesser affinity under the current physicochemical conditions. This is further substantiated by our observation that proteins which exhibit extraordinary structural stability are retained on the NP surface at all perturbation conditions due to their structure-dependent affinity. We attribute this stability to the enrichment of proteins with higher hydrophobicity and propensity for β-sheets. Our perturbation experiments highlight that the binding interface between a protein and the AgNP surface is likely to be attributable to a specific area/motif in proteins that corresponds to a change in protein affinity upon unfolding.

## Conclusion

We systematically investigated the composition of the blood plasma protein corona formed on 60 nm citrate-stabilized AgNPs as a function of pH and temperature. The quantitative proteomics workflow allowed quantification of 300 proteins (on average) for each studied condition (0.01 FDR on the protein level), providing a comprehensive characterization of the corona proteins. Since we detected non-specific protein binding in the particle-free control experiments, further optimization of the protein corona purification procedure was necessary. Thus, the appropriate correction of the result for the non-specific binding should be employed, especially when working with biological media such as blood plasma that has a high concentration of proteins. An important finding is that for AgNPs, binding is selective and includes only a very small subset of the entire plasma proteome, which raises the question of how much one can manipulate binding to direct other interactions with cells.

Quantitative characterization of corona proteins highlights that whereas many proteins exchange as a consequence of solution perturbation, 38% and 47% bind at all temperatures and pH values, respectively. Furthermore, of the most-persistent proteins, approximately 60% did not change in abundance within the protein corona. Our study of 544 properties of proteins (present in the Kyoto databank) suggests that hydrophobicity, propensity for β-sheets, frequencies of α-helical structure, and amino acid composition are the properties determining the interaction with AgNPs. The persistent fraction is enriched in proteins with higher hydrophobicity, a greater number of β-sheets, and lower content of α-helices. We speculate that these strong protein binders bind to NPs throughout all experimental conditions because these features correspond to extraordinary structural stability. Our findings could also apply to other NPs; however, further studies are necessary given the broad ranges of NP shapes, types, and sizes.

A minority of persistent proteins change their abundance in accordance with the perturbation conditions. For each of those proteins, we determined a characteristic (“critical”) condition, corresponding to the observed change. Our suggested mechanism for this small number of proteins involves a transition into partial denaturation or structural reorganization, changing the accessibility of motifs responsible for protein association.

The current investigation focuses on a single nanoparticle type (60 nm silver nanoparticles with citrate coating). Given the large variety of nanoparticles currently manufactured, there is a possibility that our findings will not be applicable to all of them. Providing exhaustive testing of all possible nanomaterial types is out of the scope of the current study. However, we present a general, robust, scalable, and high-throughput analytical approach for quantitative protein corona characterization that can facilitate standardization and inter-laboratory reproducibility. One can effectively analyze the protein–NP interactions for other nanomaterials and identify those that require further attention. This will refine our knowledge regarding the nano–bio interface, a long-standing, vexing scientific challenge. Further characterization of the conformational changes of these specific proteins and their changes in affinity to the NP surface can be assisted by more specialized mass spectrometry methods, such as H/D exchange, partial hydrolysis, and cross-linking.

## Methods

The ESI† provides detailed experimental procedures.

### Nanoparticle characterization

Silver nanoparticles (AgNPs) with a citrate coating were purchased from Nanocomposix (60 nm, NanoXact, 0.02 mg mL^–1^). The diameter of the particles was measured prior to the experiment using dynamic light scattering (DLS) on a DelsaMax Core instrument (Beckman Coulter, USA): measured diameter 66.4 nm, polydispersity index 0.05.

### Protein corona preparation

Nanoparticles were concentrated *via* centrifugation and mixed with pooled normal human blood plasma (Innovative Research, USA). For pH experiments, the pH of the blood plasma was adjusted by adding an equal amount of phosphate–citrate or Tris-HCl buffer. The pH after mixing was measured with a micro pH-meter (MP220, Mettler Toledo). NPs were incubated with plasma for 4 h at (1) pH 4.9, 6.1, 6.8, 7.7, or 8.9 and a constant temperature of 30 °C; or (2) a temperature of 4, 17, 30, 41, or 47 °C and a constant pH of 7.9. Corona formation was confirmed by DLS (ESI† Fig. S4). NPs bearing a protein corona were separated by centrifugation and washed three times with 1× phosphate-buffered saline (10 mM Na_2_HPO_4_, 2.7 mM KCl, 137 mM NaCl, P4417 (Sigma)), changing the tube after each wash to eliminate non-specific protein binding. Low-binding plastic was used throughout the sample preparation to mitigate further non-specific binding. Each experiment was performed in triplicate with a particle-free control.

### Protein corona isolation and digestion

Corona proteins were eluted by 8 M urea in 25 mM ammonium bicarbonate buffer (ABC), followed by reduction and alkylation *via* dithiothreitol and iodoacetamide, respectively. Proteins were digested with Lys-C (1 : 50, 3 h, 37 °C), followed by trypsin (1 : 50, overnight, 37 °C). Nanoparticles were separated by centrifugation. The samples were concentrated in a SpeedVac and purified using C18 StageTips (Thermo Fisher Scientific). The purified peptides were dried in SpeedVac and stored at –20 °C until LC-MS analysis.

### LC-MS analysis

Samples were analyzed using a Q-Exactive HF mass spectrometer (Thermo Scientific, Bremen, Germany) coupled with an UltiMate 3000 Nanoflow LC system (Thermo Scientific, Germering, Germany). Peptides were concentrated at the trap column (μ-Precolumn C18 PepMap100, Thermo Scientific, 5 μm, 300 μm i.d. 5 mm, 100 Å) and eluted from an analytical column (EASY-Spray PepMap RSLC C18, Thermo Scientific, 2 μm, 75 μm i.d. 500 mm, 100 Å) with a gradient of acetonitrile.

Mass spectrometry measurements were performed using data-dependent acquisition mode (Top 12). MS1 spectra were acquired from 300 to 1400 Th, with a resolving power of 120 000 at *m*/*z* 200. Precursor ions were isolated with the *m*/*z* window of 1.4 Th followed by their fragmentation using higher-energy collision dissociation (HCD). Fragment ions were measured in an Orbitrap mass-analyzer with a resolving power of 15 000 at *m*/*z* 200. Each sample was analyzed in triplicate. The data were deposited in the ProteomeXchange Consortium (http://proteomecentral.proteomexchange.org) *via* the PRIDE^34^ partner repository with the dataset identifiers PXD007648 and 10.6019/PXD007648.

### Data analysis

Mass spectrometry data were converted^35^ to mzML format and searched with MSGF+ (2016.12.12)^36^ against the database of plasma proteins^26^ concatenated with common contaminants and reversed decoy sequences of all proteins (described in detail in the ESI†). Carbamidomethylation of cysteine was used as a fixed modification; variable modifications include methionine oxidation, acetylation of the protein N-terminus, and carbamylation of the peptide N-terminus and lysine. Parent mass tolerance was set to 10 ppm and the instrument was set to Q-Exactive. Identifications of all samples in the same experiment (*i.e.*, pH and temperature perturbation) were merged and validated by Percolator (3.01);^37^ protein FDR was restricted to 0.01. Feature detection, alignment between LC-MS runs, and peptide quantification were performed using OpenMS (2.1.0).^38^ Protein abundance was calculated as a median abundance of the three most abundant peptides (Top3). Proteins having fewer than 3 quantified peptides were excluded. Protein abundances for each replicate were corrected by subtracting the abundances of the same protein found in the corresponding particle-free control sample. The Kyoto AAindex^30^ (v 9.1) was downloaded from the official website (; http://www.genome.jp/aaindex/), and property annotations were extracted from Tomii *et al.*^31^ The dispersion of protein property distributions was calculated as the difference between the 10th and the 90th percentiles. The lessening of the dispersion was calculated as the ratio between the dispersion for the persistent corona fraction and that for the plasma protein database. Significance was estimated by a permutation test and corresponding *p*-values were corrected using the Benjamini–Hochberg method. The minimal spanning tree was calculated using the Kruskal algorithm (as implemented in cySpanningTree 1.1 in Cytoscape 3.6.0), using the absolute value of the Pearson correlation coefficient as a measure of the similarity between protein parameters in the Kyoto AAindex (in a manner similar to that of Tomii *et al.*^31^). Integration of all tools and data analysis was programmed by scripts written in Python (3.6.3). All of our scripts are published *via* GitHub: https://github.com/caetera/AgNPCorona.

## Conflicts of interest

The authors declare no conflicts of interest.

## Supplementary Material

Supplementary information

Supplementary information

Supplementary information
