# Supplementary material for: Protein corona formed on silver nanoparticles in blood plasma is highly selective and resistant to physicochemical changes of the solution
Source: Environ Sci Nano. 2019 Mar 18;6(4):1089–98. doi: 10.1039/c8en01054d (PMC6592156; doi:10.1039/c8en01054d)
Supplement: Supplementary file 1 [file EN-006-C8EN01054D-s001.pdf]

Electronic Supplementary Material (ESI) for Environmental Science: Nano.
This journal is © The Royal Society of Chemistry 2019

Supplementary material for

Protein Corona Formed on Silver Nanoparticles in Blood Plasma is Highly Selective and Resistant to Physicochemical Changes of the Solution

Vladimir Gorshkov[1#*], Julia A. Bubis[2,3#], Elizaveta M. Solovyeva[2,3#], Mikhail V. Gorshkov[2], Frank Kjeldsen[1*]

[1] Department of Biochemistry and Molecular Biology, University of Southern Denmark, Odense, Denmark

[2] V.L. Talrose Institute for Energy Problems of Chemical Physics, Russian Academy of Sciences, Moscow, Russia

[3] Moscow Institute of Physics and Technology (State University), Dolgoprudny, Moscow Region, Russia

# Authors contributed equally

* Corresponding author; Address: Campusvej 55, 5230 Odense M, Denmark; e-mail: vgor@bmb.sdu.dk; frankk@bmb.sdu.dk

Materials and Methods

Protein corona preparation

Nanoparticles (3 mL stock solution) were concentrated by centrifugation (5000g, 10 min, 4°C) followed by the addition of 300 µL of pooled normal human blood plasma (Innovative Research, USA) (41.4 µL of plasma per cm^2 of particle surface). For pH experiments, the pH of blood plasma was adjusted by adding an equal amount of phosphate-citrate buffer solution or Tris-HCl buffer solution (20.7 µL of plasma per cm^2 of particle surface). The pH of the solution after mixing was measured by micro pH-meter (MP220, Mettler Toledo). Nanoparticles were incubated with plasma in thermoshaker (800 rpm) for 4 hours at pH 4.9, 6.1, 6.8, 7.7, 8.9 and constant temperature 30°C or, at temperature 4, 17, 30, 41, 47°C and constant pH 7.9. Corona formation was confirmed by DLS (typical particle diameter after the incubation was ~ 200 nm). Nanoparticles bearing protein corona were separated by centrifugation (10000g, 10 min) and washed three times with 1x PBS (10 mM Na_2HPO_4, 2.7 mM KCl, 137 mM NaCl, P4417 (Sigma)) changing the tube after each wash to eliminate unspecific protein binding. Low binding plastic was used on all sample preparation steps. Each experiment was performed in triplicate with particle-free control.

Protein corona isolation and digestion

Particles with protein corona were placed in 150 µL of 8M urea in 25 mM ammonium bicarbonate buffer (ABC), sonicated for 5 minutes and kept for 10 minutes at room temperature (repeated twice). Proteins were reduced by dithiothreitol (final concentration 10 mM, 1 hour, 37°C) and alkylated by iodoacetamide (final concentration 20 mM, 30 min, room temperature, in dark). The solution was diluted with 115 µL of 25 mM ABC (4M final urea concentration) and digested with Lys-C (1:50, 3 hours, 37°C). Later 900 µL of 25mM ABC (1M final urea concentration) was added to the solution and it was digested with trypsin (1:50, overnight, 37°C). Nanoparticles were removed by centrifugation (15000g, 10 min, 25°C). The sample was concentrated in SpeedVac to the volume of ~300µL and purified by C18 StageTips (Thermo Fisher Scientific). Purified peptides were dried completely in SpeedVac and stored at –20°C until LC-MS analysis.

LC-MS analysis

Samples (~ 1 µg on the column) were analyzed by Q-Exactive HF mass spectrometer (Thermo Scientific, Bremen, Germany) coupled with UltiMate 3000 nanoflow LC system (Thermo Scientific, Germering, Germany). Trap column (µ-Precolumn C18 PepMap100, Thermo Scientific, 5µm, 300µm i.d.5 mm, 100Å) and analytical column (EASY-Spray PepMap RSLC C18, Thermo Scientific, 2 µm, 75µm i.d. 500 mm, 100Å) heated to 50°C were employed for separations. Mobile phases were as follows: (A) 0.1% FA in water; (B) 95% ACN, 0.1% FA in water. Samples were pre-concentrated for 10 min on the trap column at 2%B. Then, peptides were eluted using the following gradient: from 2%B to 20%B in 52.5 min, from 20%B to 32%B in 7.5 minutes at 270 $nL\cdot min^{-1}$ flow rate. The column was washed at 95%B for 10 minutes and equilibrated to the start concentration of mobile phase B.

Mass spectrometry measurements were performed using data-dependent acquisition (DDA) mode (Top 12). Electrospray voltage was set to 2.0 kV. Electrospray capillary temperature was 275ºC. MS1 settings were as follows: mass range from 300 to 1400 Th, resolving power of 120,000 at m/z 200, maximum injection time

was set to 100 ms, the automatic gain control (AGC) for MS1 was 3.0e6. Precursor ions were isolated with the m/z window of 1.4 Th followed by their fragmentation using higher-energy collision dissociation (HCD) using normalized collision energy (NCE) of 27, the dynamic exclusion was set to 20 s. Fragment ions were measured in the Orbitrap mass-analyzer with resolving power of 15,000 at m/z 200. Maximum injection time during MS/MS was 100 ms with AGC value of 1.0e5.

Search database

Plasma proteome database [1] was downloaded in XML format from the official website – http://www.plasmaproteomedatabase.org/ (access date: 09.12.2016). Uniprot accession number, information about the experimental evidence, and reported plasma concentration were parsed from the XML files. Only database entries having more than one experimental evidence and at least one indicating that the protein was detected in plasma with valid Uniprot accession number were preserved. The most recent version of protein sequences was obtained from Uniprot via programming interface using accession numbers. If the plasma proteome database entry had several accession numbers assigned, each of them was added individually. Duplicate protein sequences were discarded. The number of proteins in the database after refinement was 3776. The sequences of common contaminants (226 proteins), as used in MaxQuant [2], were added to the database. Finally, the reversed decoy database was concatenated and saved in common FASTA format. All data manipulations were performed using Python (3.6.3) script.

Data analysis

Mass spectrometry data was converted to mzML format using msconvert from ProteoWizard (3.0.9248) [3] and searched with MSGF+ (2016.12.12) [4] against protein database described earlier. Carbamidomethylation of cysteine was used as fixed modification, variable modifications included methionine oxidation, acetylation of protein N-terminus, and carbamylation of peptide N-terminus and lysine. Parent mass tolerance was set to 10 ppm and instrument was set to Q-Exactive. Identifications of all samples in the same experiment (i.e., pH and temperature perturbation) were merged and validated by Percolator (3.01) [5], protein inference was performed by picked protein algorithm, protein FDR was restricted to 0.01. Feature detection, alignment between LC-MS runs and peptide quantification was performed by corresponding tools from OpenMS (2.1.0) [6]. Protein abundance was calculated as a median abundance of three most abundant peptides (Top3). Proteins having less than 3 quantified peptides were excluded. Protein abundancies for each replicate were corrected by subtracting abundancies of the same protein found in the corresponding particle-free control sample. Integration of all tools was programmed in Python (3.6.3).

Differentially abundant proteins

The $\log_{10}$-transformed abundancies of proteins detected in all tested conditions (y) and temperature or pH values (x) were scaled to [0, 1] interval. The relationship was modeled by the sigmoid curve

$$y = \frac{1}{1 + e^{-k(x-x_0)}}.$$

Parameters k and x_0 were selected using non-linear least squares optimization (**curve_fit** from **scipy** module). The fraction of explained variability was required to be higher than 0.5 for the successful fit.

$$1 - \frac{\sum(y'-y)^2}{\sum(y-\bar{y})^2} > 0.5, \text{ where } y' \text{ – predicted value and } \bar{y} \text{ – is the average value}$$

Reversed scaling transformation was applied for the optimized x_0 to obtain critical condition; the sign of k indicated the direction of change.

Minimal spanning tree

The values for amino acid indices for 544 protein parameters presenting in the Kyoto database [7] (v. 9.1) were downloaded from the official website (http://www.genome.jp/aaindex/). Properties annotation as **composition**, **physicochemical property**, **beta propensity**, **other property**, **alpha and turn propensity**, and **hydrophobicity** were extracted from Tomii et al. [8]. Since the paper used an earlier version of Kyoto database only 402 properties got annotations, the others were annotated as **undefined**. Cytoscape (3.6.0) was used to perform network analysis. All pairwise distances between protein properties were calculated as $1 - |R|$, where R is Pearson's correlation coefficient between amino acid indices and used to build minimal spanning tree by Kruskal's algorithm using cySpanningTree (1.1) plugin for Cytoscape. Interactive visualization of the complete spanning tree can be accessed at https://caetera.github.io/AgNPCorona

Lessening analysis

Amino acid indices from Kyoto database were used to calculate the numerical value of each property (544 in total) for all proteins in the protein database (created earlier). The distributions of each individual property for persistent proteins i.e. quantified in all perturbation conditions (189 in temperature experiment, and 173 in pH experiment) and for all proteins in the database were compared. The dispersion of the distribution was calculated as the difference between 10th and 90th percentiles and used to calculate the change in dispersion (lessening).

$$Lessening = \frac{{}^{90}_{Pers}C - {}^{10}_{Pers}C}{{}^{90}_{All}C - {}^{10}_{All}C},$$

$${}^{90}_{Pers}C \text{ and } {}^{10}_{Pers}C - 90^{th} \text{ and } 10^{th} \text{ percentiles of property } C \text{ for persistent proteins,}$$

$${}^{90}_{All}C \text{ and } {}^{10}_{All}C - 90^{th} \text{ and } 10^{th} \text{ percentiles of property } C \text{ for all proteins}$$

Significance was estimated by a permutation test. The test was performed in the following way. The distribution of the lessening under null-hypothesis was estimated by sampling (100000 times) a subset of proteins without repetition from the complete population. The size of this subset was the same as the size of the persistent group in the corresponding experiment (i.e. 189 or 173). The lessening was calculated using this subset as the persistent group. The resulting distribution was fitted by the normal one. The parameters were calculated using non-linear least squares optimization (**curve_fit** from **scipy** module). The p-value was

calculated as cumulative distribution function (two-tailed) of the fitted normal distribution. Resulting p-values were corrected using Benjamini-Hochberg method.

Protein properties in **alpha and turn propensities** and **beta propensity** groups displaying significant change in the distribution for persistent fraction were manually assigned a coefficient (1, or –1) indicating, if the increase in numerical value indicates the increase in the corresponding property, for example, an increase of beta-sheet content in the protein (coefficient 1) or vice versa (coefficient –1). The coefficients for properties in hydrophobicity group were calculated as the sign of the difference between the numeric value for isoleucine (hydrophobic) and aspartic acid (hydrophilic). The coefficients were used to calculate the direction of change (expressed as the difference between median values) in the persistent protein fraction relative to the background. Alpha and turn propensities were split into two separate groups.

All used scripts are published on GitHub: https://github.com/caetera/AgNPCorona

Supplementary Figures

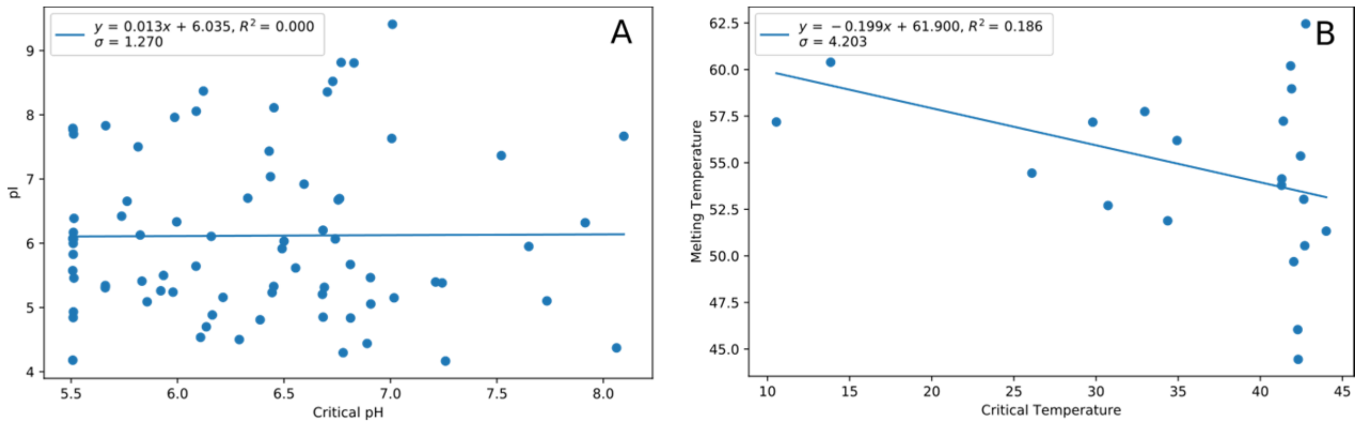

Figure S1. Correlation of critical pH and isoelectric point (A) and critical temperature with the melting temperature (B) for differentially abundant protein

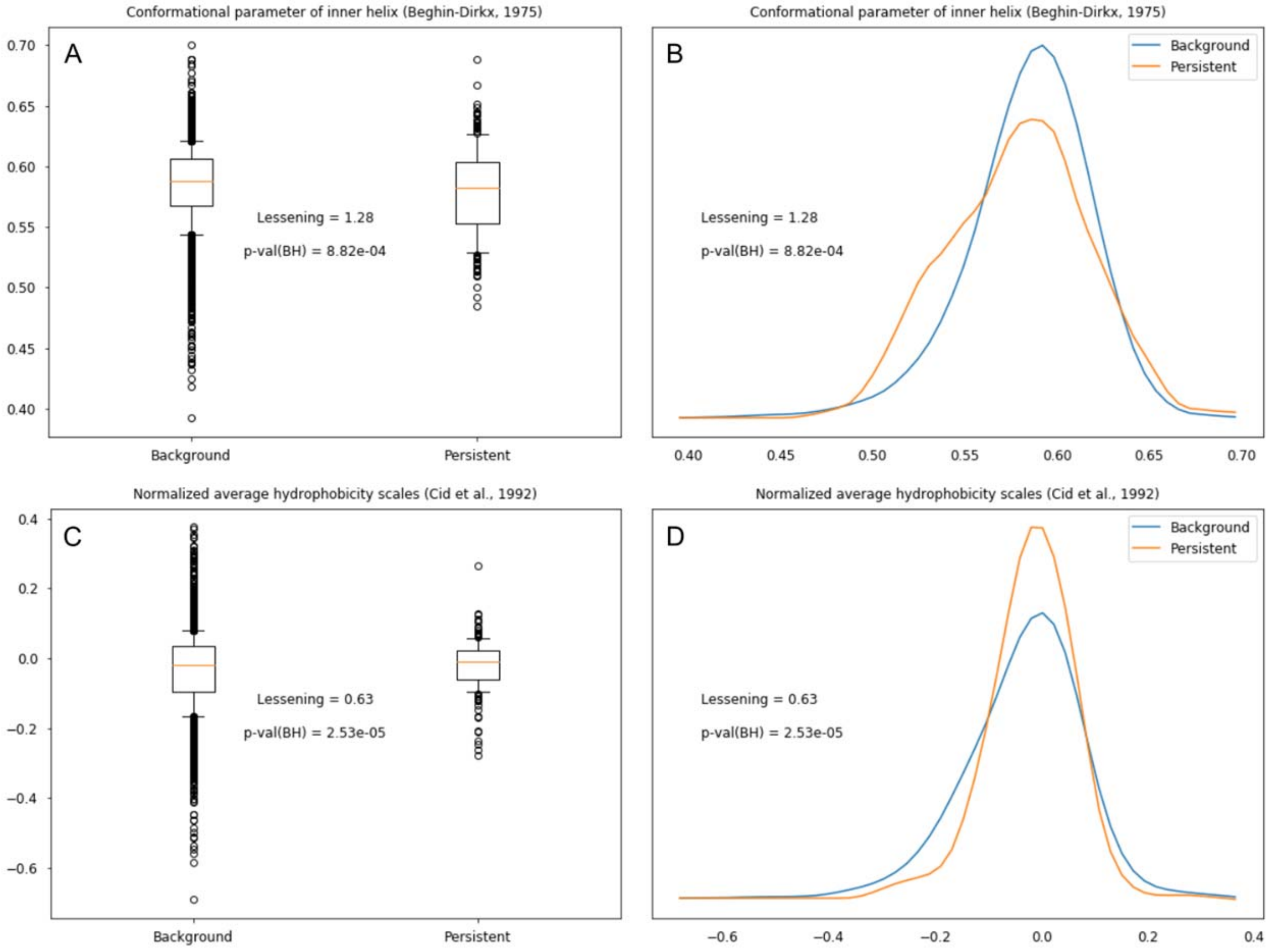

Figure S2. Example of dispersion change (lessening) analysis. (A) The persistent proteins have wider spread, than background – lessening > 1; (C) distribution of numerical values of the corresponding protein property, additional second component can be observed; (B) the persistent proteins have narrower spread, than background – lessening < 1; (D) value distribution for persistent proteins is narrower and has one

component. The significance is estimated by permutation test and corrected according to Benjamini-Hochberg. The whiskers on boxplots show the 10th and 90th percentile of the distribution.

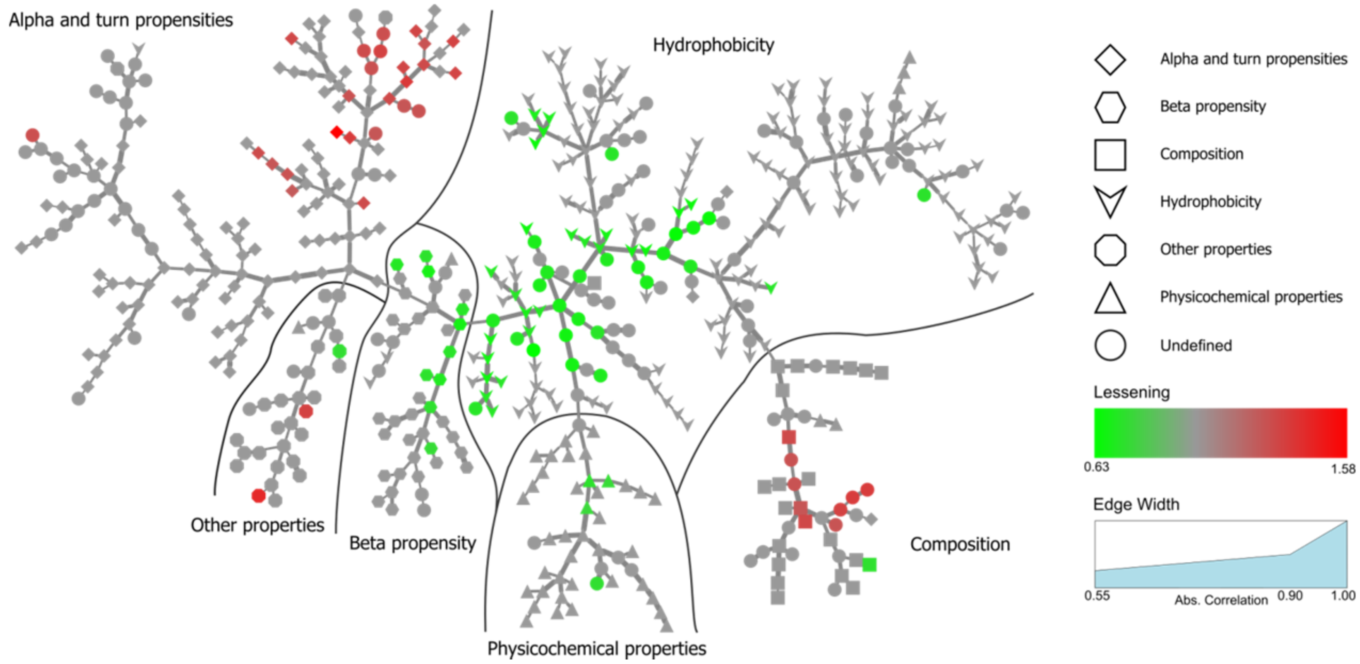

Figure S3. Minimum spanning trees for the pH perturbation experiment. Properties are divided into six main categories and individual properties are color coded according to the degree of lessening in spread between persistent proteins and proteins found in plasma. Only nodes corresponding to significant changes (FDR < 0.005) are colored.

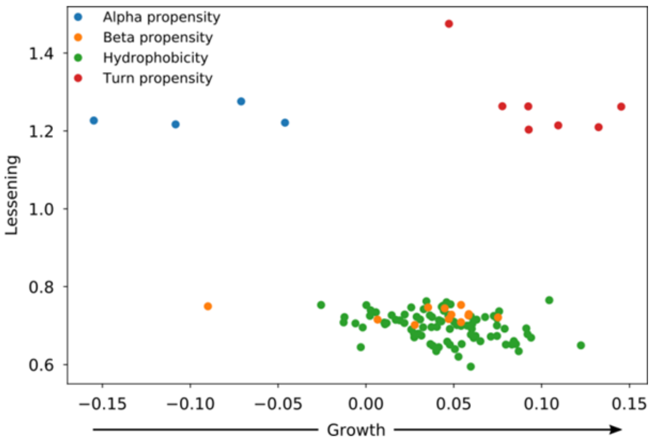

Figure S4. The direction of change for protein properties displaying significant lessening or broadening of distribution in persistent protein fraction.

Supplementary Tables

Table S1. Number of proteins in each protein classes

Experiment	Class I	Class II	Class III
Temperature	123	32	34
pH	96	59	18

References

1. Nanjappa, V., et al., *Plasma Proteome Database as a resource for proteomics research: 2014 update.* Nucleic Acids Res, 2014. **42**(Database issue): p. D959-65.
2. Cox, J. and M. Mann, *MaxQuant enables high peptide identification rates, individualized p.p.b.-range mass accuracies and proteome-wide protein quantification.* Nat Biotechnol, 2008. **26**(12): p. 1367-72.
3. Chambers, M.C., et al., *A cross-platform toolkit for mass spectrometry and proteomics.* Nat Biotechnol, 2012. **30**(10): p. 918-20.
4. Kim, S. and P.A. Pevzner, *MS-GF+ makes progress towards a universal database search tool for proteomics.* Nat Commun, 2014. **5**: p. 5277.
5. The, M., et al., *Fast and Accurate Protein False Discovery Rates on Large-Scale Proteomics Data Sets with Percolator 3.0.* J Am Soc Mass Spectrom, 2016. **27**(11): p. 1719-1727.
6. Rost, H.L., et al., *OpenMS: a flexible open-source software platform for mass spectrometry data analysis.* Nat Methods, 2016. **13**(9): p. 741-8.
7. Kawashima, S., et al., *AAindex: amino acid index database, progress report 2008.* Nucleic Acids Res, 2008. **36**(Database issue): p. D202-5.
8. Tomii, K. and M. Kanehisa, *Analysis of amino acid indices and mutation matrices for sequence comparison and structure prediction of proteins.* Protein Eng, 1996. **9**(1): p. 27-36.

Differentially abundant proteins in pH experiment

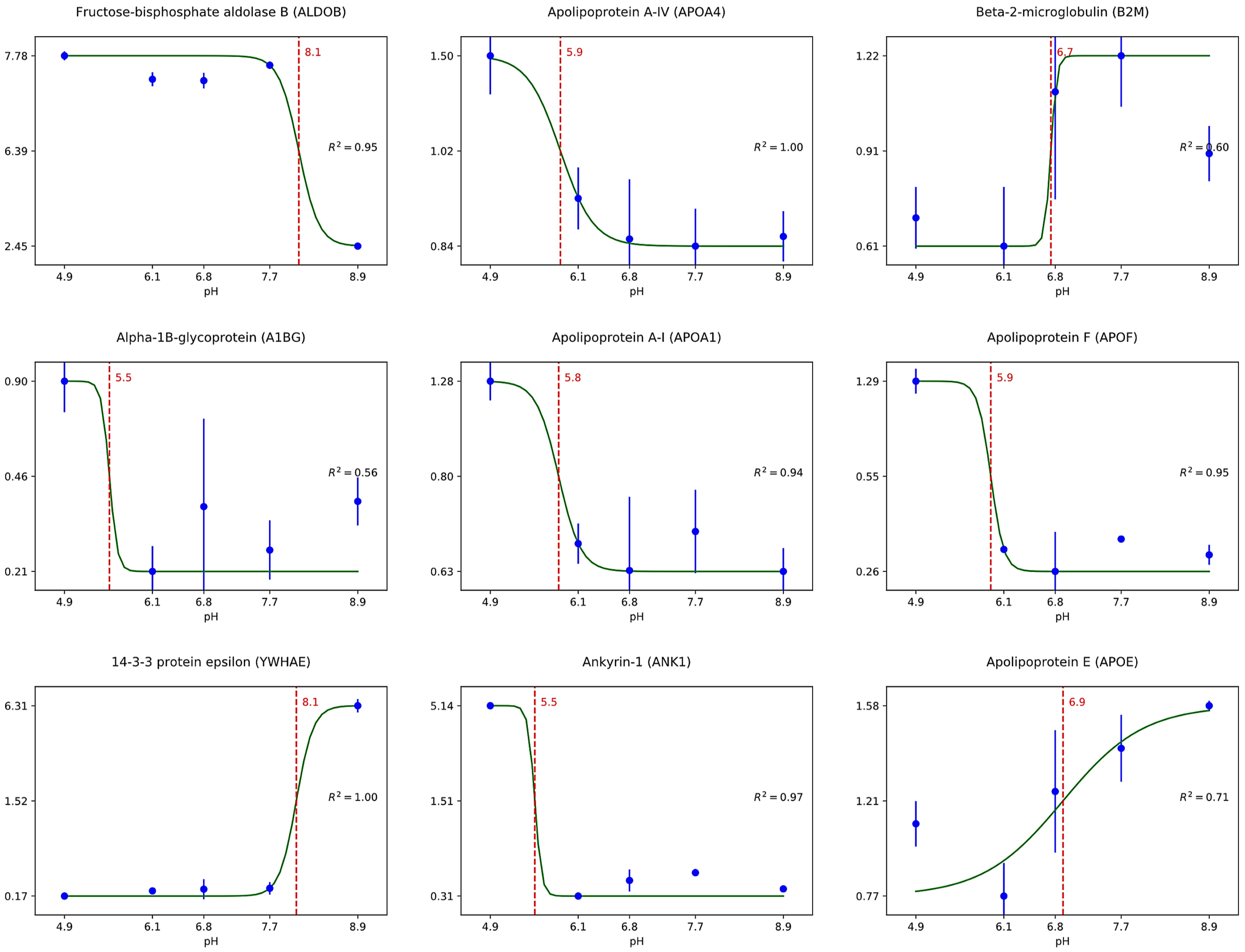

Fructose-bisphosphate aldolase B (ALDOB)
7.78
6.39
2.45
8.1
$R^2 = 0.95$
4.9
6.1
6.8
7.7
8.9
pH
Apolipoprotein A-IV (APOA4)
1.50
1.02
0.84
5.9
$R^2 = 1.00$
4.9
6.1
6.8
7.7
8.9
pH
Beta-2-microglobulin (B2M)
1.22
0.91
0.61
6.7
$R^2 = 0.60$
4.9
6.1
6.8
7.7
8.9
pH
Alpha-1B-glycoprotein (A1BG)
0.90
0.46
0.21
5.5
$R^2 = 0.56$
4.9
6.1
6.8
7.7
8.9
pH
Apolipoprotein A-I (APOA1)
1.28
0.80
0.63
5.8
$R^2 = 0.94$
4.9
6.1
6.8
7.7
8.9
pH
Apolipoprotein F (APOF)
1.29
0.55
0.26
5.9
$R^2 = 0.95$
4.9
6.1
6.8
7.7
8.9
pH
14-3-3 protein epsilon (YWHAE)
6.31
1.52
0.17
8.1
$R^2 = 1.00$
4.9
6.1
6.8
7.7
8.9
pH
Ankyrin-1 (ANK1)
5.14
1.51
0.31
5.5
$R^2 = 0.97$
4.9
6.1
6.8
7.7
8.9
pH
Apolipoprotein E (APOE)
1.58
1.21
0.77
6.9
$R^2 = 0.71$
4.9
6.1
6.8
7.7
8.9
pH

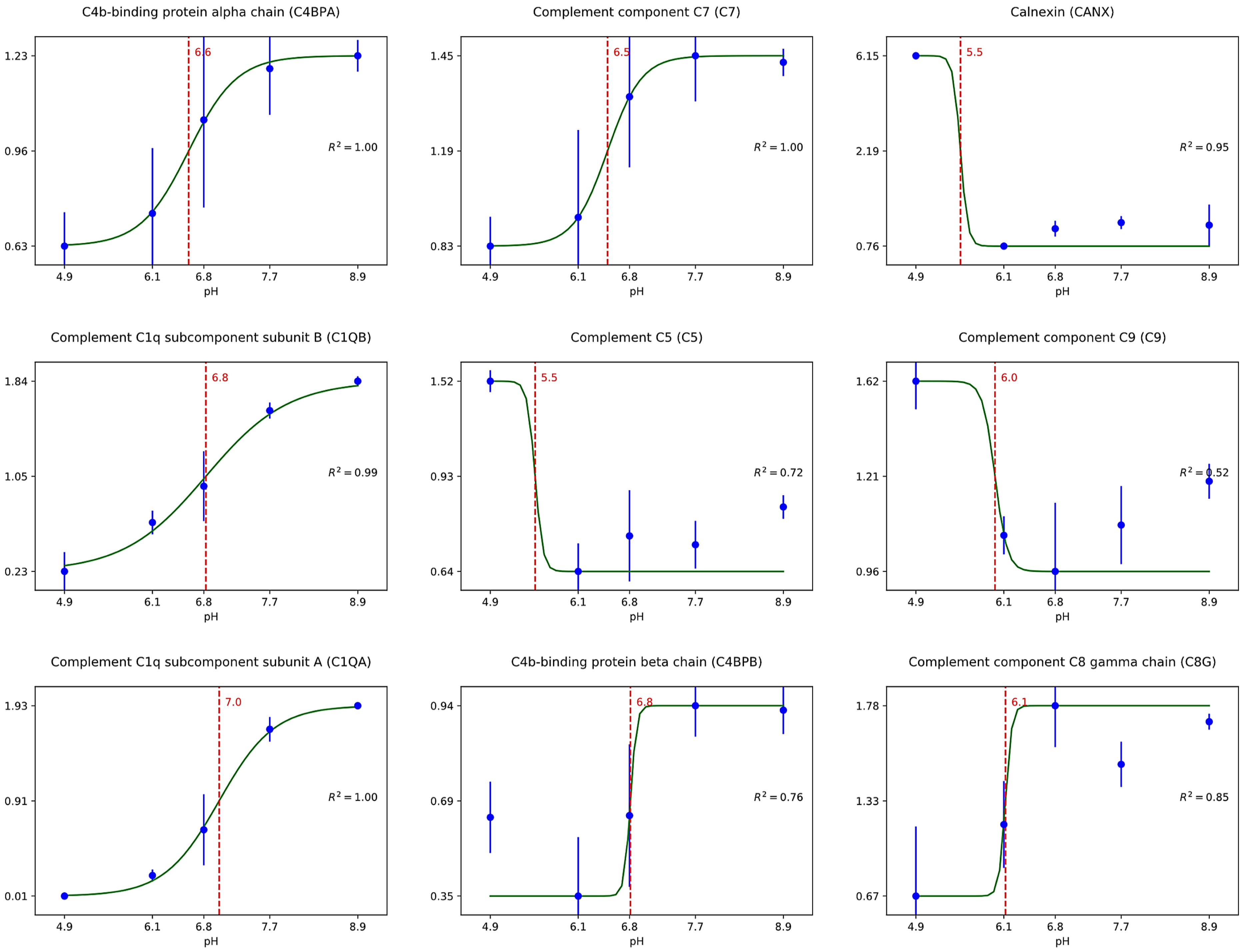
C4b-binding protein alpha chain (C4BPA)
1.23
0.96
0.63
6.6
$R^2 = 1.00$
4.9 6.1 6.8 7.7 8.9
pH
Complement component C7 (C7)
1.45
1.19
0.83
6.5
$R^2 = 1.00$
4.9 6.1 6.8 7.7 8.9
pH
Calnexin (CANX)
6.15
2.19
0.76
5.5
$R^2 = 0.95$
4.9 6.1 6.8 7.7 8.9
pH
Complement C1q subcomponent subunit B (C1QB)
1.84
1.05
0.23
6.8
$R^2 = 0.99$
4.9 6.1 6.8 7.7 8.9
pH
Complement C5 (C5)
1.52
0.93
0.64
5.5
$R^2 = 0.72$
4.9 6.1 6.8 7.7 8.9
pH
Complement component C9 (C9)
1.62
1.21
0.96
6.0
$R^2 = 0.52$
4.9 6.1 6.8 7.7 8.9
pH
Complement C1q subcomponent subunit A (C1QA)
1.93
0.91
0.01
7.0
$R^2 = 1.00$
4.9 6.1 6.8 7.7 8.9
pH
C4b-binding protein beta chain (C4BPB)
0.94
0.69
0.35
6.8
$R^2 = 0.76$
4.9 6.1 6.8 7.7 8.9
pH
Complement component C8 gamma chain (C8G)
1.78
1.33
0.67
6.1
$R^2 = 0.85$
4.9 6.1 6.8 7.7 8.9
pH

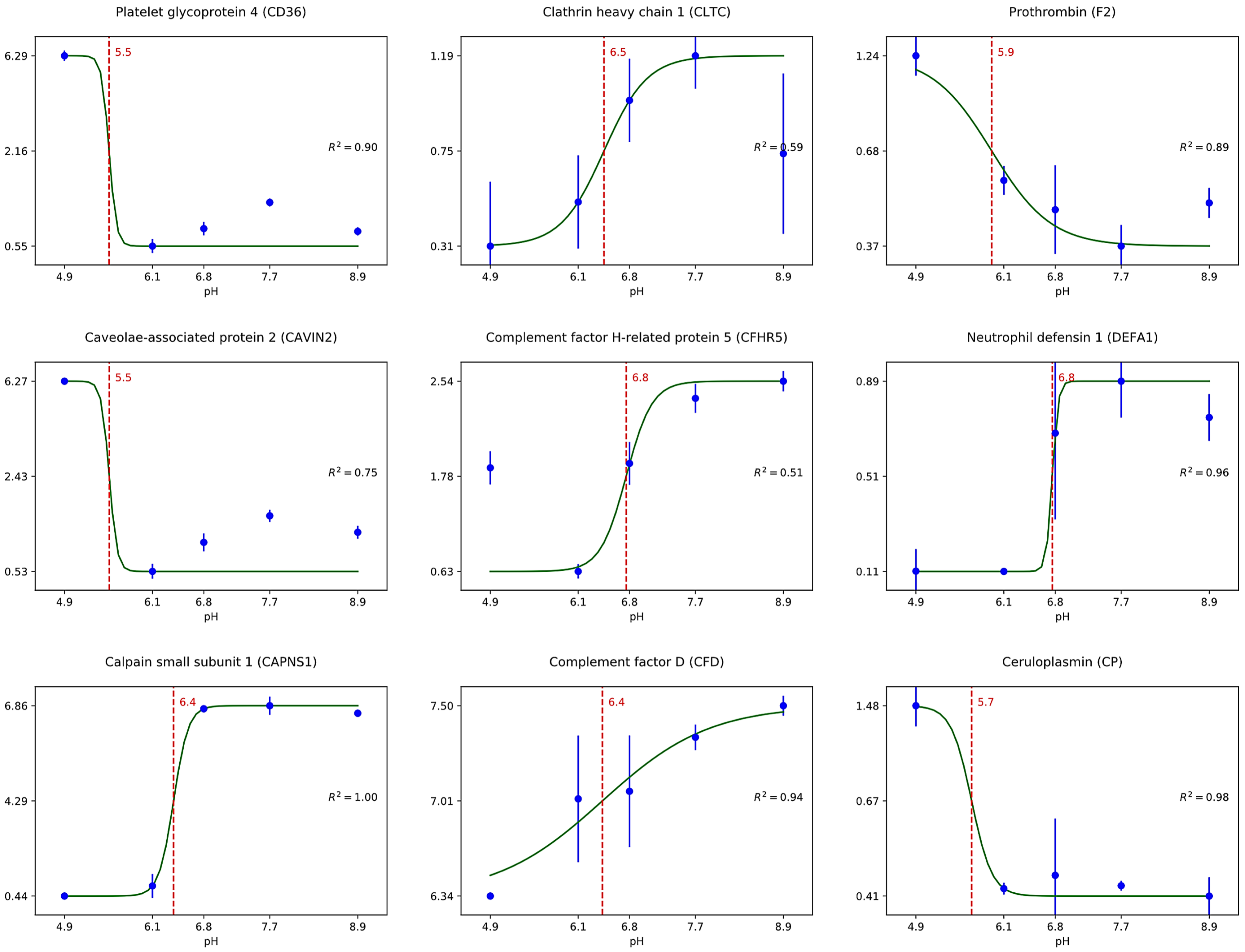
Platelet glycoprotein 4 (CD36)
Clathrin heavy chain 1 (CLTC)
Prothrombin (F2)
Caveolae-associated protein 2 (CAVIN2)
Complement factor H-related protein 5 (CFHR5)
Neutrophil defensin 1 (DEFA1)
Calpain small subunit 1 (CAPNS1)
Complement factor D (CFD)
Ceruloplasmin (CP)
pH
4.9
6.1
6.8
7.7
8.9
$R^2 = 0.90$
$R^2 = 0.59$
$R^2 = 0.89$
$R^2 = 0.75$
$R^2 = 0.51$
$R^2 = 0.96$
$R^2 = 1.00$
$R^2 = 0.94$
$R^2 = 0.98$
5.5
6.5
5.9
5.5
6.8
6.8
6.4
6.4
5.7

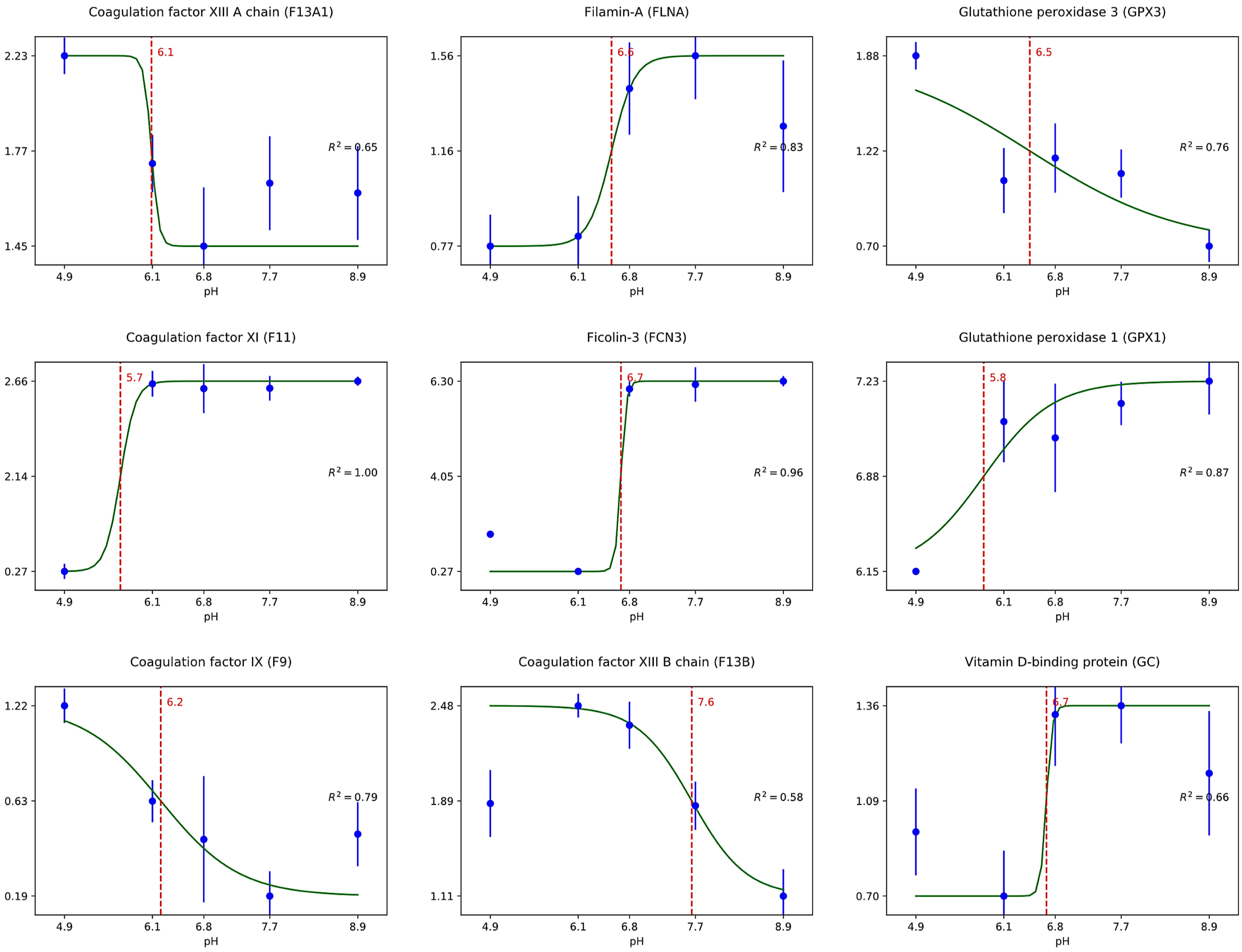

Coagulation factor XIII A chain (F13A1)
2.23
1.77
1.45
6.1
$R^2 = 0.65$
4.9
6.1
6.8
7.7
8.9
pH
Filamin-A (FLNA)
1.56
1.16
0.77
6.6
$R^2 = 0.83$
Glutathione peroxidase 3 (GPX3)
1.88
1.22
0.70
6.5
$R^2 = 0.76$
Coagulation factor XI (F11)
2.66
2.14
0.27
5.7
$R^2 = 1.00$
Ficolin-3 (FCN3)
6.30
4.05
0.27
6.7
$R^2 = 0.96$
Glutathione peroxidase 1 (GPX1)
7.23
6.88
6.15
5.8
$R^2 = 0.87$
Coagulation factor IX (F9)
1.22
0.63
0.19
6.2
$R^2 = 0.79$
Coagulation factor XIII B chain (F13B)
2.48
1.89
1.11
7.6
$R^2 = 0.58$
Vitamin D-binding protein (GC)
1.36
1.09
0.70
6.7
$R^2 = 0.66$

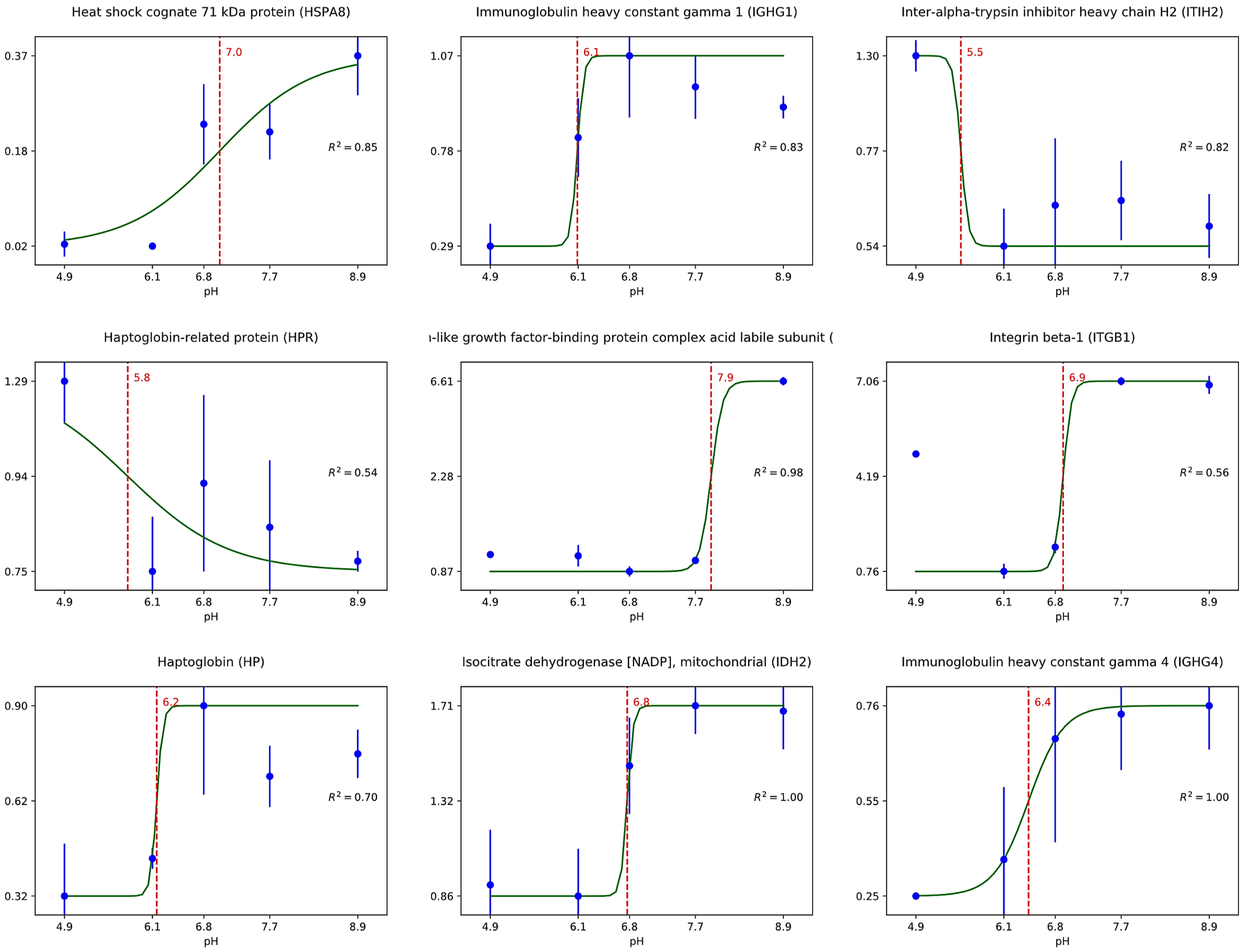
Heat shock cognate 71 kDa protein (HSPA8)
0.37
0.18
0.02
7.0
$R^2 = 0.85$
4.9
6.1
6.8
7.7
8.9
pH
Immunoglobulin heavy constant gamma 1 (IGHG1)
1.07
0.78
0.29
6.1
$R^2 = 0.83$
pH
Inter-alpha-trypsin inhibitor heavy chain H2 (ITIH2)
1.30
0.77
0.54
5.5
$R^2 = 0.82$
pH
Haptoglobin-related protein (HPR)
1.29
0.94
0.75
5.8
$R^2 = 0.54$
pH
ı-like growth factor-binding protein complex acid labile subunit (
6.61
2.28
0.87
7.9
$R^2 = 0.98$
pH
Integrin beta-1 (ITGB1)
7.06
4.19
0.76
6.9
$R^2 = 0.56$
pH
Haptoglobin (HP)
0.90
0.62
0.32
6.2
$R^2 = 0.70$
pH
Isocitrate dehydrogenase [NADP], mitochondrial (IDH2)
1.71
1.32
0.86
6.8
$R^2 = 1.00$
pH
Immunoglobulin heavy constant gamma 4 (IGHG4)
0.76
0.55
0.25
6.4
$R^2 = 1.00$
pH

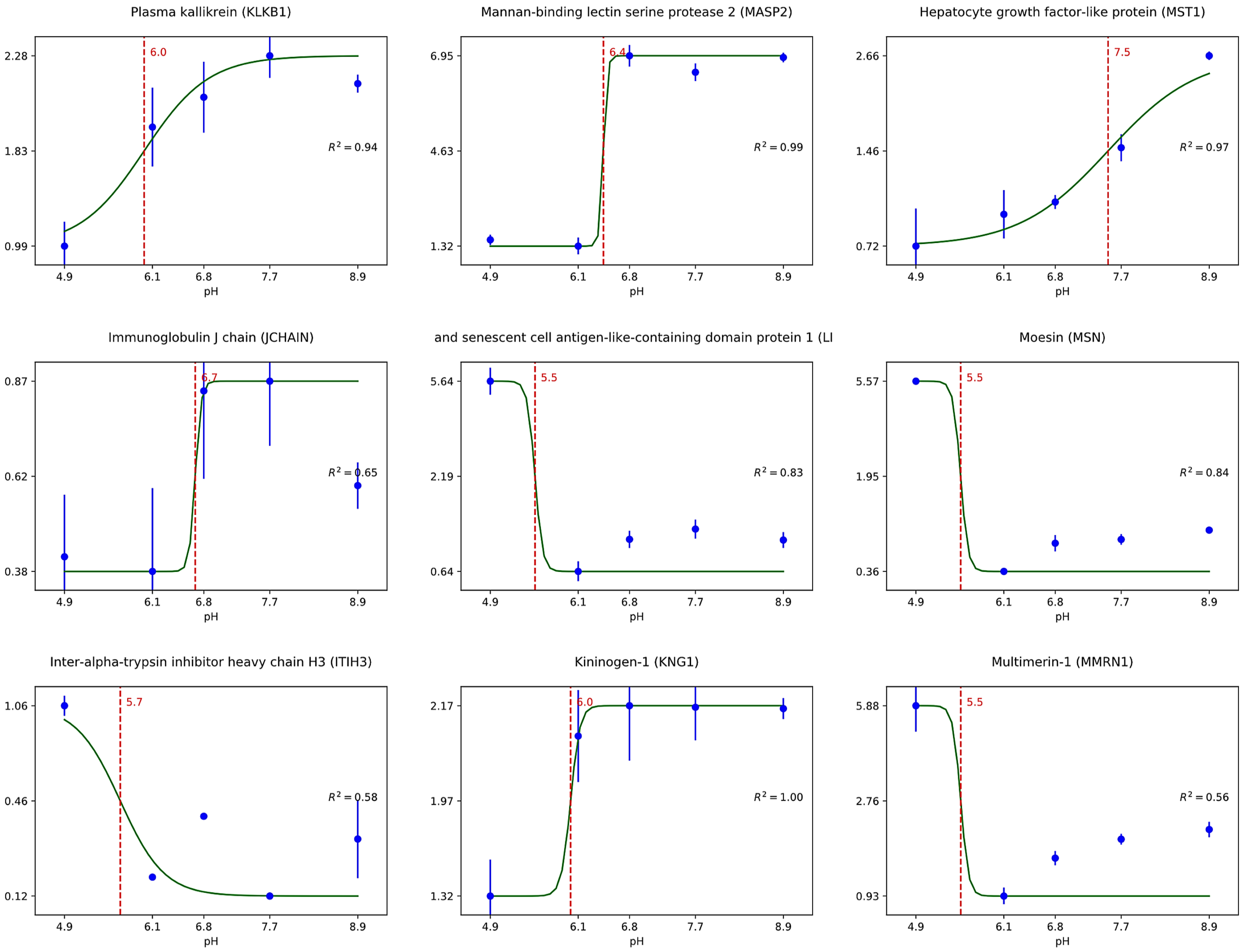
Plasma kallikrein (KLKB1)
Mannan-binding lectin serine protease 2 (MASP2)
Hepatocyte growth factor-like protein (MST1)
Immunoglobulin J chain (JCHAIN)
and senescent cell antigen-like-containing domain protein 1 (LI
Moesin (MSN)
Inter-alpha-trypsin inhibitor heavy chain H3 (ITIH3)
Kininogen-1 (KNG1)
Multimerin-1 (MMRN1)
pH
4.9
6.1
6.8
7.7
8.9
$R^2 = 0.94$
$R^2 = 0.99$
$R^2 = 0.97$
$R^2 = 0.65$
$R^2 = 0.83$
$R^2 = 0.84$
$R^2 = 0.58$
$R^2 = 1.00$
$R^2 = 0.56$
6.0
6.4
7.5
6.7
5.5
5.5
5.7
6.0
5.5
2.28
1.83
0.99
6.95
4.63
1.32
2.66
1.46
0.72
0.87
0.62
0.38
5.64
2.19
0.64
5.57
1.95
0.36
1.06
0.46
0.12
2.17
1.97
1.32
5.88
2.76
0.93

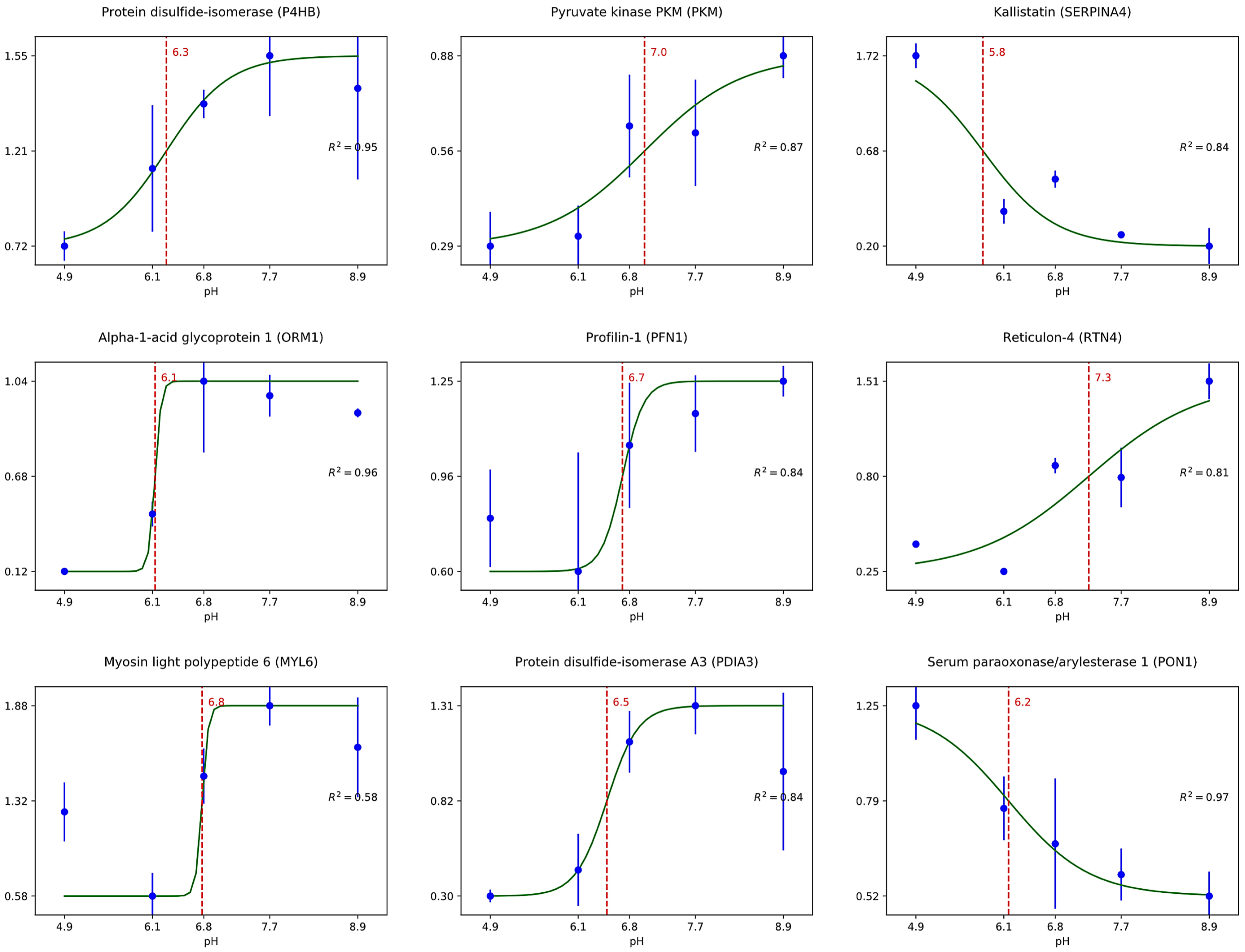

Protein disulfide-isomerase (P4HB)
6.3
$R^2 = 0.95$
Pyruvate kinase PKM (PKM)
7.0
$R^2 = 0.87$
Kallistatin (SERPINA4)
5.8
$R^2 = 0.84$
Alpha-1-acid glycoprotein 1 (ORM1)
6.1
$R^2 = 0.96$
Profilin-1 (PFN1)
6.7
$R^2 = 0.84$
Reticulon-4 (RTN4)
7.3
$R^2 = 0.81$
Myosin light polypeptide 6 (MYL6)
6.8
$R^2 = 0.58$
Protein disulfide-isomerase A3 (PDIA3)
6.5
$R^2 = 0.84$
Serum paraoxonase/arylesterase 1 (PON1)
6.2
$R^2 = 0.97$
pH
4.9
6.1
6.8
7.7
8.9

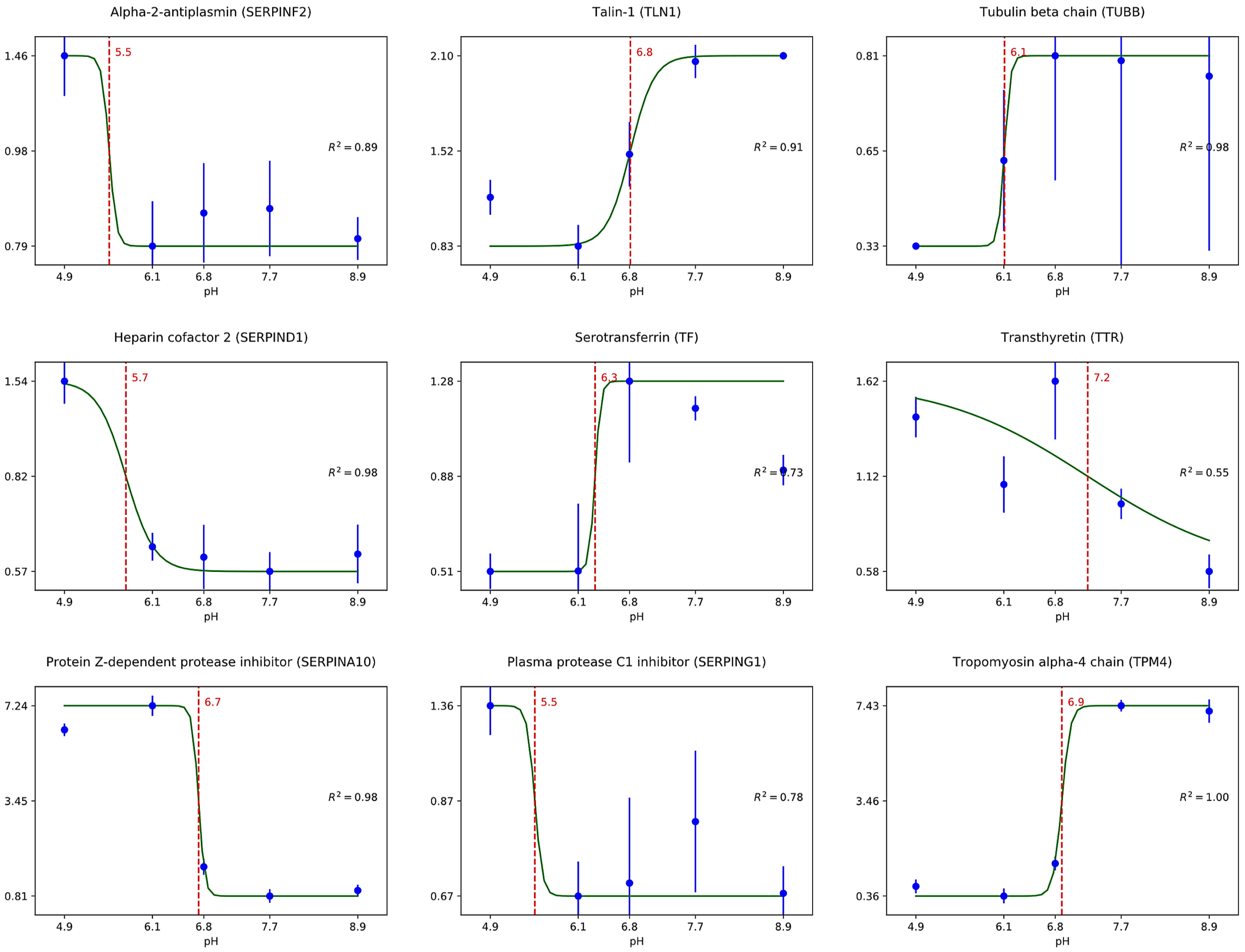

Alpha-2-antiplasmin (SERPINF2)
Talin-1 (TLN1)
Tubulin beta chain (TUBB)
Heparin cofactor 2 (SERPIND1)
Serotransferrin (TF)
Transthyretin (TTR)
Protein Z-dependent protease inhibitor (SERPINA10)
Plasma protease C1 inhibitor (SERPING1)
Tropomyosin alpha-4 chain (TPM4)
pH
$R^2 = 0.89$
$R^2 = 0.91$
$R^2 = 0.98$
$R^2 = 0.98$
$R^2 = 0.73$
$R^2 = 0.55$
$R^2 = 0.98$
$R^2 = 0.78$
$R^2 = 1.00$

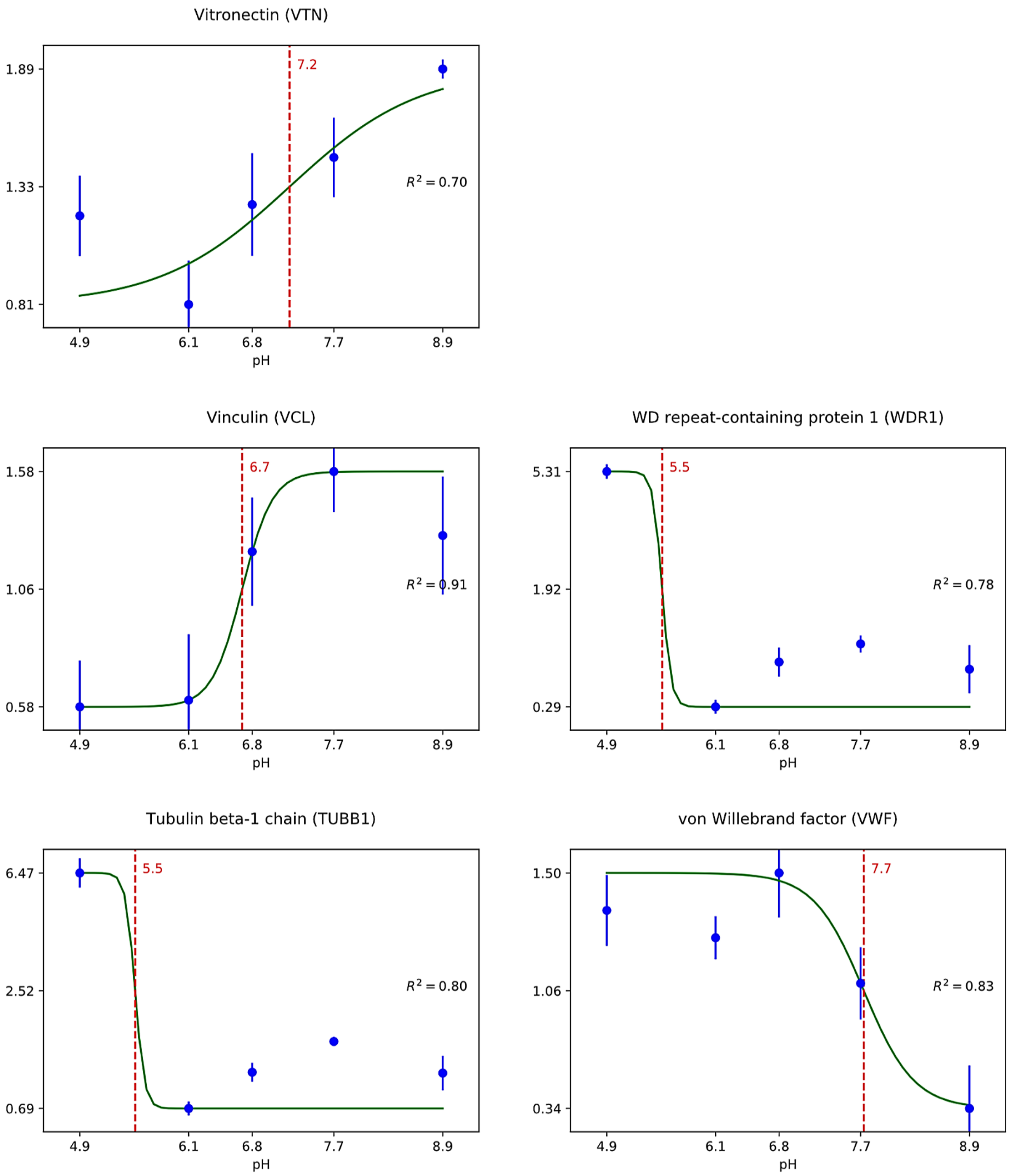
Vitronectin (VTN)
1.89
1.33
0.81
7.2
$R^2 = 0.70$
4.9
6.1
6.8
7.7
8.9
pH
Vinculin (VCL)
1.58
1.06
0.58
6.7
$R^2 = 0.91$
4.9
6.1
6.8
7.7
8.9
pH
WD repeat-containing protein 1 (WDR1)
5.31
1.92
0.29
5.5
$R^2 = 0.78$
4.9
6.1
6.8
7.7
8.9
pH
Tubulin beta-1 chain (TUBB1)
6.47
2.52
0.69
5.5
$R^2 = 0.80$
4.9
6.1
6.8
7.7
8.9
pH
von Willebrand factor (VWF)
1.50
1.06
0.34
7.7
$R^2 = 0.83$
4.9
6.1
6.8
7.7
8.9
pH

Differentially abundant proteins in temperature experiment

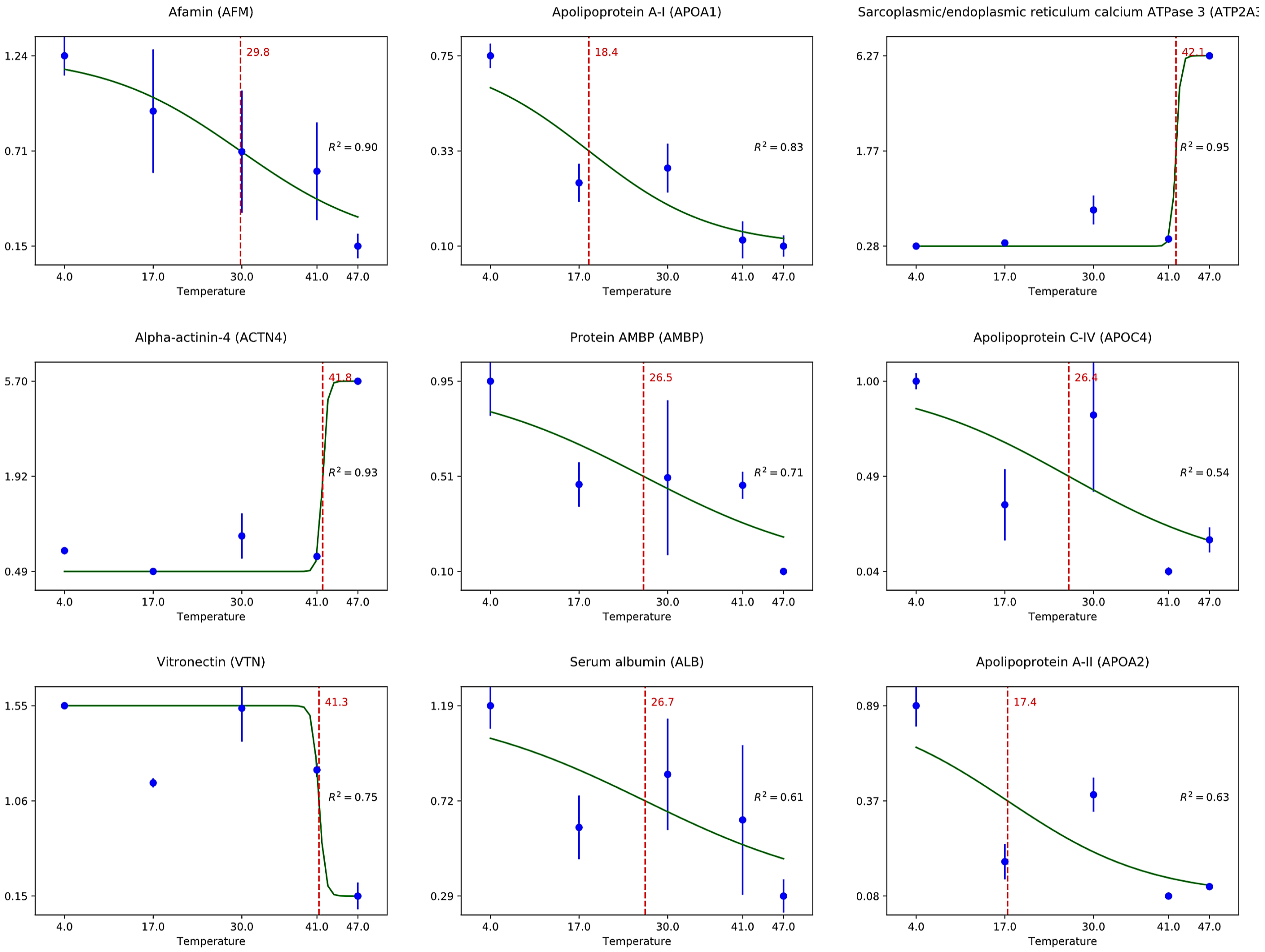
Afamin (AFM)
Apolipoprotein A-I (APOA1)
Sarcoplasmic/endoplasmic reticulum calcium ATPase 3 (ATP2A3
Alpha-actinin-4 (ACTN4)
Protein AMBP (AMBP)
Apolipoprotein C-IV (APOC4)
Vitronectin (VTN)
Serum albumin (ALB)
Apolipoprotein A-II (APOA2)
Temperature
29.8
18.4
42.1
41.8
26.5
26.4
41.3
26.7
17.4
$R^2 = 0.90$
$R^2 = 0.83$
$R^2 = 0.95$
$R^2 = 0.93$
$R^2 = 0.71$
$R^2 = 0.54$
$R^2 = 0.75$
$R^2 = 0.61$
$R^2 = 0.63$

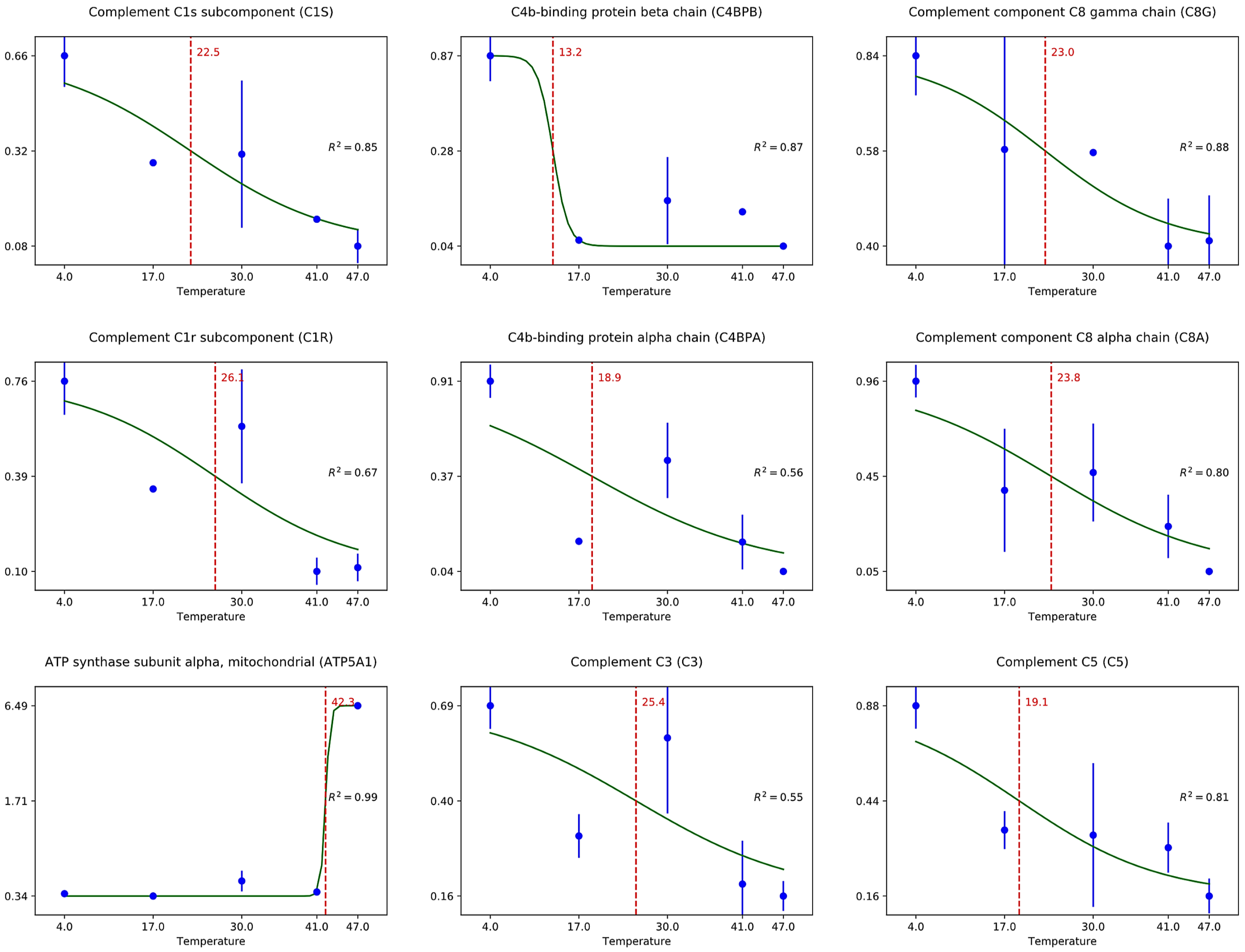
Complement C1s subcomponent (C1S)
0.66
0.32
0.08
22.5
$R^2 = 0.85$
4.0
17.0
30.0
41.0
47.0
Temperature
C4b-binding protein beta chain (C4BPB)
0.87
0.28
0.04
13.2
$R^2 = 0.87$
4.0
17.0
30.0
41.0
47.0
Temperature
Complement component C8 gamma chain (C8G)
0.84
0.58
0.40
23.0
$R^2 = 0.88$
4.0
17.0
30.0
41.0
47.0
Temperature
Complement C1r subcomponent (C1R)
0.76
0.39
0.10
26.1
$R^2 = 0.67$
4.0
17.0
30.0
41.0
47.0
Temperature
C4b-binding protein alpha chain (C4BPA)
0.91
0.37
0.04
18.9
$R^2 = 0.56$
4.0
17.0
30.0
41.0
47.0
Temperature
Complement component C8 alpha chain (C8A)
0.96
0.45
0.05
23.8
$R^2 = 0.80$
4.0
17.0
30.0
41.0
47.0
Temperature
ATP synthase subunit alpha, mitochondrial (ATP5A1)
6.49
1.71
0.34
42.3
$R^2 = 0.99$
4.0
17.0
30.0
41.0
47.0
Temperature
Complement C3 (C3)
0.69
0.40
0.16
25.4
$R^2 = 0.55$
4.0
17.0
30.0
41.0
47.0
Temperature
Complement C5 (C5)
0.88
0.44
0.16
19.1
$R^2 = 0.81$
4.0
17.0
30.0
41.0
47.0
Temperature

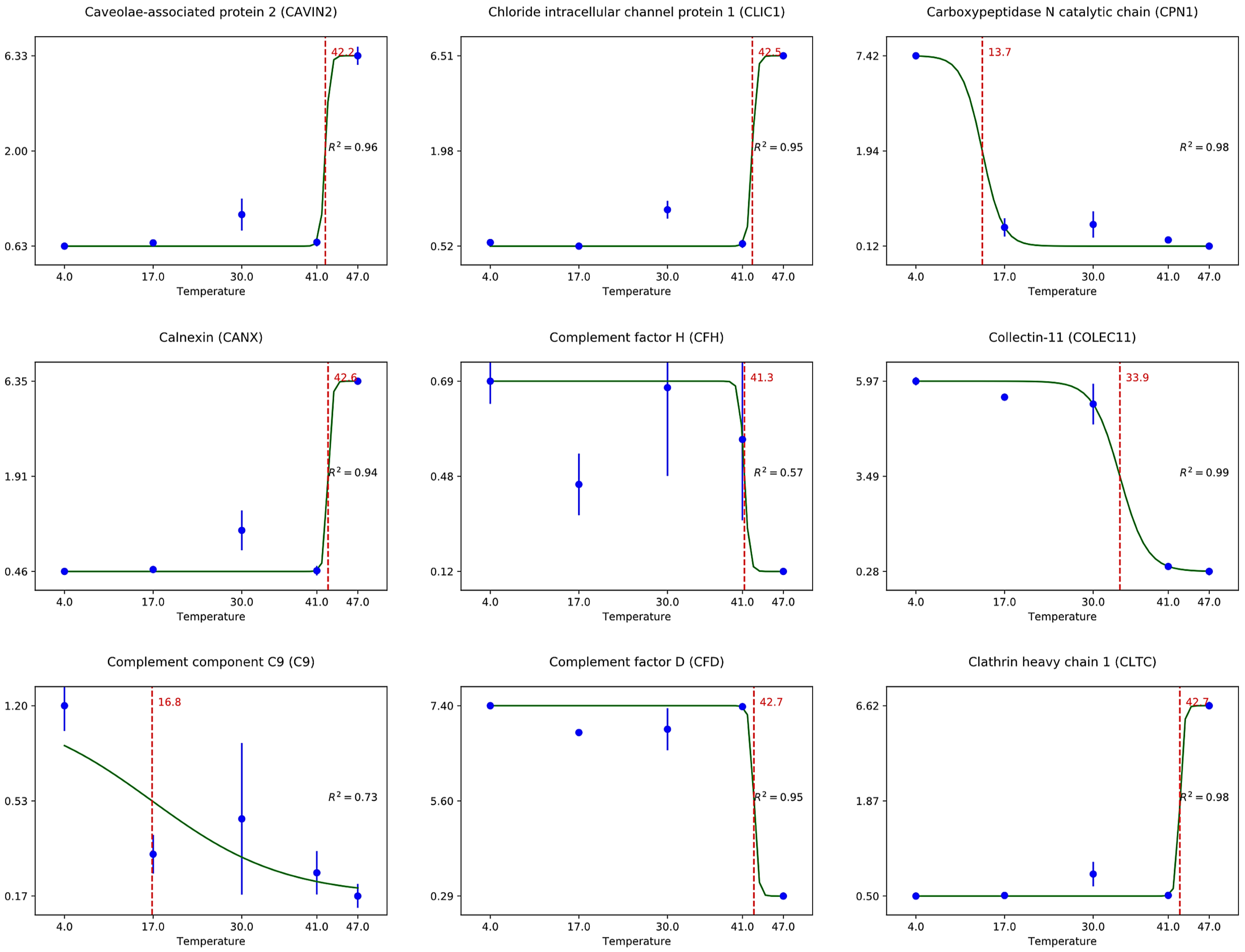

Caveolae-associated protein 2 (CAVIN2)
Chloride intracellular channel protein 1 (CLIC1)
Carboxypeptidase N catalytic chain (CPN1)
Calnexin (CANX)
Complement factor H (CFH)
Collectin-11 (COLEC11)
Complement component C9 (C9)
Complement factor D (CFD)
Clathrin heavy chain 1 (CLTC)
Temperature
$R^2 = 0.96$
$R^2 = 0.95$
$R^2 = 0.98$
$R^2 = 0.94$
$R^2 = 0.57$
$R^2 = 0.99$
$R^2 = 0.73$
$R^2 = 0.95$
$R^2 = 0.98$
42.2
42.5
13.7
42.6
41.3
33.9
16.8
42.7
42.7

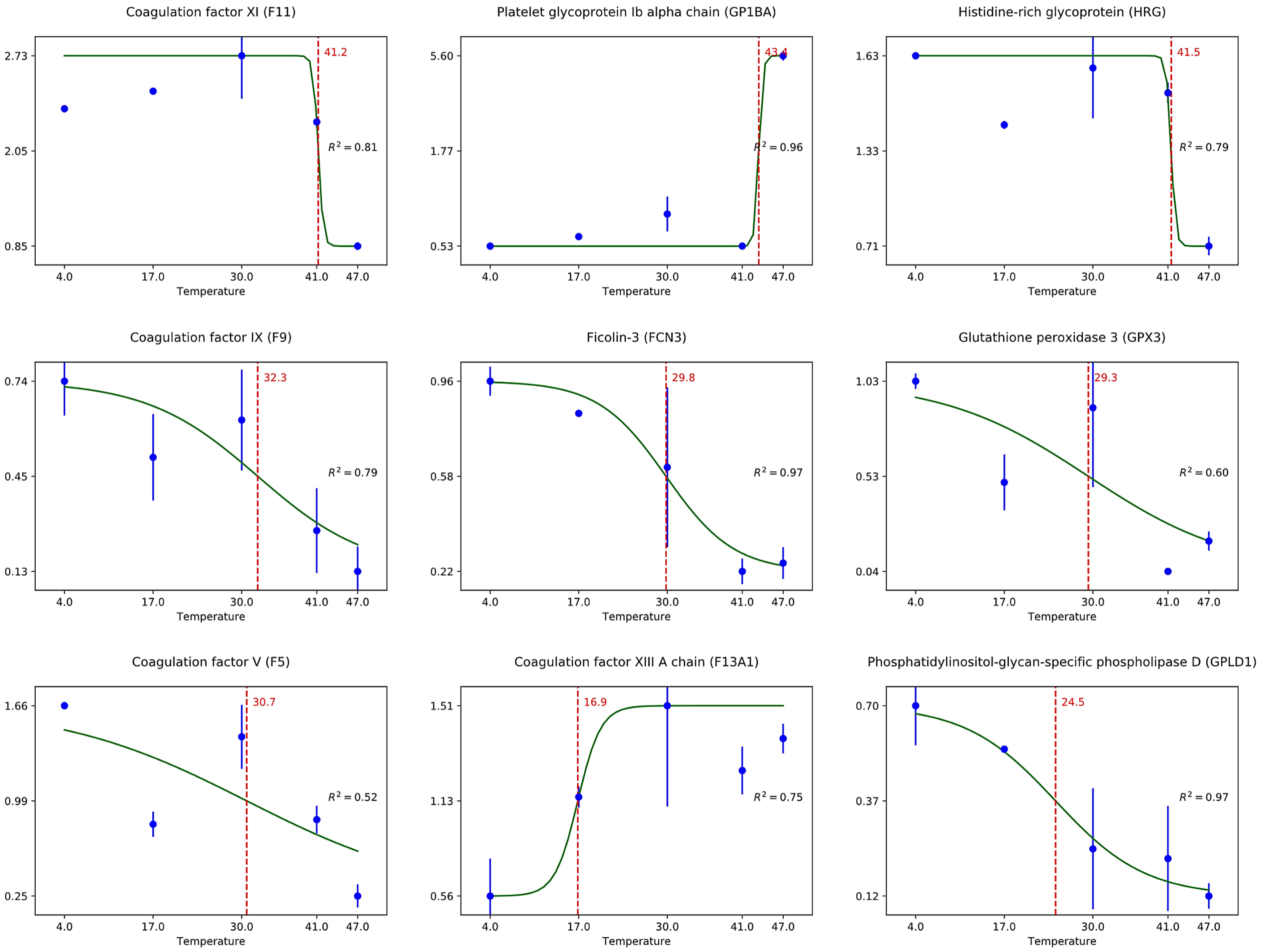

Coagulation factor XI (F11)
2.73
2.05
0.85
41.2
$R^2 = 0.81$
4.0
17.0
30.0
41.0
47.0
Temperature
Platelet glycoprotein Ib alpha chain (GP1BA)
5.60
1.77
0.53
43.4
$R^2 = 0.96$
4.0
17.0
30.0
41.0
47.0
Temperature
Histidine-rich glycoprotein (HRG)
1.63
1.33
0.71
41.5
$R^2 = 0.79$
4.0
17.0
30.0
41.0
47.0
Temperature
Coagulation factor IX (F9)
0.74
0.45
0.13
32.3
$R^2 = 0.79$
4.0
17.0
30.0
41.0
47.0
Temperature
Ficolin-3 (FCN3)
0.96
0.58
0.22
29.8
$R^2 = 0.97$
4.0
17.0
30.0
41.0
47.0
Temperature
Glutathione peroxidase 3 (GPX3)
1.03
0.53
0.04
29.3
$R^2 = 0.60$
4.0
17.0
30.0
41.0
47.0
Temperature
Coagulation factor V (F5)
1.66
0.99
0.25
30.7
$R^2 = 0.52$
4.0
17.0
30.0
41.0
47.0
Temperature
Coagulation factor XIII A chain (F13A1)
1.51
1.13
0.56
16.9
$R^2 = 0.75$
4.0
17.0
30.0
41.0
47.0
Temperature
Phosphatidylinositol-glycan-specific phospholipase D (GPLD1)
0.70
0.37
0.12
24.5
$R^2 = 0.97$
4.0
17.0
30.0
41.0
47.0
Temperature

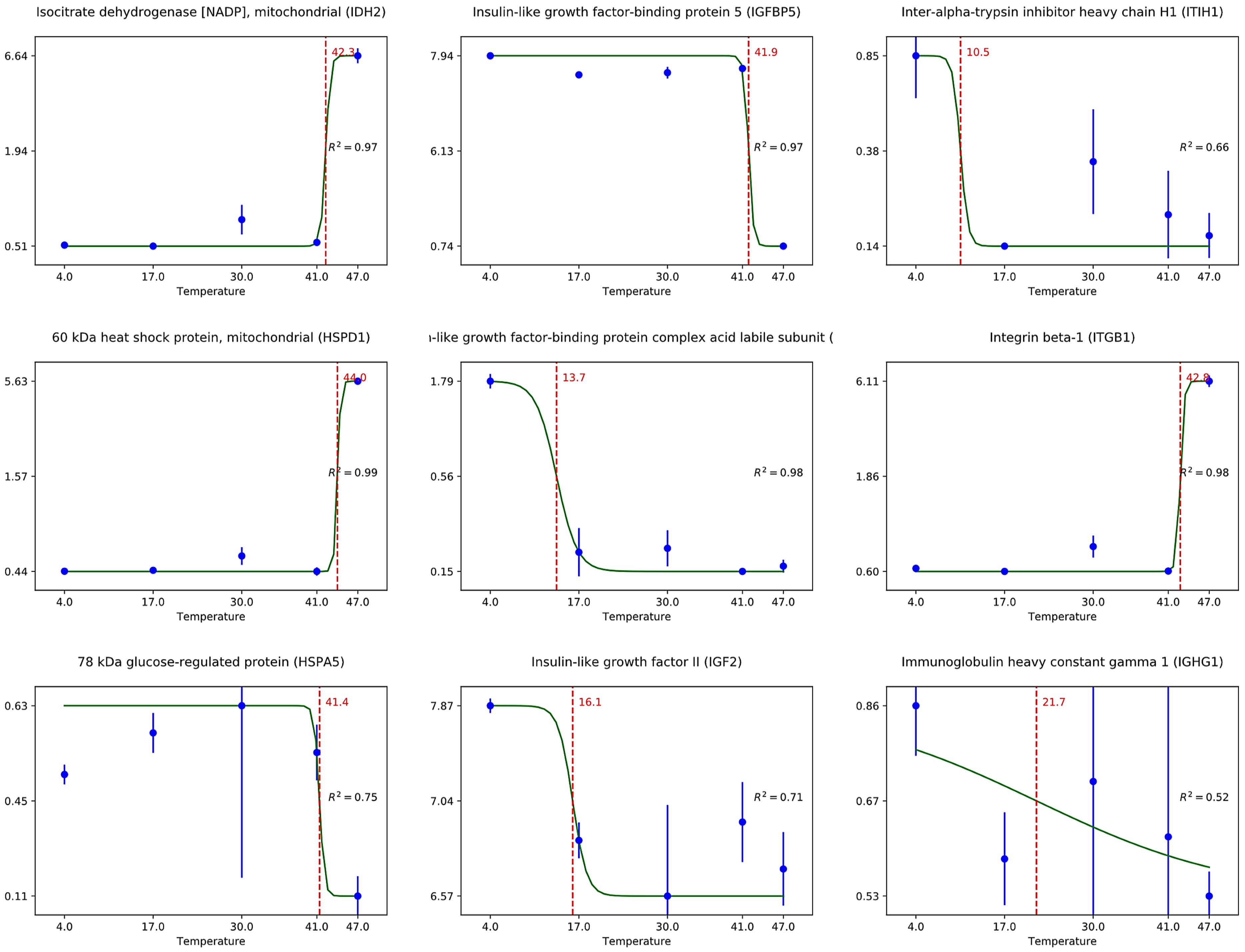

Isocitrate dehydrogenase [NADP], mitochondrial (IDH2)
6.64
1.94
0.51
42.3
$R^2 = 0.97$
4.0
17.0
30.0
41.0
47.0
Temperature
Insulin-like growth factor-binding protein 5 (IGFBP5)
7.94
6.13
0.74
41.9
$R^2 = 0.97$
4.0
17.0
30.0
41.0
47.0
Temperature
Inter-alpha-trypsin inhibitor heavy chain H1 (ITIH1)
0.85
0.38
0.14
10.5
$R^2 = 0.66$
4.0
17.0
30.0
41.0
47.0
Temperature
60 kDa heat shock protein, mitochondrial (HSPD1)
5.63
1.57
0.44
44.0
$R^2 = 0.99$
4.0
17.0
30.0
41.0
47.0
Temperature
-like growth factor-binding protein complex acid labile subunit (
1.79
0.56
0.15
13.7
$R^2 = 0.98$
4.0
17.0
30.0
41.0
47.0
Temperature
Integrin beta-1 (ITGB1)
6.11
1.86
0.60
42.8
$R^2 = 0.98$
4.0
17.0
30.0
41.0
47.0
Temperature
78 kDa glucose-regulated protein (HSPA5)
0.63
0.45
0.11
41.4
$R^2 = 0.75$
4.0
17.0
30.0
41.0
47.0
Temperature
Insulin-like growth factor II (IGF2)
7.87
7.04
6.57
16.1
$R^2 = 0.71$
4.0
17.0
30.0
41.0
47.0
Temperature
Immunoglobulin heavy constant gamma 1 (IGHG1)
0.86
0.67
0.53
21.7
$R^2 = 0.52$
4.0
17.0
30.0
41.0
47.0
Temperature

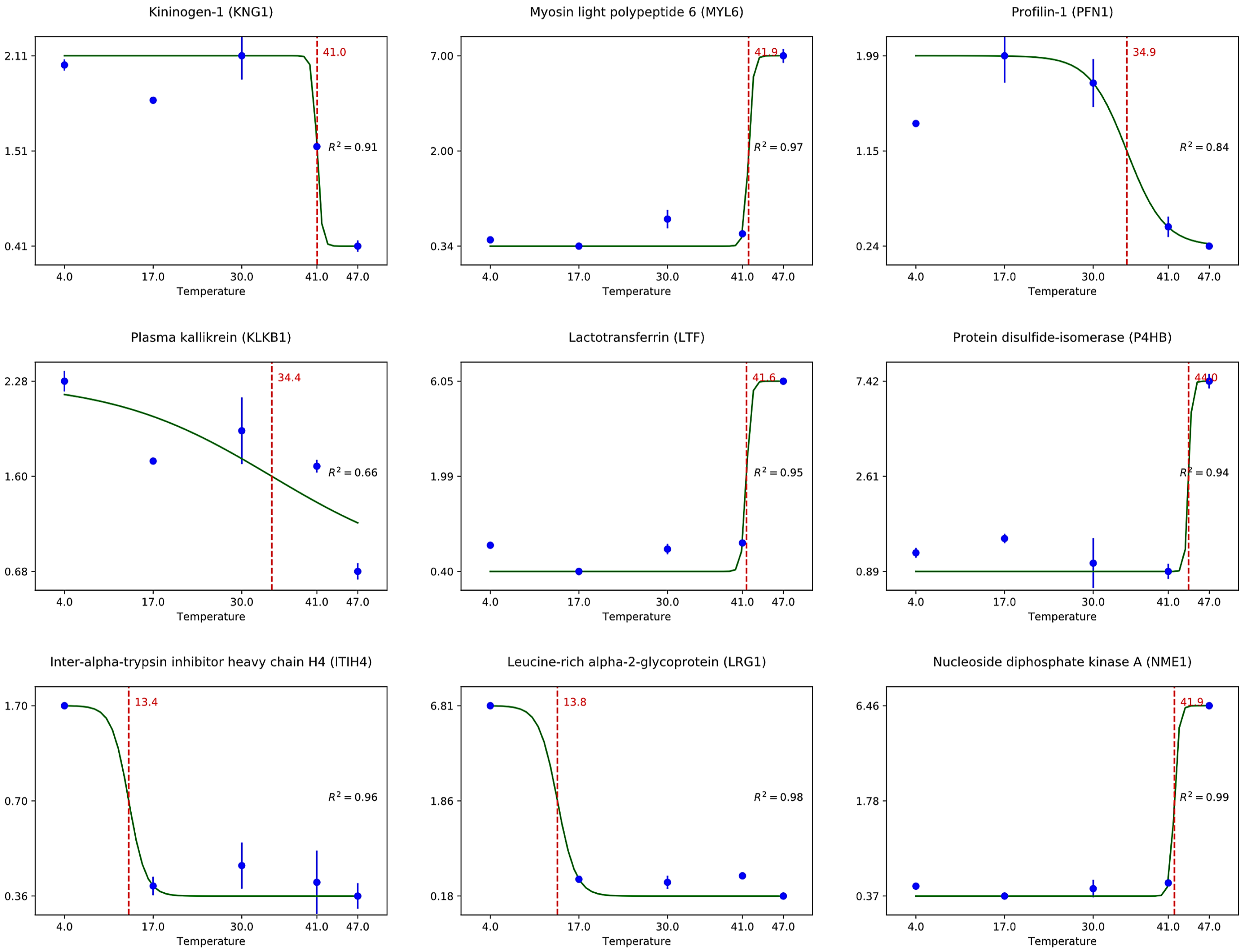
Kininogen-1 (KNG1)
41.0
$R^2 = 0.91$
2.11
1.51
0.41
4.0
17.0
30.0
41.0
47.0
Temperature
Myosin light polypeptide 6 (MYL6)
41.9
$R^2 = 0.97$
7.00
2.00
0.34
Temperature
Profilin-1 (PFN1)
34.9
$R^2 = 0.84$
1.99
1.15
0.24
Temperature
Plasma kallikrein (KLKB1)
34.4
$R^2 = 0.66$
2.28
1.60
0.68
Temperature
Lactotransferrin (LTF)
41.6
$R^2 = 0.95$
6.05
1.99
0.40
Temperature
Protein disulfide-isomerase (P4HB)
44.0
$R^2 = 0.94$
7.42
2.61
0.89
Temperature
Inter-alpha-trypsin inhibitor heavy chain H4 (ITIH4)
13.4
$R^2 = 0.96$
1.70
0.70
0.36
Temperature
Leucine-rich alpha-2-glycoprotein (LRG1)
13.8
$R^2 = 0.98$
6.81
1.86
0.18
Temperature
Nucleoside diphosphate kinase A (NME1)
41.9
$R^2 = 0.99$
6.46
1.78
0.37
Temperature

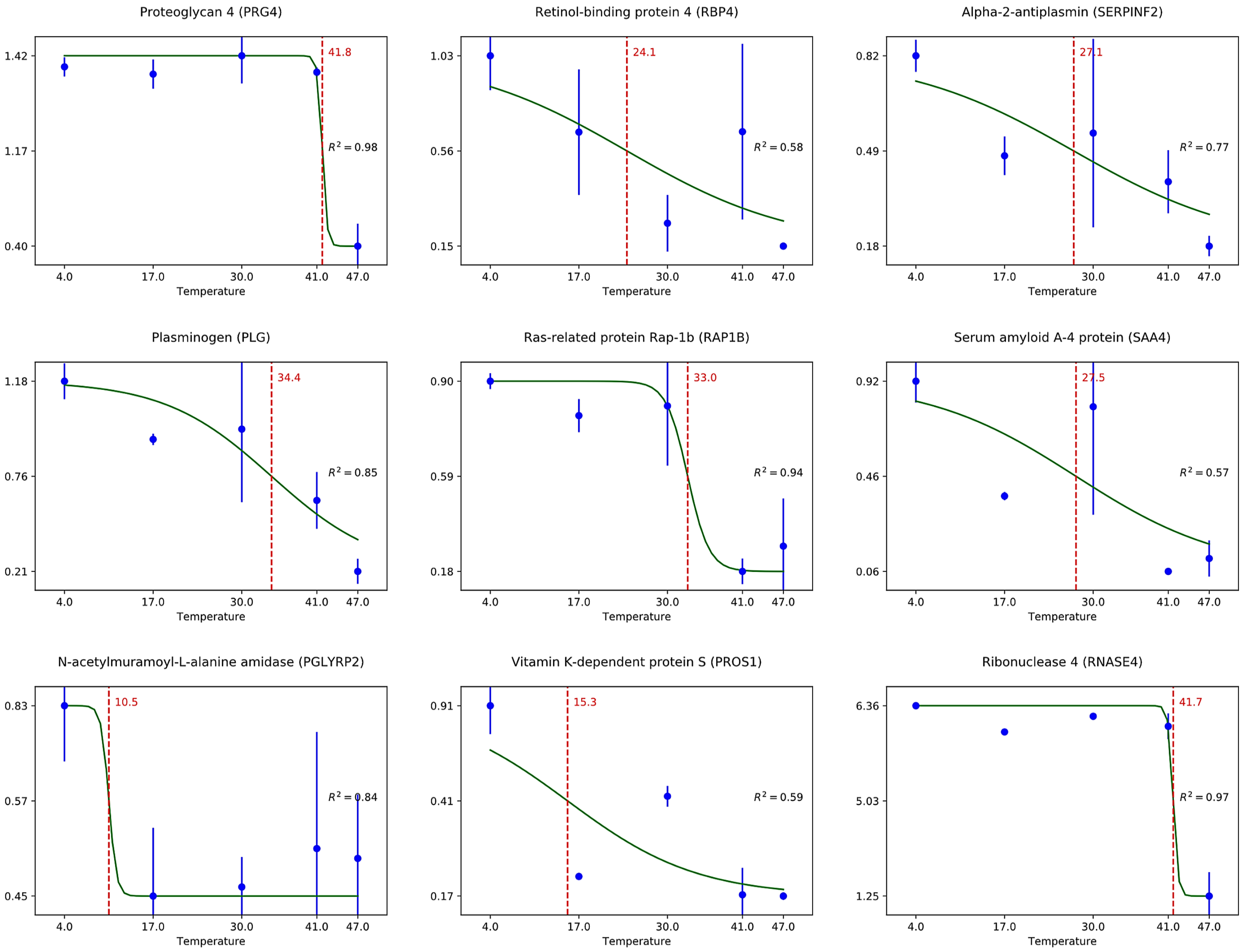
Proteoglycan 4 (PRG4)
1.42
1.17
0.40
41.8
$R^2 = 0.98$
4.0
17.0
30.0
41.0
47.0
Temperature
Retinol-binding protein 4 (RBP4)
1.03
0.56
0.15
24.1
$R^2 = 0.58$
4.0
17.0
30.0
41.0
47.0
Temperature
Alpha-2-antiplasmin (SERPINF2)
0.82
0.49
0.18
27.1
$R^2 = 0.77$
4.0
17.0
30.0
41.0
47.0
Temperature
Plasminogen (PLG)
1.18
0.76
0.21
34.4
$R^2 = 0.85$
4.0
17.0
30.0
41.0
47.0
Temperature
Ras-related protein Rap-1b (RAP1B)
0.90
0.59
0.18
33.0
$R^2 = 0.94$
4.0
17.0
30.0
41.0
47.0
Temperature
Serum amyloid A-4 protein (SAA4)
0.92
0.46
0.06
27.5
$R^2 = 0.57$
4.0
17.0
30.0
41.0
47.0
Temperature
N-acetylmuramoyl-L-alanine amidase (PGLYRP2)
0.83
0.57
0.45
10.5
$R^2 = 0.84$
4.0
17.0
30.0
41.0
47.0
Temperature
Vitamin K-dependent protein S (PROS1)
0.91
0.41
0.17
15.3
$R^2 = 0.59$
4.0
17.0
30.0
41.0
47.0
Temperature
Ribonuclease 4 (RNASE4)
6.36
5.03
1.25
41.7
$R^2 = 0.97$
4.0
17.0
30.0
41.0
47.0
Temperature

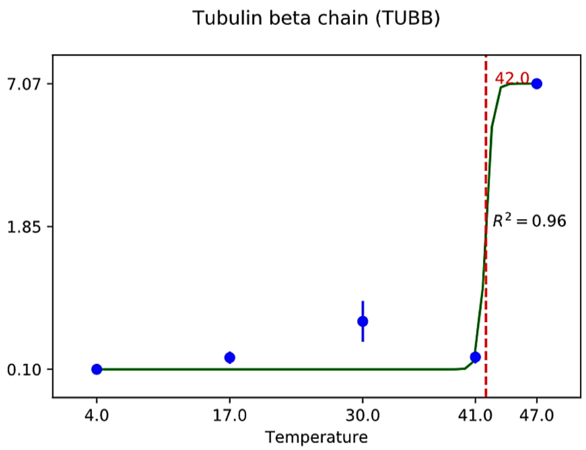
Tubulin beta chain (TUBB)
7.07
1.85
0.10
42.0
$R^2 = 0.96$
4.0
17.0
30.0
41.0
47.0
Temperature

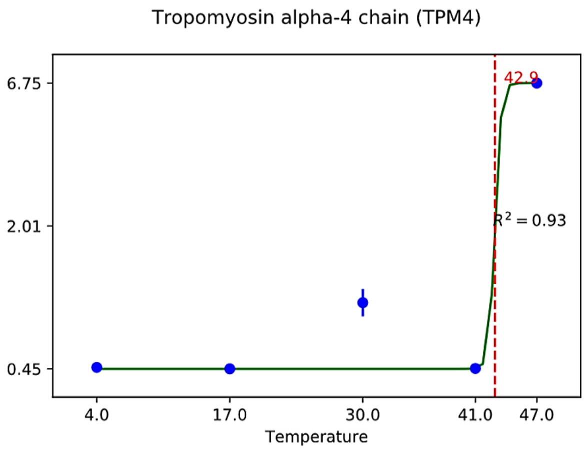
Tropomyosin alpha-4 chain (TPM4)
6.75
2.01
0.45
42.9
$R^2 = 0.93$
4.0
17.0
30.0
41.0
47.0
Temperature

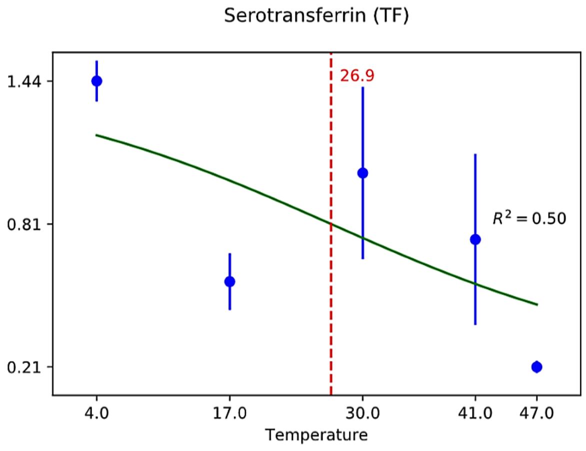
Serotransferrin (TF)
1.44
0.81
0.21
26.9
$R^2 = 0.50$
4.0
17.0
30.0
41.0
47.0
Temperature

Results of lessening analysis

	Difference(pH)	Lessening(pH)	p-value(pH)	FDR(pH)	Difference(Temp)	Lessening(Temp)	p-value(Temp)	FDR(Temp)	Class	Description
**ANDN920101**	0.0851	1.0059	7.749E-01	8.414E-01	0.1143	1.0669	2.608E-01	3.618E-01	Physicochemical properties	alpha-CH chemical shifts (Andersen et al., 1992)
**ARGP820101**	0.0029	0.7459	1.466E-03	6.760E-03	0.0118	0.7065	1.049E-04	1.098E-03	Hydrophobicity	Hydrophobicity index (Argos et al., 1982)
**ARGP820102**	-0.0324	0.9570	7.526E-01	8.255E-01	-0.0634	0.9077	3.353E-01	4.354E-01	Hydrophobicity	Signal sequence helical potential (Argos et al., 1982)
**ARGP820103**	-0.0281	1.0370	5.354E-01	6.318E-01	-0.0724	0.9953	9.056E-01	9.261E-01	Hydrophobicity	Membrane-buried preference parameters (Argos et al., 1982)
**BEGF750101**	0.0084	1.3538	1.750E-06	1.190E-04	-0.0710	1.2759	6.961E-05	8.819E-04	Alpha and turn propensities	Conformational parameter of inner helix (Beghin-Dirkx, 1975)
**BEGF750102**	0.0230	0.8138	1.674E-02	3.976E-02	-0.0039	0.8151	1.418E-02	3.655E-02	Hydrophobicity	Conformational parameter of beta-structure (Beghin-Dirkx, 1975)
**BEGF750103**	0.0125	1.2073	1.904E-03	7.788E-03	0.1185	1.1574	1.137E-02	3.072E-02	Alpha and turn propensities	Conformational parameter of beta-turn (Beghin-Dirkx, 1975)
**BHAR880101**	-0.0973	0.7407	1.852E-03	7.691E-03	-0.0577	0.7579	2.721E-03	1.007E-02	Hydrophobicity	Average flexibility indices (Bhaskaran-Ponnuswamy, 1988)
**BIGC670101**	-0.0143	0.8438	2.710E-02	5.826E-02	-0.0185	0.8550	3.313E-02	7.024E-02	Physicochemical properties	Residue volume (Bigelow, 1967)
**BIOV880101**	0.0590	0.7139	3.814E-04	2.594E-03	0.0576	0.7000	9.036E-05	9.988E-04	Hydrophobicity	Information value for accessibility; average fraction 35% (Biou et al., 1988)
**BIOV880102**	0.0475	0.6721	1.430E-04	1.587E-03	0.0367	0.6526	2.204E-05	4.695E-04	Hydrophobicity	Information value for accessibility; average fraction 23% (Biou et al., 1988)
**BROC820101**	-0.0162	1.1275	6.048E-02	1.058E-01	-0.0626	1.0925	1.451E-01	2.267E-01	Hydrophobicity	Retention coefficient in TFA (Browne et al., 1982)
**BROC820102**	-0.0576	1.1287	5.689E-02	1.008E-01	-0.1004	1.1458	2.520E-02	5.760E-02	Hydrophobicity	Retention coefficient in HFBA (Browne et al., 1982)
**BULH740101**	-0.0225	0.8825	1.983E-01	2.838E-01	-0.0147	0.8486	6.977E-02	1.278E-01	Hydrophobicity	Transfer free energy to surface (Bull-Breese, 1974)
**BULH740102**	-0.0573	0.9580	6.584E-01	7.446E-01	-0.0735	0.9714	7.990E-01	8.520E-01	Physicochemical properties	Apparent partial specific volume (Bull-Breese, 1974)
**BUNA790101**	0.0548	0.8940	2.560E-01	3.544E-01	0.0623	0.8798	1.554E-01	2.374E-01	Alpha and turn propensities	alpha-NH chemical shifts (Bundi-Wuthrich, 1979)
**BUNA790102**	0.1160	0.9051	2.659E-01	3.634E-01	0.1096	1.0315	5.539E-01	6.439E-01	Physicochemical properties	alpha-CH chemical shifts (Bundi-Wuthrich, 1979)
**BUNA790103**	0.0508	0.8536	1.177E-01	1.878E-01	0.0674	0.8426	7.311E-02	1.321E-01	Alpha and turn propensities	Spin-spin coupling constants 3JHalpha-NH (Bundi-Wuthrich, 1979)
**BURA740101**	-0.0392	1.3145	1.827E-05	4.496E-04	-0.0717	1.1398	2.966E-02	6.560E-02	Alpha and turn propensities	Normalized frequency of alpha-helix (Burgess et al., 1974)
**BURA740102**	0.0838	0.9878	9.431E-01	9.629E-01	0.0757	0.9516	6.513E-01	7.245E-01	Beta propensity	Normalized frequency of extended structure (Burgess et al., 1974)
**CHAM810101**	-0.0013	0.9187	4.116E-01	5.101E-01	-0.0502	0.8823	1.940E-01	2.800E-01	Other properties	Steric parameter (Charton, 1981)
**CHAM820101**	0.0218	0.7695	6.014E-04	3.533E-03	0.0185	0.8583	2.673E-02	6.034E-02	Physicochemical properties	Polarizability parameter (Charton-Charton, 1982)
**CHAM820102**	0.1543	0.9102	3.777E-01	4.712E-01	0.1161	0.9077	3.454E-01	4.458E-01	Hydrophobicity	Free energy of solution in water, kcal/mole (Charton-Charton, 1982)
**CHAM830101**	-0.0203	1.1104	1.230E-01	1.939E-01	0.0604	1.0713	2.704E-01	3.704E-01	Alpha and turn propensities	The Chou-Fasman parameter of the coil conformation (Charton-Charton, 1983)
**CHAM830102**	0.1170	1.0303	4.542E-01	5.578E-01	0.1365	0.9939	8.832E-01	9.083E-01	Alpha and turn propensities	A parameter defined from the residuals obtained from the best correlation of
**CHAM830103**	0.0505	0.7753	1.989E-02	4.605E-02	0.0210	0.8936	2.776E-01	3.740E-01	Physicochemical properties	The number of atoms in the side chain labelled 1+1 (Charton-Charton, 1983)
**CHAM830104**	-0.0319	0.9482	5.701E-01	6.598E-01	-0.0720	0.9685	7.544E-01	8.192E-01	Other properties	The number of atoms in the side chain labelled 2+1 (Charton-Charton, 1983)
**CHAM830105**	-0.0141	0.7812	3.486E-03	1.163E-02	-0.0082	0.8537	4.594E-02	9.087E-02	Physicochemical properties	The number of atoms in the side chain labelled 3+1 (Charton-Charton, 1983)
**CHAM830106**	-0.0173	0.8416	5.646E-02	1.004E-01	0.0000	0.8337	3.653E-02	7.556E-02	Physicochemical properties	The number of bonds in the longest chain (Charton-Charton, 1983)
**CHAM830107**	0.0873	1.0240	5.979E-01	6.892E-01	0.0886	1.0019	7.995E-01	8.520E-01	Beta propensity	A parameter of charge transfer capability (Charton-Charton, 1983)
**CHAM830108**	0.0889	0.9726	7.804E-01	8.435E-01	0.1130	0.9978	9.247E-01	9.368E-01	Composition	A parameter of charge transfer donor capability (Charton-Charton, 1983)
**CHOC750101**	-0.0154	0.8483	3.862E-02	7.613E-02	-0.0159	0.8837	9.730E-02	1.659E-01	Physicochemical properties	Average volume of buried residue (Chothia, 1975)
**CHOC760101**	-0.0178	0.8044	7.883E-03	2.121E-02	-0.0144	0.8663	5.814E-02	1.098E-01	Physicochemical properties	Residue accessible surface area in tripeptide (Chothia, 1976)
**CHOC760102**	-0.0440	0.7238	2.182E-03	8.603E-03	-0.0109	0.7036	5.747E-04	3.361E-03	Hydrophobicity	Residue accessible surface area in folded protein (Chothia, 1976)
**CHOC760103**	0.0657	0.7908	7.553E-03	2.086E-02	0.0281	0.8053	8.648E-03	2.502E-02	Hydrophobicity	Proportion of residues 95% buried (Chothia, 1976)
**CHOC760104**	0.0548	0.8468	4.448E-02	8.581E-02	0.0300	0.8103	9.709E-03	2.716E-02	Hydrophobicity	Proportion of residues 100% buried (Chothia, 1976)
**CHOP780101**	-0.0040	1.0471	3.630E-01	4.571E-01	0.0766	1.0267	5.131E-01	6.094E-01	Alpha and turn propensities	Normalized frequency of beta-turn (Chou-Fasman, 1978a)
**CHOP780201**	-0.0640	1.3663	3.461E-06	1.883E-04	-0.1146	1.2092	3.836E-03	1.321E-02	Alpha and turn propensities	Normalized frequency of alpha-helix (Chou-Fasman, 1978b)
**CHOP780202**	0.0568	0.7281	1.736E-04	1.760E-03	0.0486	0.7281	7.709E-05	9.116E-04	Beta propensity	Normalized frequency of beta-sheet (Chou-Fasman, 1978b)
**CHOP780203**	-0.0316	0.9666	8.458E-01	8.935E-01	0.0393	1.0132	7.093E-01	7.779E-01	Alpha and turn propensities	Normalized frequency of beta-turn (Chou-Fasman, 1978b)
**CHOP780204**	0.0080	0.8882	1.980E-01	2.838E-01	-0.0098	0.8194	3.295E-02	7.024E-02	Hydrophobicity	Normalized frequency of N-terminal helix (Chou-Fasman, 1978b)
**CHOP780205**	-0.0590	0.9694	8.753E-01	9.037E-01	-0.0574	0.9251	4.676E-01	5.628E-01	Alpha and turn propensities	Normalized frequency of C-terminal helix (Chou-Fasman, 1978b)
**CHOP780206**	0.0656	0.9334	5.038E-01	6.034E-01	0.0641	0.9979	8.365E-01	8.751E-01	Other properties	Normalized frequency of N-terminal non helical region (Chou-Fasman, 1978b)
**CHOP780207**	-0.0250	1.0821	2.039E-01	2.903E-01	0.0580	1.0613	2.812E-01	3.758E-01	Alpha and turn propensities	Normalized frequency of C-terminal non helical region (Chou-Fasman, 1978b)
**CHOP780208**	-0.0135	0.7785	7.948E-03	2.121E-02	-0.0187	0.7804	4.922E-03	1.573E-02	Beta propensity	Normalized frequency of N-terminal beta-sheet (Chou-Fasman, 1978b)
**CHOP780209**	0.0819	0.7365	7.285E-04	4.003E-03	0.0899	0.7496	7.400E-04	3.798E-03	Beta propensity	Normalized frequency of C-terminal beta-sheet (Chou-Fasman, 1978b)
**CHOP780210**	-0.0434	1.1381	2.621E-02	5.681E-02	0.0593	1.0655	2.161E-01	3.022E-01	Alpha and turn propensities	Normalized frequency of N-terminal non beta region (Chou-Fasman, 1978b)
**CHOP780211**	-0.0354	0.9443	5.058E-01	6.034E-01	-0.0049	0.9341	3.906E-01	4.919E-01	Alpha and turn propensities	Normalized frequency of C-terminal non beta region (Chou-Fasman, 1978b)
**CHOP780212**	0.1135	1.1781	4.609E-03	1.449E-02	0.1548	1.1305	2.009E-02	4.772E-02	Alpha and turn propensities	Frequency of the 1st residue in turn (Chou-Fasman, 1978b)
**CHOP780213**	-0.0772	0.8147	6.883E-02	1.178E-01	-0.0558	0.7975	3.465E-02	7.306E-02	Alpha and turn propensities	Frequency of the 2nd residue in turn (Chou-Fasman, 1978b)
**CHOP780214**	0.0983	1.0913	1.463E-01	2.242E-01	0.1443	1.1475	2.079E-02	4.892E-02	Other properties	Frequency of the 3rd residue in turn (Chou-Fasman, 1978b)
**CHOP780215**	0.0665	1.2936	1.529E-04	1.664E-03	0.1453	1.2624	3.102E-04	2.150E-03	Alpha and turn propensities	Frequency of the 4th residue in turn (Chou-Fasman, 1978b)

**CHOP780216**	-0.0298	0.9665	7.684E-01	8.377E-01	0.0335	0.9396	4.906E-01	5.866E-01	Alpha and turn propensities	Normalized frequency of the 2nd and 3rd residues in turn (Chou-Fasman, 1978b)
**CIDH920101**	0.0709	0.7624	5.339E-03	1.614E-02	0.0518	0.7472	1.803E-03	7.212E-03	Hydrophobicity	Normalized hydrophobicity scales for alpha-proteins (Cid et al., 1992)
**CIDH920102**	0.0463	0.6879	1.981E-04	1.858E-03	0.0463	0.6588	2.216E-05	4.695E-04	Hydrophobicity	Normalized hydrophobicity scales for beta-proteins (Cid et al., 1992)
**CIDH920103**	-0.0022	0.6936	2.700E-05	5.650E-04	-0.0029	0.6446	3.716E-07	2.527E-05	Hydrophobicity	Normalized hydrophobicity scales for alpha+beta-proteins (Cid et al., 1992)
**CIDH920104**	0.0489	0.6553	5.708E-06	2.588E-04	0.0597	0.5946	2.286E-08	4.146E-06	Hydrophobicity	Normalized hydrophobicity scales for alpha/beta-proteins (Cid et al., 1992)
**CIDH920105**	0.0155	0.6813	2.366E-05	5.149E-04	0.0400	0.6345	3.626E-07	2.527E-05	Hydrophobicity	Normalized average hydrophobicity scales (Cid et al., 1992)
**COHE430101**	-0.0794	1.0667	2.279E-01	3.187E-01	-0.0865	1.0820	1.477E-01	2.289E-01	Physicochemical properties	Partial specific volume (Cohn-Edsall, 1943)
**CRAJ730101**	-0.0293	1.1973	8.649E-03	2.273E-02	-0.1072	1.1233	7.522E-02	1.351E-01	Alpha and turn propensities	Normalized frequency of middle helix (Crawford et al., 1973)
**CRAJ730102**	0.0146	0.7787	7.883E-03	2.121E-02	0.0102	0.8284	3.022E-02	6.656E-02	Beta propensity	Normalized frequency of beta-sheet (Crawford et al., 1973)
**CRAJ730103**	0.0404	1.2403	1.167E-04	1.476E-03	0.0927	1.2034	5.804E-04	3.361E-03	Alpha and turn propensities	Normalized frequency of turn (Crawford et al., 1973)
**DAWD720101**	-0.0272	0.7592	1.151E-03	5.695E-03	-0.0394	0.8861	1.264E-01	2.044E-01	Physicochemical properties	Size (Dawson, 1972)
**DAYM780101**	-0.0905	1.0317	5.598E-01	6.507E-01	-0.0902	1.0957	1.385E-01	2.196E-01	Composition	Amino acid composition (Dayhoff et al., 1978a)
**DAYM780201**	-0.0601	0.9443	5.463E-01	6.419E-01	-0.1647	1.0308	5.048E-01	6.009E-01	Physicochemical properties	Relative mutability (Dayhoff et al., 1978b)
**DESM900101**	0.0638	0.7219	8.457E-04	4.555E-03	0.0470	0.7243	5.494E-04	3.284E-03	Hydrophobicity	Membrane preference for cytochrome b: MPH89 (Degli Esposti et al., 1990)
**DESM900102**	0.0463	0.7555	7.541E-03	2.086E-02	0.0330	0.7230	1.442E-03	6.227E-03	Hydrophobicity	Average membrane preference: AMP07 (Degli Esposti et al., 1990)
**EISD840101**	0.0634	0.7744	1.351E-02	3.296E-02	0.0157	0.7749	8.863E-03	2.551E-02	Hydrophobicity	Consensus normalized hydrophobicity scale (Eisenberg, 1984)
**EISD860101**	0.0585	0.8259	4.916E-02	9.230E-02	0.0057	0.7343	9.345E-04	4.459E-03	Hydrophobicity	Solvation free energy (Eisenberg-McLachlan, 1986)
**EISD860102**	-0.1361	0.7864	2.000E-02	4.611E-02	-0.0838	0.8205	4.191E-02	8.443E-02	Hydrophobicity	Atom-based hydrophobic moment (Eisenberg-McLachlan, 1986)
**EISD860103**	0.0482	0.8149	2.755E-02	5.877E-02	0.0376	0.7733	4.619E-03	1.496E-02	Hydrophobicity	Direction of hydrophobic moment (Eisenberg-McLachlan, 1986)
**FASG760101**	0.0276	0.7926	7.431E-03	2.073E-02	0.0276	0.8823	1.266E-01	2.044E-01	Physicochemical properties	Molecular weight (Fasman, 1976)
**FASG760102**	0.0641	1.0246	5.620E-01	6.519E-01	0.0169	1.0028	7.940E-01	8.502E-01	Hydrophobicity	Melting point (Fasman, 1976)
**FASG760103**	0.0039	0.9557	6.969E-01	7.769E-01	0.0039	0.9421	5.570E-01	6.460E-01	Alpha and turn propensities	Optical rotation (Fasman, 1976)
**FASG760104**	-0.0896	0.8885	2.944E-01	3.906E-01	-0.0655	0.9690	9.023E-01	9.243E-01	Alpha and turn propensities	pK-N (Fasman, 1976)
**FASG760105**	0.0138	1.0694	2.681E-01	3.655E-01	-0.0607	1.0192	6.007E-01	6.802E-01	Hydrophobicity	pK-C (Fasman, 1976)
**FAUJ830101**	0.0557	0.7691	6.500E-03	1.871E-02	0.0299	0.6794	6.971E-05	8.819E-04	Hydrophobicity	Hydrophobic parameter pi (Fauchere-Pliska, 1983)
**FAUJ880101**	0.0073	0.8437	6.839E-02	1.177E-01	0.0088	0.8073	1.697E-02	4.159E-02	Physicochemical properties	Graph shape index (Fauchere et al., 1988)
**FAUJ880102**	0.0136	0.9024	2.986E-01	3.950E-01	-0.0354	0.9214	3.880E-01	4.897E-01	Other properties	Smoothed upsilon steric parameter (Fauchere et al., 1988)
**FAUJ880103**	0.0185	0.7984	5.326E-03	1.614E-02	0.0185	0.8213	1.069E-02	2.951E-02	Physicochemical properties	Normalized van der Waals volume (Fauchere et al., 1988)
**FAUJ880104**	-0.0688	0.8191	1.293E-02	3.211E-02	-0.0464	0.8892	1.218E-01	1.978E-01	Physicochemical properties	STERIMOL length of the side chain (Fauchere et al., 1988)
**FAUJ880105**	0.0090	0.8205	5.742E-02	1.014E-01	-0.0201	0.9000	3.006E-01	3.969E-01	Physicochemical properties	STERIMOL minimum width of the side chain (Fauchere et al., 1988)
**FAUJ880106**	-0.0015	0.7802	8.716E-03	2.280E-02	0.0029	0.8769	1.401E-01	2.210E-01	Physicochemical properties	STERIMOL maximum width of the side chain (Fauchere et al., 1988)
**FAUJ880107**	0.0122	0.9889	9.723E-01	9.743E-01	-0.0329	0.9528	6.468E-01	7.225E-01	Other properties	N.m.r. chemical shift of alpha-carbon (Fauchere et al., 1988)
**FAUJ880108**	0.0460	0.9607	7.264E-01	8.016E-01	0.0460	1.0239	6.014E-01	6.802E-01	Hydrophobicity	Localized electrical effect (Fauchere et al., 1988)
**FAUJ880109**	-0.0771	0.7774	7.084E-03	1.997E-02	-0.0469	0.7945	8.223E-03	2.392E-02	Hydrophobicity	Number of hydrogen bond donors (Fauchere et al., 1988)
**FAUJ880110**	-0.0064	0.9326	5.026E-01	6.034E-01	-0.0232	0.8435	7.190E-02	1.313E-01	Hydrophobicity	Number of full nonbonding orbitals (Fauchere et al., 1988)
**FAUJ880111**	-0.1172	0.7370	1.566E-03	6.926E-03	-0.0338	0.8309	3.265E-02	6.992E-02	Hydrophobicity	Positive charge (Fauchere et al., 1988)
**FAUJ880112**	-0.0162	0.9069	3.568E-01	4.515E-01	-0.0155	0.8610	1.316E-01	2.106E-01	Hydrophobicity	Negative charge (Fauchere et al., 1988)
**FAUJ880113**	0.0248	1.0564	4.156E-01	5.127E-01	0.0128	1.0266	6.199E-01	6.968E-01	Alpha and turn propensities	pK-a(RCOOH) (Fauchere et al., 1988)
**FINA770101**	-0.0144	1.0047	7.906E-01	8.466E-01	-0.0666	1.0309	5.441E-01	6.352E-01	Alpha and turn propensities	Helix-coil equilibrium constant (Finkelstein-Ptitsyn, 1977)
**FINA910101**	0.0767	0.8830	1.525E-01	2.317E-01	0.0581	0.9436	4.856E-01	5.832E-01	Hydrophobicity	Helix initiation parameter at posision i-1 (Finkelstein et al., 1991)
**FINA910102**	-0.0481	0.9099	3.734E-01	4.680E-01	-0.0504	0.8393	7.801E-02	1.382E-01	Alpha and turn propensities	Helix initiation parameter at posision i,i+1,i+2 (Finkelstein et al., 1991)
**FINA910103**	-0.1341	0.7279	1.371E-03	6.373E-03	-0.0992	0.8012	1.620E-02	4.092E-02	Hydrophobicity	Helix termination parameter at posision j-2,j-1,j (Finkelstein et al., 1991)
**FINA910104**	-0.0350	0.7603	7.699E-03	2.115E-02	0.0170	0.8671	1.108E-01	1.838E-01	Hydrophobicity	Helix termination parameter at posision j+1 (Finkelstein et al., 1991)
**GARJ730101**	0.1686	0.7607	2.931E-03	1.029E-02	0.1035	0.8079	1.209E-02	3.209E-02	Physicochemical properties	Partition coefficient (Garel et al., 1973)
**GEIM800101**	-0.0782	1.1909	9.714E-03	2.493E-02	-0.1003	1.1207	7.680E-02	1.365E-01	Alpha and turn propensities	Alpha-helix indices (Geisow-Roberts, 1980)
**GEIM800102**	0.0345	0.7826	1.799E-02	4.238E-02	0.0225	0.8839	1.896E-01	2.744E-01	Alpha and turn propensities	Alpha-helix indices for alpha-proteins (Geisow-Roberts, 1980)
**GEIM800103**	0.0460	1.0375	4.866E-01	5.907E-01	-0.0003	0.9347	5.330E-01	6.235E-01	Alpha and turn propensities	Alpha-helix indices for beta-proteins (Geisow-Roberts, 1980)
**GEIM800104**	-0.0694	1.1039	1.149E-01	1.843E-01	-0.1018	0.9710	8.124E-01	8.595E-01	Alpha and turn propensities	Alpha-helix indices for alpha/beta-proteins (Geisow-Roberts, 1980)
**GEIM800105**	0.0997	0.9563	7.089E-01	7.870E-01	0.0982	0.8695	1.095E-01	1.821E-01	Beta propensity	Beta-strand indices (Geisow-Roberts, 1980)
**GEIM800106**	-0.0092	0.8456	4.498E-02	8.617E-02	-0.0139	0.8310	2.086E-02	4.892E-02	Beta propensity	Beta-strand indices for beta-proteins (Geisow-Roberts, 1980)
**GEIM800107**	0.0427	0.7517	1.508E-03	6.838E-03	0.0449	0.7445	6.157E-04	3.472E-03	Beta propensity	Beta-strand indices for alpha/beta-proteins (Geisow-Roberts, 1980)
**GEIM800108**	0.0004	0.9222	4.494E-01	5.531E-01	0.0611	0.9171	3.873E-01	4.897E-01	Alpha and turn propensities	Aperiodic indices (Geisow-Roberts, 1980)
**GEIM800109**	-0.0233	0.8087	3.475E-02	7.054E-02	0.0072	0.8509	8.753E-02	1.521E-01	Alpha and turn propensities	Aperiodic indices for alpha-proteins (Geisow-Roberts, 1980)

**GEIM800110**	-0.0161	0.7664	1.317E-02	3.228E-02	0.0164	0.7800	1.337E-02	3.463E-02	Beta propensity	Aperiodic indices for beta-proteins (Geisow-Roberts, 1980)
**GEIM800111**	-0.0060	0.9486	6.485E-01	7.380E-01	0.0329	0.9235	4.029E-01	5.061E-01	Alpha and turn propensities	Aperiodic indices for alpha/beta-proteins (Geisow-Roberts, 1980)
**GOLD730101**	-0.0124	0.7612	1.904E-03	7.788E-03	-0.0122	0.7219	1.339E-04	1.271E-03	Hydrophobicity	Hydrophobicity factor (Goldsack-Chalifoux, 1973)
**GOLD730102**	-0.0145	0.8393	3.251E-02	6.675E-02	-0.0145	0.8631	5.971E-02	1.124E-01	Physicochemical properties	Residue volume (Goldsack-Chalifoux, 1973)
**GRAR740101**	0.0132	1.2048	5.401E-03	1.623E-02	0.0460	1.2080	3.067E-03	1.112E-02	Hydrophobicity	Composition (Grantham, 1974)
**GRAR740102**	-0.0442	0.8623	1.167E-01	1.868E-01	-0.0330	0.7837	8.920E-03	2.554E-02	Hydrophobicity	Polarity (Grantham, 1974)
**GRAR740103**	-0.0058	0.8244	3.618E-02	7.235E-02	-0.0127	0.8674	1.018E-01	1.725E-01	Physicochemical properties	Volume (Grantham, 1974)
**GUYH850101**	-0.0824	0.6916	2.998E-04	2.260E-03	-0.0797	0.6513	1.586E-05	3.751E-04	Hydrophobicity	Partition energy (Guy, 1985)
**HOPA770101**	0.0062	0.8827	1.762E-01	2.590E-01	0.0144	0.8617	8.989E-02	1.552E-01	Hydrophobicity	Hydration number (Hopfinger, 1971), Cited by Charton-Charton (1982)
**HOPT810101**	-0.1104	0.8109	5.016E-02	9.345E-02	-0.0488	0.7757	1.260E-02	3.313E-02	Hydrophobicity	Hydrophilicity value (Hopp-Woods, 1981)
**HUTJ700101**	0.0941	0.7795	2.894E-03	1.022E-02	0.0669	0.7898	2.873E-03	1.056E-02	Composition	Heat capacity (Hutchens, 1970)
**HUTJ700102**	-0.0234	0.7748	2.752E-03	9.913E-03	-0.0019	0.8815	1.202E-01	1.958E-01	Physicochemical properties	Absolute entropy (Hutchens, 1970)
**HUTJ700103**	-0.0476	0.8391	4.781E-02	9.095E-02	-0.0310	0.9067	2.656E-01	3.667E-01	Physicochemical properties	Entropy of formation (Hutchens, 1970)
**ISOY800101**	-0.0505	1.2602	1.024E-03	5.355E-03	-0.0980	1.1170	1.035E-01	1.738E-01	Alpha and turn propensities	Normalized relative frequency of alpha-helix (Isogai et al., 1980)
**ISOY800102**	0.0900	1.0466	4.757E-01	5.815E-01	0.0863	0.9237	4.528E-01	5.487E-01	Beta propensity	Normalized relative frequency of extended structure (Isogai et al., 1980)
**ISOY800103**	0.0116	0.8771	1.808E-01	2.645E-01	0.0491	0.9818	9.316E-01	9.419E-01	Alpha and turn propensities	Normalized relative frequency of bend (Isogai et al., 1980)
**ISOY800104**	-0.0841	0.8443	8.515E-02	1.430E-01	-0.0608	0.8412	6.015E-02	1.128E-01	Alpha and turn propensities	Normalized relative frequency of bend R (Isogai et al., 1980)
**ISOY800105**	0.0586	0.9994	7.864E-01	8.452E-01	0.1588	1.1387	3.895E-02	7.966E-02	Other properties	Normalized relative frequency of bend S (Isogai et al., 1980)
**ISOY800106**	-0.0816	0.9081	2.924E-01	3.889E-01	-0.1156	1.0315	4.406E-01	5.386E-01	Alpha and turn propensities	Normalized relative frequency of helix end (Isogai et al., 1980)
**ISOY800107**	-0.0176	0.9607	7.877E-01	8.452E-01	-0.0145	0.9241	4.513E-01	5.487E-01	Hydrophobicity	Normalized relative frequency of double bend (Isogai et al., 1980)
**ISOY800108**	0.0606	1.0652	3.333E-01	4.282E-01	0.1311	1.2203	3.773E-03	1.307E-02	Other properties	Normalized relative frequency of coil (Isogai et al., 1980)
**JANJ780101**	-0.0635	0.7533	2.120E-03	8.420E-03	-0.0432	0.7484	1.046E-03	4.853E-03	Hydrophobicity	Average accessible surface area (Janin et al., 1978)
**JANJ780102**	0.0754	0.7389	4.889E-03	1.511E-02	0.0561	0.7222	1.787E-03	7.212E-03	Hydrophobicity	Percentage of buried residues (Janin et al., 1978)
**JANJ780103**	-0.0609	0.7607	2.845E-03	1.018E-02	-0.0443	0.7381	6.537E-04	3.521E-03	Hydrophobicity	Percentage of exposed residues (Janin et al., 1978)
**JANJ790101**	0.1008	0.7786	5.798E-03	1.714E-02	0.1138	0.7631	1.792E-03	7.212E-03	Hydrophobicity	Ratio of buried and accessible molar fractions (Janin, 1979)
**JANJ790102**	0.0656	0.7267	2.093E-03	8.420E-03	0.0416	0.7138	7.379E-04	3.798E-03	Hydrophobicity	Transfer free energy (Janin, 1979)
**JOND750101**	0.0031	0.7455	1.591E-03	6.954E-03	0.0105	0.7078	1.378E-04	1.271E-03	Hydrophobicity	Hydrophobicity (Jones, 1975)
**JOND750102**	0.0100	1.0872	2.195E-01	3.085E-01	-0.0219	1.0988	1.577E-01	2.397E-01	Hydrophobicity	pK (-COOH) (Jones, 1975)
**JOND920101**	-0.1033	1.2556	2.551E-04	2.055E-03	-0.0923	1.2442	2.746E-04	1.965E-03	Composition	Relative frequency of occurrence (Jones et al., 1992)
**JOND920102**	-0.1287	0.9535	6.529E-01	7.416E-01	-0.1780	1.0717	2.121E-01	2.974E-01	Physicochemical properties	Relative mutability (Jones et al., 1992)
**JUKT750101**	-0.0380	1.1645	1.667E-02	3.976E-02	-0.0456	1.1973	2.718E-03	1.007E-02	Composition	Amino acid distribution (Jukes et al., 1975)
**JUNJ780101**	-0.0614	1.0118	6.939E-01	7.751E-01	-0.0587	1.0967	1.170E-01	1.928E-01	Composition	Sequence frequency (Jungck, 1978)
**KANM800101**	-0.0634	1.2716	4.080E-04	2.674E-03	-0.0827	1.1411	5.067E-02	9.809E-02	Alpha and turn propensities	Average relative probability of helix (Kanehisa-Tsong, 1980)
**KANM800102**	0.0687	0.7377	6.607E-04	3.729E-03	0.0584	0.7261	1.655E-04	1.412E-03	Beta propensity	Average relative probability of beta-sheet (Kanehisa-Tsong, 1980)
**KANM800103**	-0.0259	1.2205	1.680E-03	7.196E-03	-0.0864	1.1328	3.979E-02	8.107E-02	Alpha and turn propensities	Average relative probability of inner helix (Kanehisa-Tsong, 1980)
**KANM800104**	0.0714	0.7757	6.421E-03	1.863E-02	0.0654	0.7562	1.788E-03	7.212E-03	Beta propensity	Average relative probability of inner beta-sheet (Kanehisa-Tsong, 1980)
**KARP850101**	-0.0688	0.6577	1.278E-04	1.533E-03	-0.0711	0.6719	1.428E-04	1.295E-03	Hydrophobicity	Flexibility parameter for no rigid neighbors (Karplus-Schulz, 1985)
**KARP850102**	-0.0454	0.6526	1.323E-05	3.753E-04	-0.0404	0.6968	6.558E-05	8.819E-04	Hydrophobicity	Flexibility parameter for one rigid neighbor (Karplus-Schulz, 1985)
**KARP850103**	0.0170	0.8800	1.643E-01	2.467E-01	0.0721	0.9465	5.930E-01	6.763E-01	Composition	Flexibility parameter for two rigid neighbors (Karplus-Schulz, 1985)
**KHAG800101**	-0.1031	0.9109	3.459E-01	4.397E-01	-0.0862	0.8801	1.489E-01	2.301E-01	Hydrophobicity	The Kerr-constant increments (Khanarian-Moore, 1980)
**KLEP840101**	-0.1167	0.8303	9.760E-02	1.604E-01	-0.0387	0.8330	8.933E-02	1.548E-01	Hydrophobicity	Net charge (Klein et al., 1984)
**KRIW710101**	-0.0775	0.6953	1.216E-03	5.904E-03	-0.0619	0.8252	5.783E-02	1.098E-01	Hydrophobicity	Side chain interaction parameter (Krigbaum-Rubin, 1971)
**KRIW790101**	-0.0830	0.6496	1.480E-06	1.150E-04	-0.0746	0.6735	2.601E-06	1.014E-04	Hydrophobicity	Side chain interaction parameter (Krigbaum-Komoriya, 1979)
**KRIW790102**	-0.0558	0.6523	8.819E-06	3.198E-04	-0.0325	0.6958	4.516E-05	7.020E-04	Hydrophobicity	Fraction of site occupied by water (Krigbaum-Komoriya, 1979)
**KRIW790103**	-0.0053	0.7841	2.751E-03	9.913E-03	-0.0081	0.8585	4.068E-02	8.257E-02	Physicochemical properties	Side chain volume (Krigbaum-Komoriya, 1979)
**KYTJ820101**	0.0502	0.8424	5.514E-02	9.899E-02	0.0179	0.8033	1.100E-02	2.992E-02	Hydrophobicity	Hydropathy index (Kyte-Doolittle, 1982)
**LAWE840101**	-0.0314	0.8925	2.154E-01	3.044E-01	-0.0463	0.8866	1.550E-01	2.374E-01	Hydrophobicity	Transfer free energy, CHP/water (Lawson et al., 1984)
**LEVM760101**	-0.0935	0.7939	1.846E-02	4.310E-02	-0.0388	0.7533	2.566E-03	9.695E-03	Hydrophobicity	Hydrophobic parameter (Levitt, 1976)
**LEVM760102**	-0.0148	0.7901	6.439E-03	1.863E-02	-0.0134	0.8504	4.424E-02	8.783E-02	Physicochemical properties	Distance between C-alpha and centroid of side chain (Levitt, 1976)
**LEVM760103**	-0.0460	1.0143	7.747E-01	8.414E-01	-0.0656	1.0288	6.072E-01	6.853E-01	Other properties	Side chain angle theta(AAR) (Levitt, 1976)
**LEVM760104**	-0.0152	0.8217	3.631E-02	7.235E-02	0.0102	0.8929	2.015E-01	2.885E-01	Other properties	Side chain torsion angle phi(AAAR) (Levitt, 1976)
**LEVM760105**	-0.0073	0.7907	5.057E-03	1.554E-02	0.0025	0.8546	4.741E-02	9.309E-02	Physicochemical properties	Radius of gyration of side chain (Levitt, 1976)
**LEVM760106**	-0.0117	0.8169	1.837E-02	4.309E-02	-0.0176	0.8743	9.278E-02	1.592E-01	Physicochemical properties	van der Waals parameter R0 (Levitt, 1976)

**LEVM760107**	0.0982	0.7592	5.723E-04	3.421E-03	0.0948	0.7641	3.655E-04	2.425E-03	Physicochemical properties	van der Waals parameter epsilon (Levitt, 1976)
**LEVM780101**	-0.0713	1.1462	3.213E-02	6.626E-02	-0.0858	1.0615	2.710E-01	3.704E-01	Alpha and turn propensities	Normalized frequency of alpha-helix, with weights (Levitt, 1978)
**LEVM780102**	0.0171	0.7835	4.178E-03	1.337E-02	-0.0024	0.8511	4.322E-02	8.644E-02	Beta propensity	Normalized frequency of beta-sheet, with weights (Levitt, 1978)
**LEVM780103**	-0.0105	0.9643	8.083E-01	8.605E-01	0.0199	0.9299	4.873E-01	5.840E-01	Alpha and turn propensities	Normalized frequency of reverse turn, with weights (Levitt, 1978)
**LEVM780104**	-0.0574	1.1838	8.247E-03	2.189E-02	-0.0802	1.1502	2.311E-02	5.350E-02	Alpha and turn propensities	Normalized frequency of alpha-helix, unweighted (Levitt, 1978)
**LEVM780105**	0.0914	0.7945	1.024E-02	2.592E-02	0.0407	0.7803	4.019E-03	1.361E-02	Beta propensity	Normalized frequency of beta-sheet, unweighted (Levitt, 1978)
**LEVM780106**	-0.0164	0.8906	2.471E-01	3.438E-01	0.0309	0.8757	1.594E-01	2.414E-01	Alpha and turn propensities	Normalized frequency of reverse turn, unweighted (Levitt, 1978)
**LEWP710101**	0.0003	0.9575	6.766E-01	7.604E-01	0.0200	1.0369	5.158E-01	6.103E-01	Alpha and turn propensities	Frequency of occurrence in beta-bends (Lewis et al., 1971)
**LIFS790101**	0.0320	0.6792	1.955E-04	1.858E-03	0.0066	0.7155	4.941E-04	3.154E-03	Beta propensity	Conformational preference for all beta-strands (Lifson-Sander, 1979)
**LIFS790102**	0.0058	0.8016	7.954E-03	2.121E-02	0.0106	0.8468	3.079E-02	6.672E-02	Beta propensity	Conformational preference for parallel beta-strands (Lifson-Sander, 1979)
**LIFS790103**	0.0817	0.7229	6.402E-03	1.863E-02	0.0273	0.7201	4.093E-03	1.366E-02	Beta propensity	Conformational preference for antiparallel beta-strands (Lifson-Sander, 1979)
**MANP780101**	0.0506	0.6729	1.901E-05	4.496E-04	0.0506	0.6398	7.130E-07	4.310E-05	Hydrophobicity	Average surrounding hydrophobicity (Manavalan-Ponnuswamy, 1978)
**MAXF760101**	-0.0596	1.2350	1.843E-03	7.691E-03	-0.0984	1.1102	1.066E-01	1.784E-01	Alpha and turn propensities	Normalized frequency of alpha-helix (Maxfield-Scheraga, 1976)
**MAXF760102**	0.0757	1.0205	6.562E-01	7.437E-01	0.0514	0.8940	3.466E-01	4.458E-01	Beta propensity	Normalized frequency of extended structure (Maxfield-Scheraga, 1976)
**MAXF760103**	0.0982	0.9923	9.579E-01	9.684E-01	0.0838	0.9707	7.762E-01	8.362E-01	Other properties	Normalized frequency of zeta R (Maxfield-Scheraga, 1976)
**MAXF760104**	0.0857	1.1240	9.045E-02	1.502E-01	0.1591	1.1536	3.044E-02	6.671E-02	Other properties	Normalized frequency of left-handed alpha-helix (Maxfield-Scheraga, 1976)
**MAXF760105**	0.0721	0.8409	9.055E-02	1.502E-01	0.0919	0.9686	8.554E-01	8.921E-01	Other properties	Normalized frequency of zeta L (Maxfield-Scheraga, 1976)
**MAXF760106**	-0.0640	0.8221	2.374E-02	5.272E-02	-0.0749	0.8487	4.761E-02	9.309E-02	Alpha and turn propensities	Normalized frequency of alpha region (Maxfield-Scheraga, 1976)
**MCMT640101**	0.0812	0.8491	7.949E-02	1.347E-01	0.0870	0.8832	1.807E-01	2.656E-01	Physicochemical properties	Refractivity (McMeekin et al., 1964), Cited by Jones (1975)
**MEEJ800101**	-0.0143	0.9863	9.508E-01	9.650E-01	-0.0191	0.8602	1.493E-01	2.301E-01	Hydrophobicity	Retention coefficient in HPLC, pH7.4 (Meek, 1980)
**MEEJ800102**	0.0396	0.8227	3.836E-02	7.588E-02	0.0127	0.7809	7.138E-03	2.182E-02	Hydrophobicity	Retention coefficient in HPLC, pH2.1 (Meek, 1980)
**MEEJ810101**	0.0489	0.7449	1.490E-03	6.812E-03	0.0309	0.7159	2.321E-04	1.779E-03	Hydrophobicity	Retention coefficient in NaClO4 (Meek-Rossetti, 1981)
**MEEJ810102**	0.0633	0.7418	2.405E-03	9.278E-03	0.0364	0.7264	5.437E-04	3.284E-03	Hydrophobicity	Retention coefficient in NaH2PO4 (Meek-Rossetti, 1981)
**MEIH800101**	-0.0301	0.7240	3.988E-04	2.674E-03	-0.0258	0.6899	3.313E-05	6.007E-04	Hydrophobicity	Average reduced distance for C-alpha (Meirovitch et al., 1980)
**MEIH800102**	-0.0556	0.7084	6.041E-04	3.533E-03	-0.0289	0.7227	6.525E-04	3.521E-03	Hydrophobicity	Average reduced distance for side chain (Meirovitch et al., 1980)
**MEIH800103**	0.0298	0.7568	3.409E-03	1.145E-02	0.0237	0.7547	2.219E-03	8.561E-03	Hydrophobicity	Average side chain orientation angle (Meirovitch et al., 1980)
**MIYS850101**	0.0524	0.7359	2.698E-03	9.913E-03	0.0373	0.6961	2.663E-04	1.932E-03	Hydrophobicity	Effective partition energy (Miyazawa-Jernigan, 1985)
**NAGK730101**	-0.0471	1.1509	3.050E-02	6.381E-02	-0.0861	1.0762	2.058E-01	2.916E-01	Alpha and turn propensities	Normalized frequency of alpha-helix (Nagano, 1973)
**NAGK730102**	0.0461	0.7565	4.189E-04	2.713E-03	0.0369	0.7923	1.793E-03	7.212E-03	Beta propensity	Normalized frequency of bata-structure (Nagano, 1973)
**NAGK730103**	-0.0192	1.1728	1.186E-02	2.988E-02	0.0379	1.1070	8.733E-02	1.521E-01	Alpha and turn propensities	Normalized frequency of coil (Nagano, 1973)
**NAKH900101**	-0.1335	1.3094	2.218E-05	5.027E-04	-0.1335	1.2781	6.470E-05	8.819E-04	Composition	AA composition of total proteins (Nakashima et al., 1990)
**NAKH900102**	-0.1949	0.8770	1.493E-01	2.275E-01	-0.1751	0.9857	9.148E-01	9.302E-01	Composition	SD of AA composition of total proteins (Nakashima et al., 1990)
**NAKH900103**	-0.1266	1.0730	3.242E-01	4.219E-01	-0.1653	1.0617	3.869E-01	4.897E-01	Composition	AA composition of mt-proteins (Nakashima et al., 1990)
**NAKH900104**	0.0147	0.9278	5.050E-01	6.034E-01	-0.0249	0.8504	8.694E-02	1.521E-01	Hydrophobicity	Normalized composition of mt-proteins (Nakashima et al., 1990)
**NAKH900105**	-0.1168	1.0999	1.731E-01	2.566E-01	-0.1640	0.9926	8.776E-01	9.073E-01	Composition	AA composition of mt-proteins from animal (Nakashima et al., 1990)
**NAKH900106**	-0.0293	0.9777	8.357E-01	8.857E-01	-0.0467	0.8686	1.277E-01	2.056E-01	Hydrophobicity	Normalized composition from animal (Nakashima et al., 1990)
**NAKH900107**	-0.0734	1.0872	1.751E-01	2.582E-01	-0.0927	1.1574	2.194E-02	5.122E-02	Composition	AA composition of mt-proteins from fungi and plant (Nakashima et al., 1990)
**NAKH900108**	0.0191	0.8821	1.221E-01	1.931E-01	0.0099	0.8733	7.344E-02	1.323E-01	Hydrophobicity	Normalized composition from fungi and plant (Nakashima et al., 1990)
**NAKH900109**	-0.0460	1.2995	8.524E-06	3.198E-04	-0.0767	1.3048	1.484E-06	7.340E-05	Composition	AA composition of membrane proteins (Nakashima et al., 1990)
**NAKH900110**	0.0564	0.8906	1.659E-01	2.480E-01	-0.0139	0.8895	1.461E-01	2.271E-01	Hydrophobicity	Normalized composition of membrane proteins (Nakashima et al., 1990)
**NAKH900111**	-0.0290	1.0928	1.206E-01	1.913E-01	-0.0550	1.1048	8.538E-02	1.498E-01	Composition	Transmembrane regions of non-mt-proteins (Nakashima et al., 1990)
**NAKH900112**	-0.0859	1.0621	3.422E-01	4.365E-01	-0.1377	1.0634	3.200E-01	4.183E-01	Composition	Transmembrane regions of mt-proteins (Nakashima et al., 1990)
**NAKH900113**	0.0086	0.8423	4.920E-02	9.230E-02	-0.0086	0.9329	4.222E-01	5.232E-01	Hydrophobicity	Ratio of average and computed composition (Nakashima et al., 1990)
**NAKH920101**	-0.1855	1.0674	2.762E-01	3.738E-01	-0.1377	1.0443	4.163E-01	5.170E-01	Composition	AA composition of CYT of single-spanning proteins (Nakashima-Nishikawa, 1992)
**NAKH920102**	-0.1668	1.0173	6.309E-01	7.210E-01	-0.1563	0.9640	8.249E-01	8.663E-01	Composition	AA composition of CYT2 of single-spanning proteins (Nakashima-Nishikawa,
**NAKH920103**	-0.1117	1.0342	4.806E-01	5.862E-01	-0.1117	1.0057	7.685E-01	8.312E-01	Composition	AA composition of EXT of single-spanning proteins (Nakashima-Nishikawa, 1992)
**NAKH920104**	-0.0746	1.1492	8.600E-03	2.271E-02	-0.1042	1.0515	2.087E-01	2.933E-01	Composition	AA composition of EXT2 of single-spanning proteins (Nakashima-Nishikawa,
**NAKH920105**	-0.0406	1.0585	2.647E-01	3.634E-01	-0.0648	1.0447	3.437E-01	4.452E-01	Composition	AA composition of MEM of single-spanning proteins (Nakashima-Nishikawa, 1992)
**NAKH920106**	-0.1654	1.1388	2.849E-02	6.032E-02	-0.1464	1.0742	1.597E-01	2.414E-01	Composition	AA composition of CYT of multi-spanning proteins (Nakashima-Nishikawa, 1992)
**NAKH920107**	-0.0338	1.1326	5.077E-02	9.395E-02	-0.0513	1.1504	2.048E-02	4.845E-02	Composition	AA composition of EXT of multi-spanning proteins (Nakashima-Nishikawa, 1992)
**NAKH920108**	-0.0557	1.0143	7.153E-01	7.925E-01	-0.0593	1.0256	5.968E-01	6.778E-01	Composition	AA composition of MEM of multi-spanning proteins (Nakashima-Nishikawa, 1992)
**NISK800101**	0.0679	0.7100	9.164E-05	1.246E-03	0.0746	0.7003	2.244E-05	4.695E-04	Hydrophobicity	8 A contact number (Nishikawa-Ooi, 1980)
**NISK860101**	0.0474	0.6866	7.852E-05	1.228E-03	0.0414	0.6446	3.345E-06	1.213E-04	Hydrophobicity	14 A contact number (Nishikawa-Ooi, 1986)

NOZY710101	0.0574	0.7811	2.990E-03	1.043E-02	0.0027	0.7394	1.821E-04	1.478E-03	Hydrophobicity	Transfer energy, organic solvent/water (Nozaki-Tanford, 1971)
OOBM770101	-0.0510	0.7574	2.655E-03	9.892E-03	-0.0258	0.7469	9.202E-04	4.430E-03	Hydrophobicity	Average non-bonded energy per atom (Oobatake-Ooi, 1977)
OOBM770102	-0.0516	0.8508	5.433E-02	9.829E-02	0.0237	0.8661	7.254E-02	1.318E-01	Physicochemical properties	Short and medium range non-bonded energy per atom (Oobatake-Ooi, 1977)
OOBM770103	-0.0766	0.7086	3.223E-04	2.297E-03	-0.0678	0.7220	2.973E-04	2.100E-03	Hydrophobicity	Long range non-bonded energy per atom (Oobatake-Ooi, 1977)
OOBM770104	-0.0643	0.7362	5.701E-04	3.421E-03	-0.0439	0.7863	3.765E-03	1.307E-02	Physicochemical properties	Average non-bonded energy per residue (Oobatake-Ooi, 1977)
OOBM770105	-0.0511	0.8078	1.694E-02	4.008E-02	-0.0234	0.8238	2.249E-02	5.228E-02	Physicochemical properties	Short and medium range non-bonded energy per residue (Oobatake-Ooi, 1977)
OOBM850101	0.0053	0.8219	5.812E-02	1.020E-01	-0.0169	0.7893	1.672E-02	4.117E-02	Beta propensity	Optimized beta-structure-coil equilibrium constant (Oobatake et al., 1985)
OOBM850102	-0.0962	0.8778	1.873E-01	2.732E-01	-0.0562	0.8903	2.042E-01	2.901E-01	Physicochemical properties	Optimized propensity to form reverse turn (Oobatake et al., 1985)
OOBM850103	0.0089	0.9064	2.620E-01	3.608E-01	-0.0014	0.9048	2.243E-01	3.129E-01	Hydrophobicity	Optimized transfer energy parameter (Oobatake et al., 1985)
OOBM850104	0.0269	0.7457	2.119E-03	8.420E-03	0.0180	0.7806	5.506E-03	1.731E-02	Beta propensity	Optimized average non-bonded energy per atom (Oobatake et al., 1985)
OOBM850105	-0.0779	0.7517	6.132E-03	1.803E-02	-0.0468	0.8822	2.043E-01	2.901E-01	Hydrophobicity	Optimized side chain interaction parameter (Oobatake et al., 1985)
PALJ810101	-0.0564	1.1784	6.930E-03	1.974E-02	-0.0851	1.1138	6.672E-02	1.230E-01	Alpha and turn propensities	Normalized frequency of alpha-helix from LG (Palau et al., 1981)
PALJ810102	-0.0317	1.3018	6.717E-05	1.167E-03	-0.1251	1.1953	6.118E-03	1.891E-02	Alpha and turn propensities	Normalized frequency of alpha-helix from CF (Palau et al., 1981)
PALJ810103	0.0862	0.7847	9.652E-03	2.488E-02	0.0700	0.7815	5.727E-03	1.780E-02	Beta propensity	Normalized frequency of beta-sheet from LG (Palau et al., 1981)
PALJ810104	0.0728	0.7078	1.706E-04	1.760E-03	0.0586	0.7297	2.650E-04	1.932E-03	Beta propensity	Normalized frequency of beta-sheet from CF (Palau et al., 1981)
PALJ810105	0.0070	0.9515	6.129E-01	7.034E-01	0.0552	1.0146	8.019E-01	8.520E-01	Alpha and turn propensities	Normalized frequency of turn from LG (Palau et al., 1981)
PALJ810106	-0.0097	1.0865	1.095E-01	1.762E-01	0.0663	1.0714	1.515E-01	2.328E-01	Alpha and turn propensities	Normalized frequency of turn from CF (Palau et al., 1981)
PALJ810107	0.1490	0.8755	1.926E-01	2.795E-01	0.0815	1.0871	1.844E-01	2.697E-01	Alpha and turn propensities	Normalized frequency of alpha-helix in all-alpha class (Palau et al., 1981)
PALJ810108	-0.0126	1.1629	2.289E-02	5.103E-02	-0.0586	1.0444	4.136E-01	5.149E-01	Alpha and turn propensities	Normalized frequency of alpha-helix in alpha+beta class (Palau et al., 1981)
PALJ810109	-0.0855	1.1502	2.096E-02	4.785E-02	-0.1087	0.9821	8.806E-01	9.073E-01	Alpha and turn propensities	Normalized frequency of alpha-helix in alpha/beta class (Palau et al., 1981)
PALJ810110	0.0752	0.7127	1.678E-04	1.760E-03	0.0752	0.7212	1.306E-04	1.271E-03	Beta propensity	Normalized frequency of beta-sheet in all-beta class (Palau et al., 1981)
PALJ810111	0.0429	0.8393	2.529E-02	5.525E-02	0.0499	0.8339	1.519E-02	3.863E-02	Hydrophobicity	Normalized frequency of beta-sheet in alpha+beta class (Palau et al., 1981)
PALJ810112	0.0360	0.7809	6.976E-03	1.976E-02	0.0541	0.7531	9.746E-04	4.610E-03	Beta propensity	Normalized frequency of beta-sheet in alpha/beta class (Palau et al., 1981)
PALJ810113	-0.0198	0.8896	2.800E-01	3.771E-01	0.0428	1.0266	5.965E-01	6.778E-01	Alpha and turn propensities	Normalized frequency of turn in all-alpha class (Palau et al., 1981)
PALJ810114	-0.0077	0.9793	9.471E-01	9.648E-01	0.0115	0.9596	7.082E-01	7.779E-01	Alpha and turn propensities	Normalized frequency of turn in all-beta class (Palau et al., 1981)
PALJ810115	-0.0176	0.8765	2.109E-01	2.988E-01	0.0121	0.9330	5.211E-01	6.136E-01	Alpha and turn propensities	Normalized frequency of turn in alpha+beta class (Palau et al., 1981)
PALJ810116	0.0133	1.0966	1.456E-01	2.238E-01	0.0421	1.0529	3.631E-01	4.658E-01	Alpha and turn propensities	Normalized frequency of turn in alpha/beta class (Palau et al., 1981)
PARJ860101	0.0336	0.8199	3.215E-02	6.626E-02	0.0388	0.7604	2.113E-03	8.212E-03	Hydrophobicity	HPLC parameter (Parker et al., 1986)
PLIV810101	0.0222	0.7956	1.575E-02	3.824E-02	-0.0127	0.7632	3.182E-03	1.138E-02	Hydrophobicity	Partition coefficient (Pliska et al., 1981)
PONP800101	0.0479	0.6693	1.676E-05	4.341E-04	0.0527	0.6202	2.632E-07	2.386E-05	Hydrophobicity	Surrounding hydrophobicity in folded form (Ponnuswamy et al., 1980)
PONP800102	0.0842	0.6625	1.160E-05	3.741E-04	0.0854	0.6527	2.611E-06	1.014E-04	Hydrophobicity	Average gain in surrounding hydrophobicity (Ponnuswamy et al., 1980)
PONP800103	0.0829	0.7246	2.848E-04	2.199E-03	0.0728	0.7249	1.694E-04	1.418E-03	Hydrophobicity	Average gain ratio in surrounding hydrophobicity (Ponnuswamy et al., 1980)
PONP800104	0.0635	0.7752	2.650E-03	9.892E-03	0.0708	0.8213	1.657E-02	4.102E-02	Hydrophobicity	Surrounding hydrophobicity in alpha-helix (Ponnuswamy et al., 1980)
PONP800105	0.0530	0.8432	4.698E-02	8.967E-02	0.0486	0.8525	5.531E-02	1.059E-01	Hydrophobicity	Surrounding hydrophobicity in beta-sheet (Ponnuswamy et al., 1980)
PONP800106	0.0396	0.6976	1.119E-03	5.634E-03	0.0371	0.7463	4.602E-03	1.496E-02	Hydrophobicity	Surrounding hydrophobicity in turn (Ponnuswamy et al., 1980)
PONP800107	-0.0003	0.8272	2.688E-02	5.803E-02	-0.0346	0.8420	3.318E-02	7.024E-02	Hydrophobicity	Accessibility reduction ratio (Ponnuswamy et al., 1980)
PONP800108	0.0682	0.7223	2.871E-04	2.199E-03	0.0606	0.7048	5.761E-05	8.706E-04	Hydrophobicity	Average number of surrounding residues (Ponnuswamy et al., 1980)
PRAM820101	0.0087	0.9197	3.766E-01	4.710E-01	0.0433	1.1019	1.032E-01	1.738E-01	Hydrophobicity	Intercept in regression analysis (Prabhakaran-Ponnuswamy, 1982)
PRAM820102	-0.0354	0.7508	8.803E-04	4.695E-03	-0.0292	0.8284	1.749E-02	4.248E-02	Other properties	Slope in regression analysis x 1.0E1 (Prabhakaran-Ponnuswamy, 1982)
PRAM820103	-0.0395	0.9351	5.421E-01	6.383E-01	-0.0948	0.9613	7.663E-01	8.304E-01	Other properties	Correlation coefficient in regression analysis (Prabhakaran-Ponnuswamy, 1982)
PRAM900101	-0.0863	0.8165	5.439E-02	9.829E-02	-0.0019	0.7622	7.792E-03	2.304E-02	Hydrophobicity	Hydrophobicity (Prabhakaran, 1990)
PRAM900102	-0.0713	1.1462	3.141E-02	6.546E-02	-0.0858	1.0615	2.745E-01	3.714E-01	Alpha and turn propensities	Relative frequency in alpha-helix (Prabhakaran, 1990)
PRAM900103	0.0171	0.7835	3.963E-03	1.283E-02	-0.0024	0.8511	4.275E-02	8.581E-02	Beta propensity	Relative frequency in beta-sheet (Prabhakaran, 1990)
PRAM900104	-0.0161	0.9623	7.853E-01	8.452E-01	0.0192	0.9224	4.332E-01	5.320E-01	Alpha and turn propensities	Relative frequency in reverse-turn (Prabhakaran, 1990)
PTIO830101	0.0103	1.0102	7.632E-01	8.354E-01	-0.0335	0.9456	5.885E-01	6.725E-01	Alpha and turn propensities	Helix-coil equilibrium constant (Ptitsyn-Finkelstein, 1983)
PTIO830102	0.0491	0.7221	4.038E-04	2.674E-03	0.0354	0.7469	6.475E-04	3.521E-03	Beta propensity	Beta-coil equilibrium constant (Ptitsyn-Finkelstein, 1983)
QIAN880101	-0.0345	1.1542	1.634E-02	3.933E-02	-0.0264	1.1221	4.396E-02	8.761E-02	Alpha and turn propensities	Weights for alpha-helix at the window position of -6 (Qian-Sejnowski, 1988)
QIAN880102	-0.0704	1.3230	1.169E-05	3.741E-04	-0.1020	1.2202	1.681E-03	6.981E-03	Alpha and turn propensities	Weights for alpha-helix at the window position of -5 (Qian-Sejnowski, 1988)
QIAN880103	0.0238	1.3279	1.240E-05	3.748E-04	-0.0329	1.1561	2.333E-02	5.377E-02	Alpha and turn propensities	Weights for alpha-helix at the window position of -4 (Qian-Sejnowski, 1988)
QIAN880104	-0.0263	1.2237	2.457E-03	9.381E-03	-0.0860	1.2085	3.202E-03	1.138E-02	Alpha and turn propensities	Weights for alpha-helix at the window position of -3 (Qian-Sejnowski, 1988)
QIAN880105	0.0245	1.2058	4.571E-03	1.446E-02	-0.0929	1.1754	1.083E-02	2.962E-02	Alpha and turn propensities	Weights for alpha-helix at the window position of -2 (Qian-Sejnowski, 1988)
QIAN880106	-0.0147	1.2657	4.582E-04	2.899E-03	-0.0637	1.1930	7.358E-03	2.199E-02	Alpha and turn propensities	Weights for alpha-helix at the window position of -1 (Qian-Sejnowski, 1988)
QIAN880107	-0.0586	1.1663	1.203E-02	3.016E-02	-0.0867	1.1067	8.451E-02	1.488E-01	Alpha and turn propensities	Weights for alpha-helix at the window position of 0 (Qian-Sejnowski, 1988)

**QIAN880108**	0.0065	1.1297	6.960E-02	1.187E-01	-0.0105	1.0300	5.536E-01	6.439E-01	Alpha and turn propensities	Weights for alpha-helix at the window position of 1 (Qian-Sejnowski, 1988)
**QIAN880109**	0.0054	1.0019	8.077E-01	8.605E-01	-0.0332	0.9955	8.774E-01	9.073E-01	Alpha and turn propensities	Weights for alpha-helix at the window position of 2 (Qian-Sejnowski, 1988)
**QIAN880110**	-0.0092	1.1148	1.024E-01	1.668E-01	-0.0501	1.0893	1.715E-01	2.549E-01	Alpha and turn propensities	Weights for alpha-helix at the window position of 3 (Qian-Sejnowski, 1988)
**QIAN880111**	-0.0114	1.0055	7.666E-01	8.374E-01	-0.0409	0.9942	8.792E-01	9.073E-01	Alpha and turn propensities	Weights for alpha-helix at the window position of 4 (Qian-Sejnowski, 1988)
**QIAN880112**	0.0174	1.0930	2.106E-01	2.988E-01	-0.0527	1.0723	3.046E-01	4.012E-01	Alpha and turn propensities	Weights for alpha-helix at the window position of 5 (Qian-Sejnowski, 1988)
**QIAN880113**	-0.0682	0.9335	4.876E-01	5.907E-01	-0.0637	0.9527	6.658E-01	7.392E-01	Alpha and turn propensities	Weights for alpha-helix at the window position of 6 (Qian-Sejnowski, 1988)
**QIAN880114**	0.0591	0.8403	2.013E-02	4.621E-02	0.0673	0.8457	1.659E-02	4.102E-02	Hydrophobicity	Weights for beta-sheet at the window position of -6 (Qian-Sejnowski, 1988)
**QIAN880115**	0.0284	0.8135	2.197E-02	4.958E-02	0.0223	0.8062	1.141E-02	3.072E-02	Hydrophobicity	Weights for beta-sheet at the window position of -5 (Qian-Sejnowski, 1988)
**QIAN880116**	-0.0122	0.9850	8.735E-01	9.037E-01	0.0012	0.9983	9.904E-01	9.922E-01	Hydrophobicity	Weights for beta-sheet at the window position of -4 (Qian-Sejnowski, 1988)
**QIAN880117**	-0.0255	0.9361	4.820E-01	5.866E-01	0.0312	1.1305	6.835E-02	1.256E-01	Alpha and turn propensities	Weights for beta-sheet at the window position of -3 (Qian-Sejnowski, 1988)
**QIAN880118**	-0.0210	0.9834	9.680E-01	9.743E-01	0.0046	0.9397	5.164E-01	6.103E-01	Beta propensity	Weights for beta-sheet at the window position of -2 (Qian-Sejnowski, 1988)
**QIAN880119**	-0.0078	0.7601	8.890E-03	2.314E-02	-0.0383	0.7447	3.623E-03	1.280E-02	Beta propensity	Weights for beta-sheet at the window position of -1 (Qian-Sejnowski, 1988)
**QIAN880120**	0.0312	0.7207	1.148E-03	5.695E-03	0.0065	0.7350	1.136E-03	5.064E-03	Beta propensity	Weights for beta-sheet at the window position of 0 (Qian-Sejnowski, 1988)
**QIAN880121**	0.0355	0.6818	2.171E-04	1.905E-03	0.0278	0.7017	3.122E-04	2.150E-03	Beta propensity	Weights for beta-sheet at the window position of 1 (Qian-Sejnowski, 1988)
**QIAN880122**	0.0423	0.7354	6.650E-04	3.729E-03	0.0541	0.7088	8.072E-05	9.148E-04	Beta propensity	Weights for beta-sheet at the window position of 2 (Qian-Sejnowski, 1988)
**QIAN880123**	0.1052	1.0398	5.251E-01	6.236E-01	0.0904	0.9622	6.511E-01	7.245E-01	Physicochemical properties	Weights for beta-sheet at the window position of 3 (Qian-Sejnowski, 1988)
**QIAN880124**	0.0950	1.0968	1.362E-01	2.111E-01	0.1233	1.1011	1.085E-01	1.811E-01	Physicochemical properties	Weights for beta-sheet at the window position of 4 (Qian-Sejnowski, 1988)
**QIAN880125**	0.0617	0.8416	4.843E-02	9.148E-02	0.0445	0.8359	3.072E-02	6.672E-02	Physicochemical properties	Weights for beta-sheet at the window position of 5 (Qian-Sejnowski, 1988)
**QIAN880126**	0.0504	0.9189	3.027E-01	3.979E-01	0.0522	0.9051	1.960E-01	2.821E-01	Hydrophobicity	Weights for beta-sheet at the window position of 6 (Qian-Sejnowski, 1988)
**QIAN880127**	0.0329	0.8587	9.277E-02	1.534E-01	0.0293	0.9247	3.811E-01	4.846E-01	Hydrophobicity	Weights for coil at the window position of -6 (Qian-Sejnowski, 1988)
**QIAN880128**	0.0188	0.9867	9.001E-01	9.274E-01	0.0599	1.0209	7.475E-01	8.133E-01	Hydrophobicity	Weights for coil at the window position of -5 (Qian-Sejnowski, 1988)
**QIAN880129**	0.0033	1.0276	5.559E-01	6.485E-01	0.0549	0.9853	9.842E-01	9.879E-01	Alpha and turn propensities	Weights for coil at the window position of -4 (Qian-Sejnowski, 1988)
**QIAN880130**	-0.0008	1.2965	1.380E-05	3.753E-04	0.0776	1.2635	6.063E-05	8.819E-04	Alpha and turn propensities	Weights for coil at the window position of -3 (Qian-Sejnowski, 1988)
**QIAN880131**	-0.0146	0.9717	8.369E-01	8.857E-01	0.0529	0.9325	4.348E-01	5.328E-01	Alpha and turn propensities	Weights for coil at the window position of -2 (Qian-Sejnowski, 1988)
**QIAN880132**	0.0011	1.0199	7.358E-01	8.087E-01	0.0672	0.9606	7.057E-01	7.771E-01	Alpha and turn propensities	Weights for coil at the window position of -1 (Qian-Sejnowski, 1988)
**QIAN880133**	-0.0095	0.8956	3.297E-01	4.250E-01	0.0343	0.9035	3.461E-01	4.458E-01	Alpha and turn propensities	Weights for coil at the window position of 0 (Qian-Sejnowski, 1988)
**QIAN880134**	-0.0476	0.8616	1.358E-01	2.110E-01	-0.0048	0.8533	9.228E-02	1.589E-01	Alpha and turn propensities	Weights for coil at the window position of 1 (Qian-Sejnowski, 1988)
**QIAN880135**	-0.0482	0.8753	1.299E-01	2.036E-01	-0.0184	0.8726	1.010E-01	1.716E-01	Alpha and turn propensities	Weights for coil at the window position of 2 (Qian-Sejnowski, 1988)
**QIAN880136**	-0.0044	0.8845	2.908E-01	3.877E-01	0.0046	0.9167	4.598E-01	5.547E-01	Alpha and turn propensities	Weights for coil at the window position of 3 (Qian-Sejnowski, 1988)
**QIAN880137**	-0.0425	0.8419	6.142E-02	1.071E-01	-0.0161	0.7905	7.232E-03	2.198E-02	Alpha and turn propensities	Weights for coil at the window position of 4 (Qian-Sejnowski, 1988)
**QIAN880138**	0.0069	1.0132	6.085E-01	6.998E-01	0.0529	1.0047	6.943E-01	7.677E-01	Alpha and turn propensities	Weights for coil at the window position of 5 (Qian-Sejnowski, 1988)
**QIAN880139**	0.0571	0.9478	6.692E-01	7.537E-01	0.0281	0.9981	7.860E-01	8.433E-01	Alpha and turn propensities	Weights for coil at the window position of 6 (Qian-Sejnowski, 1988)
**RACS770101**	-0.0150	0.7375	1.172E-03	5.742E-03	-0.0219	0.7068	1.499E-04	1.315E-03	Hydrophobicity	Average reduced distance for C-alpha (Rackovsky-Scheraga, 1977)
**RACS770102**	-0.0306	0.7554	3.906E-03	1.272E-02	-0.0193	0.7136	4.133E-04	2.709E-03	Hydrophobicity	Average reduced distance for side chain (Rackovsky-Scheraga, 1977)
**RACS770103**	-0.0437	0.7150	9.723E-04	5.135E-03	-0.0231	0.7645	4.945E-03	1.573E-02	Hydrophobicity	Side chain orientational preference (Rackovsky-Scheraga, 1977)
**RACS820101**	-0.0429	0.8407	4.337E-02	8.426E-02	-0.0577	0.8065	9.082E-03	2.587E-02	Hydrophobicity	Average relative fractional occurrence in A0(i) (Rackovsky-Scheraga, 1982)
**RACS820102**	-0.1827	1.0943	1.054E-01	1.702E-01	-0.2105	1.0335	4.327E-01	5.320E-01	Other properties	Average relative fractional occurrence in AR(i) (Rackovsky-Scheraga, 1982)
**RACS820103**	-0.0093	0.9087	2.782E-01	3.755E-01	0.0394	0.9679	7.740E-01	8.355E-01	Hydrophobicity	Average relative fractional occurrence in AL(i) (Rackovsky-Scheraga, 1982)
**RACS820104**	-0.0029	1.5824	5.298E-12	2.882E-09	0.0473	1.4753	7.860E-10	4.276E-07	Alpha and turn propensities	Average relative fractional occurrence in EL(i) (Rackovsky-Scheraga, 1982)
**RACS820105**	0.0586	0.7395	5.012E-04	3.098E-03	0.0257	0.7829	2.261E-03	8.663E-03	Composition	Average relative fractional occurrence in E0(i) (Rackovsky-Scheraga, 1982)
**RACS820106**	0.0667	1.0820	1.975E-01	2.838E-01	0.1135	1.1301	4.774E-02	9.309E-02	Other properties	Average relative fractional occurrence in ER(i) (Rackovsky-Scheraga, 1982)
**RACS820107**	0.1472	1.4282	5.933E-09	1.614E-06	0.2058	1.3650	1.206E-07	1.640E-05	Other properties	Average relative fractional occurrence in A0(i-1) (Rackovsky-Scheraga, 1982)
**RACS820108**	-0.0103	1.2035	4.759E-03	1.488E-02	-0.0570	1.0615	2.800E-01	3.751E-01	Alpha and turn propensities	Average relative fractional occurrence in AR(i-1) (Rackovsky-Scheraga, 1982)
**RACS820109**	0.1128	1.0015	8.618E-01	8.996E-01	0.1461	1.1082	1.644E-01	2.477E-01	Other properties	Average relative fractional occurrence in AL(i-1) (Rackovsky-Scheraga, 1982)
**RACS820110**	-0.0385	0.9215	4.027E-01	5.013E-01	0.0211	0.8946	2.080E-01	2.932E-01	Alpha and turn propensities	Average relative fractional occurrence in EL(i-1) (Rackovsky-Scheraga, 1982)
**RACS820111**	-0.0057	0.9023	2.808E-01	3.772E-01	-0.0049	0.9125	3.122E-01	4.093E-01	Beta propensity	Average relative fractional occurrence in E0(i-1) (Rackovsky-Scheraga, 1982)
**RACS820112**	0.0981	0.9000	3.364E-01	4.306E-01	0.0835	0.9613	8.153E-01	8.595E-01	Alpha and turn propensities	Average relative fractional occurrence in ER(i-1) (Rackovsky-Scheraga, 1982)
**RACS820113**	0.0932	1.3317	9.037E-05	1.246E-03	0.1292	1.2486	1.582E-03	6.725E-03	Other properties	Value of theta(i) (Rackovsky-Scheraga, 1982)
**RACS820114**	0.0146	1.0959	1.789E-01	2.623E-01	0.0330	1.0679	3.001E-01	3.969E-01	Alpha and turn propensities	Value of theta(i-1) (Rackovsky-Scheraga, 1982)
**RADA880101**	0.0619	0.8564	1.470E-01	2.247E-01	0.0229	0.7854	1.651E-02	4.102E-02	Hydrophobicity	Transfer free energy from chx to wat (Radzicka-Wolfenden, 1988)
**RADA880102**	0.0562	0.8157	9.801E-03	2.503E-02	0.0482	0.7547	2.611E-04	1.932E-03	Hydrophobicity	Transfer free energy from oct to wat (Radzicka-Wolfenden, 1988)
**RADA880103**	-0.0399	0.7515	5.490E-03	1.632E-02	-0.0399	0.8186	3.542E-02	7.387E-02	Physicochemical properties	Transfer free energy from vap to chx (Radzicka-Wolfenden, 1988)
**RADA880104**	0.0716	0.8161	5.066E-02	9.395E-02	0.0504	0.8039	2.727E-02	6.105E-02	Hydrophobicity	Transfer free energy from chx to oct (Radzicka-Wolfenden, 1988)

**RADA880105**	0.0640	0.8324	3.503E-02	7.062E-02	0.0547	0.8328	2.642E-02	5.988E-02	Hydrophobicity	Transfer free energy from vap to oct (Radzicka-Wolfenden, 1988)
**RADA880106**	0.0020	0.8494	1.034E-01	1.678E-01	-0.0108	0.8538	9.452E-02	1.617E-01	Physicochemical properties	Accessible surface area (Radzicka-Wolfenden, 1988)
**RADA880107**	0.0695	0.7834	2.102E-02	4.785E-02	0.0451	0.7433	3.907E-03	1.337E-02	Hydrophobicity	Energy transfer from out to in(95%buried) (Radzicka-Wolfenden, 1988)
**RADA880108**	0.0670	0.7112	6.452E-04	3.694E-03	0.0597	0.6770	6.879E-05	8.819E-04	Hydrophobicity	Mean polarity (Radzicka-Wolfenden, 1988)
**RICJ880101**	0.0534	0.9985	8.513E-01	8.940E-01	0.0627	1.0862	1.700E-01	2.541E-01	Other properties	Relative preference value at N" (Richardson-Richardson, 1988)
**RICJ880102**	0.0534	0.9985	8.501E-01	8.940E-01	0.0627	1.0862	1.708E-01	2.546E-01	Other properties	Relative preference value at N' (Richardson-Richardson, 1988)
**RICJ880103**	0.0740	0.9569	6.300E-01	7.210E-01	0.0900	1.0061	8.182E-01	8.610E-01	Other properties	Relative preference value at N-cap (Richardson-Richardson, 1988)
**RICJ880104**	0.0341	0.8645	1.948E-01	2.818E-01	-0.0377	0.9683	7.781E-01	8.365E-01	Hydrophobicity	Relative preference value at N1 (Richardson-Richardson, 1988)
**RICJ880105**	0.0393	1.1347	8.971E-02	1.497E-01	0.0606	1.0363	5.656E-01	6.546E-01	Hydrophobicity	Relative preference value at N2 (Richardson-Richardson, 1988)
**RICJ880106**	0.0239	0.9863	9.568E-01	9.684E-01	0.0034	0.8755	2.686E-01	3.699E-01	Hydrophobicity	Relative preference value at N3 (Richardson-Richardson, 1988)
**RICJ880107**	-0.0044	1.0166	7.085E-01	7.870E-01	-0.0487	1.0310	5.686E-01	6.563E-01	Alpha and turn propensities	Relative preference value at N4 (Richardson-Richardson, 1988)
**RICJ880108**	-0.0565	0.9775	8.719E-01	9.037E-01	-0.0296	0.9736	8.069E-01	8.557E-01	Hydrophobicity	Relative preference value at N5 (Richardson-Richardson, 1988)
**RICJ880109**	0.0021	1.3155	3.116E-06	1.883E-04	-0.0461	1.2213	5.044E-04	3.154E-03	Alpha and turn propensities	Relative preference value at Mid (Richardson-Richardson, 1988)
**RICJ880110**	-0.0691	1.3076	4.695E-06	2.322E-04	-0.1083	1.2169	6.666E-04	3.555E-03	Alpha and turn propensities	Relative preference value at C5 (Richardson-Richardson, 1988)
**RICJ880111**	0.0048	0.8957	1.666E-01	2.483E-01	0.0007	0.8660	5.175E-02	9.982E-02	Hydrophobicity	Relative preference value at C4 (Richardson-Richardson, 1988)
**RICJ880112**	-0.0462	1.0423	4.965E-01	5.989E-01	-0.0648	0.9932	9.740E-01	9.794E-01	Alpha and turn propensities	Relative preference value at C3 (Richardson-Richardson, 1988)
**RICJ880113**	-0.1117	0.9256	4.093E-01	5.083E-01	-0.0992	0.8531	7.270E-02	1.318E-01	Alpha and turn propensities	Relative preference value at C2 (Richardson-Richardson, 1988)
**RICJ880114**	-0.0125	0.8508	9.474E-02	1.562E-01	-0.0076	0.9837	9.938E-01	9.938E-01	Alpha and turn propensities	Relative preference value at C1 (Richardson-Richardson, 1988)
**RICJ880115**	-0.0162	0.9657	7.933E-01	8.479E-01	0.0523	1.0330	5.847E-01	6.697E-01	Other properties	Relative preference value at C-cap (Richardson-Richardson, 1988)
**RICJ880116**	-0.1282	0.8662	1.646E-01	2.467E-01	-0.0846	0.8542	1.035E-01	1.738E-01	Alpha and turn propensities	Relative preference value at C' (Richardson-Richardson, 1988)
**RICJ880117**	0.1168	0.9858	9.595E-01	9.684E-01	0.1039	0.9693	8.147E-01	8.595E-01	Alpha and turn propensities	Relative preference value at C" (Richardson-Richardson, 1988)
**ROBB760101**	-0.0467	1.2977	1.747E-04	1.760E-03	-0.0864	1.1920	9.735E-03	2.716E-02	Alpha and turn propensities	Information measure for alpha-helix (Robson-Suzuki, 1976)
**ROBB760102**	0.0464	0.9100	3.265E-01	4.229E-01	0.0183	0.9105	2.897E-01	3.853E-01	Hydrophobicity	Information measure for N-terminal helix (Robson-Suzuki, 1976)
**ROBB760103**	-0.0125	1.2376	2.325E-03	9.033E-03	-0.0359	1.1285	7.634E-02	1.362E-01	Alpha and turn propensities	Information measure for middle helix (Robson-Suzuki, 1976)
**ROBB760104**	0.0305	0.8645	1.584E-01	2.401E-01	0.0344	0.8058	2.880E-02	6.420E-02	Alpha and turn propensities	Information measure for C-terminal helix (Robson-Suzuki, 1976)
**ROBB760105**	0.0619	0.8106	2.277E-02	5.098E-02	0.0396	0.8154	2.007E-02	4.772E-02	Beta propensity	Information measure for extended (Robson-Suzuki, 1976)
**ROBB760106**	0.0287	0.7421	3.240E-03	1.102E-02	0.0287	0.7466	2.715E-03	1.007E-02	Beta propensity	Information measure for pleated-sheet (Robson-Suzuki, 1976)
**ROBB760107**	0.0144	1.1733	1.291E-02	3.211E-02	0.0165	1.1672	1.327E-02	3.453E-02	Alpha and turn propensities	Information measure for extended without H-bond (Robson-Suzuki, 1976)
**ROBB760108**	-0.0215	1.2718	7.201E-05	1.187E-03	0.1324	1.2097	1.100E-03	4.947E-03	Alpha and turn propensities	Information measure for turn (Robson-Suzuki, 1976)
**ROBB760109**	0.0303	1.0594	3.036E-01	3.979E-01	0.0388	1.0524	3.353E-01	4.354E-01	Alpha and turn propensities	Information measure for N-terminal turn (Robson-Suzuki, 1976)
**ROBB760110**	-0.0118	1.2555	2.087E-04	1.905E-03	0.1095	1.2143	8.556E-04	4.156E-03	Alpha and turn propensities	Information measure for middle turn (Robson-Suzuki, 1976)
**ROBB760111**	0.0750	1.2296	1.367E-03	6.373E-03	0.0924	1.2631	1.227E-04	1.236E-03	Alpha and turn propensities	Information measure for C-terminal turn (Robson-Suzuki, 1976)
**ROBB760112**	0.0176	1.0026	8.582E-01	8.978E-01	0.0208	0.9760	8.560E-01	8.921E-01	Alpha and turn propensities	Information measure for coil (Robson-Suzuki, 1976)
**ROBB760113**	-0.0228	1.2157	1.560E-03	6.926E-03	0.1187	1.2091	1.239E-03	5.435E-03	Alpha and turn propensities	Information measure for loop (Robson-Suzuki, 1976)
**ROBB790101**	0.0597	0.7563	1.839E-03	7.691E-03	0.0597	0.7240	1.956E-04	1.565E-03	Hydrophobicity	Hydration free energy (Robson-Osguthorpe, 1979)
**ROSG850101**	0.0420	0.7478	3.019E-03	1.046E-02	0.0210	0.7672	4.449E-03	1.458E-02	Physicochemical properties	Mean area buried on transfer (Rose et al., 1985)
**ROSG850102**	0.0633	0.6647	5.310E-05	9.961E-04	0.0613	0.6685	3.026E-05	5.768E-04	Hydrophobicity	Mean fractional area loss (Rose et al., 1985)
**ROSM880101**	-0.0433	0.8765	2.007E-01	2.865E-01	-0.0044	0.7799	1.323E-02	3.453E-02	Hydrophobicity	Side chain hydropathy, uncorrected for solvation (Roseman, 1988)
**ROSM880102**	-0.0392	0.7762	1.300E-02	3.216E-02	-0.0037	0.7293	1.487E-03	6.371E-03	Hydrophobicity	Side chain hydropathy, corrected for solvation (Roseman, 1988)
**ROSM880103**	0.0700	0.9719	9.434E-01	9.629E-01	0.0054	1.0180	5.694E-01	6.563E-01	Hydrophobicity	Loss of Side chain hydropathy by helix formation (Roseman, 1988)
**SIMZ760101**	0.0036	0.7721	2.738E-03	9.913E-03	-0.0255	0.7531	5.808E-04	3.361E-03	Hydrophobicity	Transfer free energy (Simon, 1976), Cited by Charton-Charton (1982)
**SNEP660101**	-0.0364	1.0698	2.688E-01	3.655E-01	-0.1101	1.1028	1.187E-01	1.944E-01	Alpha and turn propensities	Principal component I (Sneath, 1966)
**SNEP660102**	-0.0108	0.9131	3.338E-01	4.282E-01	-0.0194	0.8485	5.794E-02	1.098E-01	Hydrophobicity	Principal component II (Sneath, 1966)
**SNEP660103**	0.0603	0.8476	3.004E-02	6.309E-02	0.0534	0.8615	3.797E-02	7.796E-02	Physicochemical properties	Principal component III (Sneath, 1966)
**SNEP660104**	0.0871	1.0228	6.340E-01	7.231E-01	0.0838	1.0727	2.453E-01	3.413E-01	Alpha and turn propensities	Principal component IV (Sneath, 1966)
**SUEM840101**	-0.0466	1.0729	2.719E-01	3.689E-01	-0.1035	1.0118	7.164E-01	7.825E-01	Alpha and turn propensities	Zimm-Bragg parameter s at 20 C (Sueki et al., 1984)
**SUEM840102**	0.0524	0.8464	7.341E-02	1.248E-01	-0.0244	0.8122	1.870E-02	4.500E-02	Hydrophobicity	Zimm-Bragg parameter sigma x 1.0E4 (Sueki et al., 1984)
**SWER830101**	0.0438	0.8286	3.580E-02	7.185E-02	0.0438	0.7511	1.095E-03	4.947E-03	Hydrophobicity	Optimal matching hydrophobicity (Sweet-Eisenberg, 1983)
**TANS770101**	-0.0703	1.2482	1.097E-03	5.576E-03	-0.1012	1.1475	3.714E-02	7.652E-02	Alpha and turn propensities	Normalized frequency of alpha-helix (Tanaka-Scheraga, 1977)
**TANS770102**	-0.0424	1.0212	5.527E-01	6.467E-01	-0.0581	1.0806	1.454E-01	2.267E-01	Alpha and turn propensities	Normalized frequency of isolated helix (Tanaka-Scheraga, 1977)
**TANS770103**	0.0336	1.0372	4.991E-01	6.007E-01	0.0123	0.9050	3.651E-01	4.674E-01	Beta propensity	Normalized frequency of extended structure (Tanaka-Scheraga, 1977)
**TANS770104**	-0.0752	0.7909	3.152E-02	6.546E-02	-0.0663	0.7765	1.625E-02	4.092E-02	Alpha and turn propensities	Normalized frequency of chain reversal R (Tanaka-Scheraga, 1977)
**TANS770105**	0.1836	1.1898	4.826E-03	1.500E-02	0.2304	1.1445	1.442E-02	3.700E-02	Other properties	Normalized frequency of chain reversal S (Tanaka-Scheraga, 1977)

**TANS770106**	0.0480	0.8481	5.231E-02	9.582E-02	0.0712	0.9136	2.784E-01	3.740E-01	Hydrophobicity	Normalized frequency of chain reversal D (Tanaka-Scheraga, 1977)
**TANS770107**	0.0943	1.1380	8.158E-02	1.375E-01	0.1943	1.2373	3.130E-03	1.128E-02	Other properties	Normalized frequency of left-handed helix (Tanaka-Scheraga, 1977)
**TANS770108**	-0.0357	0.9827	9.725E-01	9.743E-01	0.0138	1.0709	2.029E-01	2.897E-01	Hydrophobicity	Normalized frequency of zeta R (Tanaka-Scheraga, 1977)
**TANS770109**	0.0730	0.9122	3.531E-01	4.477E-01	0.1067	1.0769	2.012E-01	2.885E-01	Other properties	Normalized frequency of coil (Tanaka-Scheraga, 1977)
**TANS770110**	0.0109	0.9800	9.050E-01	9.290E-01	0.0658	0.9564	6.421E-01	7.202E-01	Alpha and turn propensities	Normalized frequency of chain reversal (Tanaka-Scheraga, 1977)
**VASM830101**	-0.0138	0.9956	8.498E-01	8.940E-01	-0.0070	1.0031	8.013E-01	8.520E-01	Alpha and turn propensities	Relative population of conformational state A (Vasquez et al., 1983)
**VASM830102**	-0.0471	0.9638	7.258E-01	8.016E-01	-0.0574	0.9814	9.136E-01	9.302E-01	Hydrophobicity	Relative population of conformational state C (Vasquez et al., 1983)
**VASM830103**	0.0742	0.9780	8.754E-01	9.037E-01	0.0630	0.9416	4.042E-01	5.067E-01	Hydrophobicity	Relative population of conformational state E (Vasquez et al., 1983)
**VELV850101**	0.0274	0.9775	8.632E-01	8.996E-01	0.0320	1.0462	4.303E-01	5.311E-01	Hydrophobicity	Electron-ion interaction potential (Veljkovic et al., 1985)
**VENT840101**	0.0305	0.7618	2.466E-03	9.381E-03	0.0207	0.7681	1.879E-03	7.356E-03	Hydrophobicity	Bitterness (Venanzi, 1984)
**VHEG790101**	-0.0736	0.9346	4.665E-01	5.715E-01	-0.0012	0.8900	1.785E-01	2.632E-01	Hydrophobicity	Transfer free energy to lipophilic phase (von Heijne-Blomberg, 1979)
**WARP780101**	-0.0023	0.8376	4.256E-02	8.299E-02	-0.0264	0.7973	7.902E-03	2.319E-02	Hydrophobicity	Average interactions per side chain atom (Warme-Morgan, 1978)
**WEBA780101**	-0.1438	0.7631	1.598E-03	6.954E-03	-0.0844	0.7950	4.061E-03	1.364E-02	Physicochemical properties	RF value in high salt chromatography (Weber-Lacey, 1978)
**WERD780101**	0.0679	0.7161	2.495E-04	2.055E-03	0.0501	0.7097	8.064E-05	9.148E-04	Hydrophobicity	Propensity to be buried inside (Wertz-Scheraga, 1978)
**WERD780102**	0.1192	0.9422	5.498E-01	6.446E-01	0.1291	0.9425	5.172E-01	6.103E-01	Other properties	Free energy change of epsilon(i) to epsilon(ex) (Wertz-Scheraga, 1978)
**WERD780103**	0.1260	0.9820	9.721E-01	9.743E-01	0.0796	1.0411	4.529E-01	5.487E-01	Hydrophobicity	Free energy change of alpha(Ri) to alpha(Rh) (Wertz-Scheraga, 1978)
**WERD780104**	0.0436	0.8603	9.849E-02	1.614E-01	0.0436	0.8464	5.783E-02	1.098E-01	Hydrophobicity	Free energy change of epsilon(i) to alpha(Rh) (Wertz-Scheraga, 1978)
**WOEC730101**	-0.0120	0.8873	2.599E-01	3.588E-01	0.0037	0.8179	4.171E-02	8.435E-02	Hydrophobicity	Polar requirement (Woese, 1973)
**WOLR810101**	0.0553	0.8546	6.888E-02	1.178E-01	0.0159	0.8360	2.893E-02	6.424E-02	Hydrophobicity	Hydration potential (Wolfenden et al., 1981)
**WOLS870101**	0.0110	0.8413	4.475E-02	8.602E-02	0.0316	0.8145	1.209E-02	3.209E-02	Hydrophobicity	Principal property value z1 (Wold et al., 1987)
**WOLS870102**	-0.0295	0.8538	8.882E-02	1.487E-01	-0.0168	0.8858	1.879E-01	2.733E-01	Physicochemical properties	Principal property value z2 (Wold et al., 1987)
**WOLS870103**	0.1282	1.0827	3.251E-01	4.221E-01	0.1282	1.0774	2.858E-01	3.811E-01	Alpha and turn propensities	Principal property value z3 (Wold et al., 1987)
**YUTK870101**	0.0553	0.7862	3.317E-03	1.121E-02	0.0344	0.7626	5.024E-04	3.154E-03	Hydrophobicity	Unfolding Gibbs energy in water, pH7.0 (Yutani et al., 1987)
**YUTK870102**	0.0188	0.9827	9.348E-01	9.577E-01	-0.0279	0.8797	1.443E-01	2.261E-01	Hydrophobicity	Unfolding Gibbs energy in water, pH9.0 (Yutani et al., 1987)
**YUTK870103**	0.1178	0.8675	1.261E-01	1.983E-01	0.0743	0.9453	5.779E-01	6.647E-01	Hydrophobicity	Activation Gibbs energy of unfolding, pH7.0 (Yutani et al., 1987)
**YUTK870104**	0.1079	0.8833	2.181E-01	3.074E-01	0.0812	0.9535	6.931E-01	7.677E-01	Hydrophobicity	Activation Gibbs energy of unfolding, pH9.0 (Yutani et al., 1987)
**ZASB820101**	0.0602	0.9057	2.907E-01	3.877E-01	0.0641	0.8928	1.874E-01	2.733E-01	Physicochemical properties	Dependence of partition coefficient on ionic strength (Zaslavsky et al.,
**ZIMJ680101**	-0.0386	0.8249	2.515E-02	5.517E-02	-0.0204	0.7880	4.338E-03	1.430E-02	Hydrophobicity	Hydrophobicity (Zimmerman et al., 1968)
**ZIMJ680102**	0.0167	0.8528	1.343E-01	2.100E-01	-0.0106	0.9048	3.114E-01	4.092E-01	Physicochemical properties	Bulkiness (Zimmerman et al., 1968)
**ZIMJ680103**	-0.0579	0.9005	3.035E-01	3.979E-01	-0.0266	0.8466	6.478E-02	1.199E-01	Hydrophobicity	Polarity (Zimmerman et al., 1968)
**ZIMJ680104**	-0.1693	0.8281	6.497E-02	1.122E-01	-0.1143	0.8969	2.740E-01	3.714E-01	Hydrophobicity	Isoelectric point (Zimmerman et al., 1968)
**ZIMJ680105**	-0.0516	0.9563	6.662E-01	7.519E-01	-0.0740	0.9086	2.613E-01	3.618E-01	Hydrophobicity	RF rank (Zimmerman et al., 1968)
**AURR980101**	-0.1047	0.9306	5.137E-01	6.115E-01	-0.1102	0.9207	4.062E-01	5.080E-01	Undefined	Normalized positional residue frequency at helix termini N4'(Aurora-Rose,
**AURR980102**	-0.1359	0.8120	5.606E-02	9.999E-02	-0.0896	0.9783	9.003E-01	9.241E-01	Undefined	Normalized positional residue frequency at helix termini N"' (Aurora-Rose,
**AURR980103**	-0.0339	1.0570	4.145E-01	5.124E-01	-0.0518	1.1279	8.320E-02	1.470E-01	Undefined	Normalized positional residue frequency at helix termini N" (Aurora-Rose,
**AURR980104**	-0.0176	0.8252	4.954E-02	9.262E-02	-0.0231	0.8695	1.356E-01	2.156E-01	Undefined	Normalized positional residue frequency at helix termini N'(Aurora-Rose,
**AURR980105**	0.0088	0.9013	2.656E-01	3.634E-01	-0.0068	0.9310	4.426E-01	5.399E-01	Undefined	Normalized positional residue frequency at helix termini Nc (Aurora-Rose,
**AURR980106**	-0.1128	0.9032	3.168E-01	4.133E-01	-0.1259	0.9012	2.736E-01	3.714E-01	Undefined	Normalized positional residue frequency at helix termini N1 (Aurora-Rose,
**AURR980107**	-0.0476	1.1417	1.003E-01	1.639E-01	-0.0980	1.0450	4.938E-01	5.892E-01	Undefined	Normalized positional residue frequency at helix termini N2 (Aurora-Rose,
**AURR980108**	-0.0530	1.2708	1.153E-04	1.476E-03	-0.1012	1.1364	3.492E-02	7.335E-02	Undefined	Normalized positional residue frequency at helix termini N3 (Aurora-Rose,
**AURR980109**	-0.0386	1.2155	1.338E-03	6.365E-03	-0.1061	1.0848	1.425E-01	2.240E-01	Undefined	Normalized positional residue frequency at helix termini N4 (Aurora-Rose,
**AURR980110**	-0.0882	1.2727	8.475E-05	1.246E-03	-0.0934	1.1392	2.392E-02	5.491E-02	Undefined	Normalized positional residue frequency at helix termini N5 (Aurora-Rose,
**AURR980111**	-0.0804	1.3297	2.858E-05	5.757E-04	-0.1257	1.1870	9.581E-03	2.701E-02	Undefined	Normalized positional residue frequency at helix termini C5 (Aurora-Rose,
**AURR980112**	-0.0786	1.2567	2.689E-04	2.120E-03	-0.1131	1.1579	1.041E-02	2.890E-02	Undefined	Normalized positional residue frequency at helix termini C4 (Aurora-Rose,
**AURR980113**	-0.0265	1.2533	7.614E-04	4.142E-03	-0.0929	1.1895	7.929E-03	2.319E-02	Undefined	Normalized positional residue frequency at helix termini C3 (Aurora-Rose,
**AURR980114**	-0.0675	1.0883	1.446E-01	2.228E-01	-0.0950	1.0395	4.078E-01	5.088E-01	Undefined	Normalized positional residue frequency at helix termini C2 (Aurora-Rose,
**AURR980115**	-0.1137	1.2970	6.862E-05	1.167E-03	-0.1298	1.1277	5.527E-02	1.059E-01	Undefined	Normalized positional residue frequency at helix termini C1 (Aurora-Rose,
**AURR980116**	-0.0744	1.1625	1.934E-02	4.497E-02	-0.1009	1.0808	1.891E-01	2.743E-01	Undefined	Normalized positional residue frequency at helix termini Cc (Aurora-Rose,
**AURR980117**	0.0051	0.9380	5.893E-01	6.807E-01	0.0819	1.0814	2.079E-01	2.932E-01	Undefined	Normalized positional residue frequency at helix termini C' (Aurora-Rose,
**AURR980118**	-0.0456	0.9646	8.571E-01	8.978E-01	-0.0445	0.9368	5.303E-01	6.218E-01	Undefined	Normalized positional residue frequency at helix termini C" (Aurora-Rose,
**AURR980119**	-0.1331	0.7982	3.979E-02	7.815E-02	-0.1138	0.8174	5.024E-02	9.761E-02	Undefined	Normalized positional residue frequency at helix termini C"' (Aurora-Rose,
**AURR980120**	-0.0693	0.8359	6.382E-02	1.106E-01	-0.0306	0.8864	1.812E-01	2.657E-01	Undefined	Normalized positional residue frequency at helix termini C4' (Aurora-Rose,
**ONEK900101**	0.0028	1.0918	1.920E-01	2.793E-01	-0.0114	1.0743	2.742E-01	3.714E-01	Undefined	Delta G values for the peptides extrapolated to 0 M urea (O'Neil-DeGrado,

**ONEK900102**	-0.0147	1.0197	6.851E-01	7.668E-01	-0.0231	0.9823	9.356E-01	9.443E-01	Undefined	Helix formation parameters (delta delta G) (O'Neil-DeGrado, 1990)
**VINM940101**	-0.0873	0.7039	7.902E-05	1.228E-03	-0.0630	0.7154	6.679E-05	8.819E-04	Undefined	Normalized flexibility parameters (B-values), average (Vihinen et al., 1994)
**VINM940102**	-0.0961	0.6907	3.114E-04	2.289E-03	-0.0746	0.7185	6.746E-04	3.563E-03	Undefined	Normalized flexibility parameters (B-values) for each residue surrounded by
**VINM940103**	-0.1096	0.6254	1.151E-07	2.086E-05	-0.1224	0.6493	1.784E-07	1.941E-05	Undefined	Normalized flexibility parameters (B-values) for each residue surrounded by
**VINM940104**	-0.0538	0.7814	3.880E-03	1.272E-02	-0.0459	0.7601	8.506E-04	4.156E-03	Undefined	Normalized flexibility parameters (B-values) for each residue surrounded by
**MUNV940101**	0.0344	1.0981	1.612E-01	2.436E-01	0.0456	0.9982	8.694E-01	9.026E-01	Undefined	Free energy in alpha-helical conformation (Munoz-Serrano, 1994)
**MUNV940102**	0.0438	1.2360	4.282E-03	1.362E-02	0.0756	1.0902	1.768E-01	2.613E-01	Undefined	Free energy in alpha-helical region (Munoz-Serrano, 1994)
**MUNV940103**	-0.0545	0.7058	1.090E-03	5.576E-03	-0.0471	0.7168	1.021E-03	4.789E-03	Undefined	Free energy in beta-strand conformation (Munoz-Serrano, 1994)
**MUNV940104**	-0.0600	0.8081	5.342E-02	9.752E-02	-0.0503	0.7979	3.258E-02	6.992E-02	Undefined	Free energy in beta-strand region (Munoz-Serrano, 1994)
**MUNV940105**	-0.0585	0.8419	1.183E-01	1.882E-01	-0.0332	0.8646	1.669E-01	2.502E-01	Undefined	Free energy in beta-strand region (Munoz-Serrano, 1994)
**WIMW960101**	0.0806	0.8949	2.528E-01	3.508E-01	0.0802	0.8116	1.943E-02	4.657E-02	Undefined	Free energies of transfer of AcWl-X-LL peptides from bilayer interface to
**KIMC930101**	-0.0475	0.7899	2.260E-02	5.079E-02	0.0065	0.8334	6.470E-02	1.199E-01	Undefined	Thermodynamic beta sheet propensity (Kim-Berg, 1993)
**MONM990101**	-0.0732	0.8686	1.645E-01	2.467E-01	-0.0113	0.7592	3.676E-03	1.290E-02	Undefined	Turn propensity scale for transmembrane helices (Monne et al., 1999)
**BLAM930101**	0.0276	1.0065	7.784E-01	8.435E-01	0.0166	0.9570	7.119E-01	7.793E-01	Undefined	Alpha helix propensity of position 44 in T4 lysozyme (Blaber et al., 1993)
**PARS000101**	-0.0792	0.6918	2.262E-04	1.953E-03	-0.0316	0.6744	4.067E-05	6.914E-04	Undefined	p-Values of mesophilic proteins based on the distributions of B values
**PARS000102**	-0.0973	0.8706	1.967E-01	2.838E-01	-0.0973	0.7814	1.488E-02	3.799E-02	Undefined	p-Values of thermophilic proteins based on the distributions of B values
**KUMS000101**	-0.0118	1.1451	3.401E-02	6.955E-02	-0.0228	1.1889	4.885E-03	1.572E-02	Undefined	Distribution of amino acid residues in the 18 non-redundant families of
**KUMS000102**	0.0000	1.1472	2.122E-02	4.810E-02	-0.0338	1.2321	2.237E-04	1.739E-03	Undefined	Distribution of amino acid residues in the 18 non-redundant families of
**KUMS000103**	-0.0709	1.3547	3.849E-07	4.188E-05	-0.1227	1.3235	1.127E-06	6.132E-05	Undefined	Distribution of amino acid residues in the alpha-helices in thermophilic
**KUMS000104**	-0.0418	1.3516	8.822E-07	7.998E-05	-0.1279	1.2912	1.425E-05	3.523E-04	Undefined	Distribution of amino acid residues in the alpha-helices in mesophilic
**TAKK010101**	0.0236	0.8217	2.736E-02	5.861E-02	-0.0211	0.8568	6.307E-02	1.179E-01	Undefined	Side-chain contribution to protein stability (kJ/mol) (Takano-Yutani, 2001)
**FODM020101**	0.0902	0.7153	2.100E-03	8.420E-03	0.0805	0.7154	1.329E-03	5.784E-03	Undefined	Propensity of amino acids within pi-helices (Fodje-Al-Karadaghi, 2002)
**NADH010101**	0.0850	0.7551	2.578E-03	9.740E-03	0.0758	0.7368	6.084E-04	3.472E-03	Undefined	Hydropathy scale based on self-information values in the two-state model (5%
**NADH010102**	0.0658	0.7121	4.806E-04	3.005E-03	0.0478	0.6915	7.332E-05	8.864E-04	Undefined	Hydropathy scale based on self-information values in the two-state model (9%
**NADH010103**	0.0927	0.6822	1.255E-04	1.533E-03	0.0831	0.6515	1.192E-05	3.241E-04	Undefined	Hydropathy scale based on self-information values in the two-state model (16%
**NADH010104**	0.0925	0.6378	7.376E-06	3.087E-04	0.0870	0.6344	1.955E-06	8.862E-05	Undefined	Hydropathy scale based on self-information values in the two-state model (20%
**NADH010105**	0.0959	0.6712	6.128E-05	1.111E-03	0.0920	0.6780	3.768E-05	6.612E-04	Undefined	Hydropathy scale based on self-information values in the two-state model (25%
**NADH010106**	0.1409	0.6490	2.140E-04	1.905E-03	0.1340	0.7071	1.233E-03	5.435E-03	Undefined	Hydropathy scale based on self-information values in the two-state model (36%
**NADH010107**	0.1711	0.8796	2.214E-01	3.103E-01	0.1814	0.8960	2.783E-01	3.740E-01	Undefined	Hydropathy scale based on self-information values in the two-state model (50%
**MONM990201**	0.0323	0.9153	3.720E-01	4.673E-01	0.0620	0.9021	2.706E-01	3.704E-01	Undefined	Averaged turn propensities in a transmembrane helix (Monne et al., 1999)
**KOEP990101**	0.1415	1.1411	3.822E-02	7.588E-02	0.1797	1.1244	4.631E-02	9.128E-02	Undefined	Alpha-helix propensity derived from designed sequences (Koehl-Levitt, 1999)
**KOEP990102**	-0.1055	0.7301	2.304E-04	1.958E-03	-0.1044	0.7654	8.233E-04	4.104E-03	Undefined	Beta-sheet propensity derived from designed sequences (Koehl-Levitt, 1999)
**CEDJ970101**	-0.0624	1.1767	5.454E-03	1.630E-02	-0.0782	1.1530	1.081E-02	2.962E-02	Undefined	Composition of amino acids in extracellular proteins (percent) (Cedano et
**CEDJ970102**	-0.1082	1.2668	4.062E-05	7.893E-04	-0.1081	1.2468	7.305E-05	8.864E-04	Undefined	Composition of amino acids in anchored proteins (percent) (Cedano et al.,
**CEDJ970103**	-0.0718	1.2686	2.413E-04	2.020E-03	-0.0806	1.2562	1.767E-04	1.457E-03	Undefined	Composition of amino acids in membrane proteins (percent) (Cedano et al.,
**CEDJ970104**	-0.1194	1.2317	1.050E-03	5.438E-03	-0.1188	1.2454	3.323E-04	2.260E-03	Undefined	Composition of amino acids in intracellular proteins (percent) (Cedano et
**CEDJ970105**	-0.2147	0.9067	2.905E-01	3.877E-01	-0.1825	1.0178	7.006E-01	7.731E-01	Undefined	Composition of amino acids in nuclear proteins (percent) (Cedano et al.,
**FUKS010101**	-0.1139	0.8166	4.445E-02	8.581E-02	-0.0747	0.8173	3.544E-02	7.387E-02	Undefined	Surface composition of amino acids in intracellular proteins of thermophiles
**FUKS010102**	-0.1071	0.8970	3.426E-01	4.365E-01	-0.0906	0.8485	1.163E-01	1.924E-01	Undefined	Surface composition of amino acids in intracellular proteins of mesophiles
**FUKS010103**	-0.0376	0.7323	6.273E-04	3.630E-03	-0.0341	0.8091	1.250E-02	3.300E-02	Undefined	Surface composition of amino acids in extracellular proteins of mesophiles
**FUKS010104**	-0.1209	0.7885	3.505E-02	7.062E-02	-0.1036	0.7575	9.278E-03	2.629E-02	Undefined	Surface composition of amino acids in nuclear proteins (percent)
**FUKS010105**	-0.0121	1.1811	9.124E-03	2.364E-02	-0.0546	1.1837	5.561E-03	1.739E-02	Undefined	Interior composition of amino acids in intracellular proteins of thermophiles
**FUKS010106**	-0.0208	1.1930	3.210E-03	1.098E-02	-0.0698	1.1978	1.681E-03	6.981E-03	Undefined	Interior composition of amino acids in intracellular proteins of mesophiles
**FUKS010107**	0.0026	1.1219	6.242E-02	1.085E-01	-0.0329	1.1362	3.255E-02	6.992E-02	Undefined	Interior composition of amino acids in extracellular proteins of mesophiles
**FUKS010108**	-0.0623	1.1583	2.582E-02	5.618E-02	-0.1007	1.0948	1.331E-01	2.123E-01	Undefined	Interior composition of amino acids in nuclear proteins (percent)
**FUKS010109**	-0.0888	1.1564	7.769E-03	2.121E-02	-0.1156	1.1974	6.454E-04	3.521E-03	Undefined	Entire chain composition of amino acids in intracellular proteins of
**FUKS010110**	-0.0903	1.3326	3.425E-07	4.188E-05	-0.1105	1.3625	8.100E-09	2.203E-06	Undefined	Entire chain composition of amino acids in intracellular proteins of
**FUKS010111**	0.0056	1.0217	6.808E-01	7.637E-01	-0.0458	1.0647	2.957E-01	3.923E-01	Undefined	Entire chain composition of amino acids in extracellular proteins of
**FUKS010112**	-0.0953	1.2614	1.960E-04	1.858E-03	-0.1477	1.2650	1.003E-04	1.070E-03	Undefined	Entire chain compositino of amino acids in nuclear proteins (percent)
**AVBF000101**	0.0275	0.7795	1.622E-02	3.922E-02	0.0200	0.8340	6.345E-02	1.182E-01	Undefined	Screening coefficients gamma, local (Avbelj, 2000)
**AVBF000102**	0.0173	0.9365	5.567E-01	6.485E-01	-0.0360	0.9168	3.811E-01	4.846E-01	Undefined	Screening coefficients gamma, non-local (Avbelj, 2000)
**AVBF000103**	0.0421	1.1025	1.370E-01	2.117E-01	-0.0038	0.9827	9.486E-01	9.556E-01	Undefined	Slopes tripeptide, FDPB VFF neutral (Avbelj, 2000)
**AVBF000104**	-0.0319	1.0779	3.125E-01	4.086E-01	-0.0346	0.9828	9.085E-01	9.272E-01	Undefined	Slopes tripeptides, LD VFF neutral (Avbelj, 2000)

**AVBF000105**	0.0640	1.0356	5.315E-01	6.285E-01	-0.0052	0.9484	5.827E-01	6.688E-01	Undefined	Slopes tripeptide, FDPB VFF noside (Avbelj, 2000)
**AVBF000106**	0.0747	0.9017	2.992E-01	3.950E-01	0.0389	0.8835	1.750E-01	2.594E-01	Undefined	Slopes tripeptide FDPB VFF all (Avbelj, 2000)
**AVBF000107**	-0.0325	1.0041	8.143E-01	8.652E-01	-0.0256	0.9778	8.587E-01	8.932E-01	Undefined	Slopes tripeptide FDPB PARSE neutral (Avbelj, 2000)
**AVBF000108**	-0.0087	0.9800	9.901E-01	9.901E-01	-0.0462	0.9003	3.207E-01	4.183E-01	Undefined	Slopes dekapeptide, FDPB VFF neutral (Avbelj, 2000)
**AVBF000109**	-0.0550	0.8982	2.297E-01	3.204E-01	-0.0915	0.8886	1.667E-01	2.502E-01	Undefined	Slopes proteins, FDPB VFF neutral (Avbelj, 2000)
**YANJ020101**	0.0264	0.9301	4.887E-01	5.907E-01	0.0457	0.9498	6.098E-01	6.868E-01	Undefined	Side-chain conformation by gaussian evolutionary method (Yang et al., 2002)
**MITS020101**	0.0647	0.9206	3.603E-01	4.548E-01	0.0825	0.9408	5.263E-01	6.184E-01	Undefined	Amphiphilicity index (Mitaku et al., 2002)
**TSAJ990101**	-0.0349	0.8604	5.211E-02	9.576E-02	-0.0377	0.8922	1.198E-01	1.956E-01	Undefined	Volumes including the crystallographic waters using the ProtOr (Tsai et al.,
**TSAJ990102**	-0.0315	0.8464	2.927E-02	6.171E-02	-0.0344	0.8986	1.389E-01	2.196E-01	Undefined	Volumes not including the crystallographic waters using the ProtOr (Tsai et
**COSI940101**	0.0276	0.9776	8.676E-01	9.024E-01	0.0321	1.0459	4.305E-01	5.311E-01	Undefined	Electron-ion interaction potential values (Cosic, 1994)
**PONP930101**	0.0310	0.7041	1.393E-04	1.579E-03	0.0274	0.6801	1.344E-05	3.483E-04	Undefined	Hydrophobicity scales (Ponnuswamy, 1993)
**WILM950101**	0.0289	0.8154	2.774E-02	5.895E-02	0.0078	0.8107	1.742E-02	4.248E-02	Undefined	Hydrophobicity coefficient in RP-HPLC, C18 with 0.1%TFA/MeCN/H2O (Wilce et
**WILM950102**	0.0185	0.8077	2.455E-02	5.407E-02	-0.0126	0.8685	1.177E-01	1.934E-01	Undefined	Hydrophobicity coefficient in RP-HPLC, C8 with 0.1%TFA/MeCN/H2O (Wilce et al.
**WILM950103**	0.0827	1.2308	1.734E-03	7.369E-03	0.0590	1.2190	1.855E-03	7.356E-03	Undefined	Hydrophobicity coefficient in RP-HPLC, C4 with 0.1%TFA/MeCN/H2O (Wilce et al.
**WILM950104**	0.0000	1.0103	7.357E-01	8.087E-01	0.0017	1.0113	7.342E-01	8.004E-01	Undefined	Hydrophobicity coefficient in RP-HPLC, C18 with 0.1%TFA/2-PrOH/MeCN/H2O
**KUHL950101**	-0.0774	0.8714	1.752E-01	2.582E-01	-0.0471	0.7277	1.062E-03	4.853E-03	Undefined	Hydrophilicity scale (Kuhn et al., 1995)
**GUOD860101**	0.0184	0.7997	1.646E-02	3.945E-02	-0.0166	0.7884	7.338E-03	2.199E-02	Undefined	Retention coefficient at pH 2 (Guo et al., 1986)
**JURD980101**	0.0564	0.8506	5.603E-02	9.999E-02	0.0329	0.7422	4.532E-04	2.935E-03	Undefined	Modified Kyte-Doolittle hydrophobicity scale (Juretic et al., 1998)
**BASU050101**	0.0423	0.7447	2.744E-03	9.913E-03	0.0275	0.6699	4.490E-05	7.020E-04	Undefined	Interactivity scale obtained from the contact matrix (Bastolla et al., 2005)
**BASU050102**	0.0528	0.6736	3.033E-04	2.260E-03	0.0477	0.6511	4.499E-05	7.020E-04	Undefined	Interactivity scale obtained by maximizing the mean of correlation
**BASU050103**	0.0708	0.7021	9.977E-05	1.324E-03	0.0624	0.6734	6.391E-06	2.045E-04	Undefined	Interactivity scale obtained by maximizing the mean of correlation
**SUYM030101**	0.1069	0.8493	1.046E-01	1.694E-01	0.0951	0.8429	7.559E-02	1.353E-01	Undefined	Linker propensity index (Suyama-Ohara, 2003)
**PUNT030101**	-0.0248	0.8289	5.462E-02	9.839E-02	-0.0022	0.8024	1.643E-02	4.102E-02	Undefined	Knowledge-based membrane-propensity scale from 1D_Helix in MPtopo databases
**PUNT030102**	-0.0428	0.7846	5.304E-03	1.614E-02	-0.0003	0.7526	6.190E-04	3.472E-03	Undefined	Knowledge-based membrane-propensity scale from 3D_Helix in MPtopo databases
**GEOR030101**	-0.1057	1.0249	5.261E-01	6.236E-01	-0.1216	1.0260	4.582E-01	5.539E-01	Undefined	Linker propensity from all dataset (George-Heringa, 2003)
**GEOR030102**	-0.0937	0.9043	3.294E-01	4.250E-01	-0.1109	0.9733	9.217E-01	9.355E-01	Undefined	Linker propensity from 1-linker dataset (George-Heringa, 2003)
**GEOR030103**	-0.0874	0.9847	9.501E-01	9.650E-01	-0.1492	1.0060	8.280E-01	8.679E-01	Undefined	Linker propensity from 2-linker dataset (George-Heringa, 2003)
**GEOR030104**	-0.0985	1.2832	1.318E-04	1.533E-03	-0.1549	1.2268	1.059E-03	4.853E-03	Undefined	Linker propensity from 3-linker dataset (George-Heringa, 2003)
**GEOR030105**	-0.0834	0.8144	5.773E-02	1.016E-01	-0.0984	0.8613	1.568E-01	2.389E-01	Undefined	Linker propensity from small dataset (linker length is less than six
**GEOR030106**	-0.1093	1.1634	2.439E-02	5.394E-02	-0.1565	1.1476	2.696E-02	6.060E-02	Undefined	Linker propensity from medium dataset (linker length is between six and 14
**GEOR030107**	-0.0449	0.8887	1.675E-01	2.489E-01	-0.0459	0.8849	1.294E-01	2.076E-01	Undefined	Linker propensity from long dataset (linker length is greater than 14
**GEOR030108**	-0.0761	1.2830	2.569E-04	2.055E-03	-0.1529	1.2151	2.998E-03	1.094E-02	Undefined	Linker propensity from helical (annotated by DSSP) dataset (George-Heringa,
**GEOR030109**	-0.0381	0.9946	9.046E-01	9.290E-01	-0.0414	0.9116	3.813E-01	4.846E-01	Undefined	Linker propensity from non-helical (annotated by DSSP) dataset
**ZHOH040101**	0.0688	0.7440	7.819E-03	2.121E-02	0.0603	0.6919	7.257E-04	3.796E-03	Undefined	The stability scale from the knowledge-based atom-atom potential (Zhou-Zhou,
**ZHOH040102**	0.0193	0.7678	6.533E-03	1.871E-02	0.0023	0.7252	7.484E-04	3.805E-03	Undefined	The relative stability scale extracted from mutation experiments (Zhou-Zhou,
**ZHOH040103**	0.0914	0.6703	8.260E-05	1.246E-03	0.0652	0.6600	2.535E-05	5.108E-04	Undefined	Buriability (Zhou-Zhou, 2004)
**BAEK050101**	0.1005	0.6884	3.268E-04	2.297E-03	0.0913	0.6929	2.361E-04	1.784E-03	Undefined	Linker index (Bae et al., 2005)
**HARY940101**	-0.0042	0.7457	3.796E-04	2.594E-03	-0.0057	0.8453	2.576E-02	5.864E-02	Undefined	Mean volumes of residues buried in protein interiors (Harpaz et al., 1994)
**PONJ960101**	0.0063	0.7934	7.410E-03	2.073E-02	-0.0063	0.8427	3.644E-02	7.556E-02	Undefined	Average volumes of residues (Pontius et al., 1996)
**DIGM050101**	-0.1699	1.0005	7.814E-01	8.435E-01	-0.1134	1.0161	6.442E-01	7.211E-01	Undefined	Hydrostatic pressure asymmetry index, PAI (Di Giulio, 2005)
**WOLR790101**	0.0311	0.8416	4.836E-02	9.148E-02	0.0031	0.8380	3.054E-02	6.671E-02	Undefined	Hydrophobicity index (Wolfenden et al., 1979)
**OLSK800101**	0.0480	0.8152	1.315E-02	3.228E-02	0.0189	0.8084	6.723E-03	2.066E-02	Undefined	Average internal preferences (Olsen, 1980)
**KIDA850101**	-0.0511	0.8144	3.444E-02	7.016E-02	-0.0309	0.8056	1.866E-02	4.500E-02	Undefined	Hydrophobicity-related index (Kidera et al., 1985)
**GUYH850102**	-0.0522	0.7115	3.227E-04	2.297E-03	-0.0429	0.7109	1.661E-04	1.412E-03	Undefined	Apparent partition energies calculated from Wertz-Scheraga index (Guy, 1985)
**GUYH850103**	-0.0580	0.7308	1.556E-03	6.926E-03	-0.0542	0.6994	1.455E-04	1.297E-03	Undefined	Apparent partition energies calculated from Robson-Osguthorpe index (Guy,
**GUYH850104**	-0.0635	0.7226	1.346E-03	6.365E-03	-0.0377	0.7221	8.109E-04	4.085E-03	Undefined	Apparent partition energies calculated from Janin index (Guy, 1985)
**GUYH850105**	-0.0983	0.7607	3.862E-03	1.272E-02	-0.0521	0.7758	4.220E-03	1.400E-02	Undefined	Apparent partition energies calculated from Chothia index (Guy, 1985)
**ROSM880104**	0.0234	0.8166	1.023E-02	2.592E-02	-0.0001	0.8179	7.312E-03	2.199E-02	Undefined	Hydropathies of amino acid side chains, neutral form (Roseman, 1988)
**ROSM880105**	0.0547	0.8136	5.106E-02	9.416E-02	0.0122	0.7809	1.189E-02	3.186E-02	Undefined	Hydropathies of amino acid side chains, pi-values in pH 7.0 (Roseman, 1988)
**JACR890101**	0.0973	0.8357	8.161E-02	1.375E-01	0.0598	0.7570	4.988E-03	1.578E-02	Undefined	Weights from the IFH scale (Jacobs-White, 1989)
**COWR900101**	0.0125	0.7230	5.489E-04	3.355E-03	-0.0127	0.7083	1.310E-04	1.271E-03	Undefined	Hydrophobicity index, 3.0 pH (Cowan-Whittaker, 1990)
**BLAS910101**	0.0605	0.8588	1.351E-01	2.107E-01	0.0355	0.7533	4.028E-03	1.361E-02	Undefined	Scaled side chain hydrophobicity values (Black-Mould, 1991)
**CASG920101**	0.0906	0.6838	4.257E-04	2.724E-03	0.0940	0.6691	1.098E-04	1.127E-03	Undefined	Hydrophobicity scale from native protein structures (Casari-Sippl, 1992)

**CORJ870101**	0.0820	0.7222	3.294E-04	2.297E-03	0.0842	0.6602	3.694E-06	1.256E-04	Undefined	NNEIG index (Cornette et al., 1987)
**CORJ870102**	0.0444	0.8343	4.007E-02	7.842E-02	0.0435	0.7494	8.298E-04	4.104E-03	Undefined	SWEIG index (Cornette et al., 1987)
**CORJ870103**	0.0186	0.7507	2.256E-03	8.831E-03	0.0221	0.7285	5.255E-04	3.249E-03	Undefined	PRIFT index (Cornette et al., 1987)
**CORJ870104**	0.0299	0.7458	4.169E-03	1.337E-02	0.0210	0.7344	1.869E-03	7.356E-03	Undefined	PRILS index (Cornette et al., 1987)
**CORJ870105**	0.0226	0.7521	3.156E-03	1.087E-02	-0.0218	0.7553	2.410E-03	9.169E-03	Undefined	ALTFT index (Cornette et al., 1987)
**CORJ870106**	0.0274	0.7224	2.888E-03	1.022E-02	-0.0062	0.7214	1.646E-03	6.940E-03	Undefined	ALTLS index (Cornette et al., 1987)
**CORJ870107**	0.0294	0.7247	6.975E-04	3.872E-03	-0.0060	0.7058	1.357E-04	1.271E-03	Undefined	TOTFT index (Cornette et al., 1987)
**CORJ870108**	-0.0219	0.7478	3.756E-03	1.246E-02	0.0018	0.6953	2.188E-04	1.725E-03	Undefined	TOTLS index (Cornette et al., 1987)
**MIYS990101**	-0.0361	0.7393	1.627E-03	7.026E-03	-0.0147	0.7267	5.453E-04	3.284E-03	Undefined	Relative partition energies derived by the Bethe approximation
**MIYS990102**	-0.0390	0.7302	1.228E-03	5.912E-03	-0.0170	0.7149	3.496E-04	2.348E-03	Undefined	Optimized relative partition energies - method A (Miyazawa-Jernigan, 1999)
**MIYS990103**	-0.0542	0.6859	1.793E-04	1.774E-03	-0.0381	0.6483	1.095E-05	3.134E-04	Undefined	Optimized relative partition energies - method B (Miyazawa-Jernigan, 1999)
**MIYS990104**	-0.0791	0.6825	8.727E-05	1.246E-03	-0.0545	0.6527	7.082E-06	2.140E-04	Undefined	Optimized relative partition energies - method C (Miyazawa-Jernigan, 1999)
**MIYS990105**	-0.0573	0.7026	2.108E-04	1.905E-03	-0.0518	0.7013	9.180E-05	9.988E-04	Undefined	Optimized relative partition energies - method D (Miyazawa-Jernigan, 1999)
**ENGD860101**	-0.0856	0.8163	5.417E-02	9.829E-02	-0.0019	0.7629	7.696E-03	2.288E-02	Undefined	Hydrophobicity index (Engelman et al., 1986)
**FASG890101**	-0.0808	0.7064	1.324E-04	1.533E-03	-0.0790	0.6921	3.075E-05	5.768E-04	Undefined	Hydrophobicity index (Fasman, 1989)
